# A generalizable data-driven multicellular model of pancreatic ductal adenocarcinoma

**DOI:** 10.1093/gigascience/giaa075

**Published:** 2020-07-22

**Authors:** Boris Aguilar, David L Gibbs, David J Reiss, Mark McConnell, Samuel A Danziger, Andrew Dervan, Matthew Trotter, Douglas Bassett, Robert Hershberg, Alexander V Ratushny, Ilya Shmulevich

**Affiliations:** Institute for Systems Biology, 401 Terry Avenue North, Seattle, WA 98109, USA; Institute for Systems Biology, 401 Terry Avenue North, Seattle, WA 98109, USA; Bristol-Myers Squibb, 400 Dexter Avenue North, Suite 1200, Seattle, WA 98109, USA; Bristol-Myers Squibb, 400 Dexter Avenue North, Suite 1200, Seattle, WA 98109, USA; Bristol-Myers Squibb, 400 Dexter Avenue North, Suite 1200, Seattle, WA 98109, USA; Bristol-Myers Squibb, 400 Dexter Avenue North, Suite 1200, Seattle, WA 98109, USA; BMS Center for Innovation and Translational Research Europe (CITRE), Pabellon de Italia, Calle Isaac Newton 4, Sevilla 41092, Spain; Bristol-Myers Squibb, 400 Dexter Avenue North, Suite 1200, Seattle, WA 98109, USA; Formerly Celgene Corporation, 400 Dexter Avenue North, Suite 1200, Seattle, WA 98109, USA; Bristol-Myers Squibb, 400 Dexter Avenue North, Suite 1200, Seattle, WA 98109, USA; Institute for Systems Biology, 401 Terry Avenue North, Seattle, WA 98109, USA

**Keywords:** cancer modeling, data-driven model, pancreatic ductal adenocarcinoma, multicellular model

## Abstract

**Background:**

Mechanistic models, when combined with pertinent data, can improve our knowledge regarding important molecular and cellular mechanisms found in cancer. These models make the prediction of tissue-level response to drug treatment possible, which can lead to new therapies and improved patient outcomes. Here we present a data-driven multiscale modeling framework to study molecular interactions between cancer, stromal, and immune cells found in the tumor microenvironment. We also develop methods to use molecular data available in The Cancer Genome Atlas to generate sample-specific models of cancer.

**Results:**

By combining published models of different cells relevant to pancreatic ductal adenocarcinoma (PDAC), we built an agent-based model of the multicellular pancreatic tumor microenvironment, formally describing cell type–specific molecular interactions and cytokine-mediated cell-cell communications. We used an ensemble-based modeling approach to systematically explore how variations in the tumor microenvironment affect the viability of cancer cells. The results suggest that the autocrine loop involving EGF signaling is a key interaction modulator between pancreatic cancer and stellate cells. EGF is also found to be associated with previously described subtypes of PDAC. Moreover, the model allows a systematic exploration of the effect of possible therapeutic perturbations; our simulations suggest that reducing bFGF secretion by stellate cells will have, on average, a positive impact on cancer apoptosis.

**Conclusions:**

The developed framework allows model-driven hypotheses to be generated regarding therapeutically relevant PDAC states with potential molecular and cellular drivers indicating specific intervention strategies.

## Introduction

Pancreatic ductal adenocarcinoma (PDAC), the most common form of pancreatic cancer, is the fourth leading cause of cancer-associated death in the United States and is predicted to be the second in 2030 [1]. With a 5-year survival rate of only 3%, it has a poor prognosis. Across all types of cancer, it is becoming increasingly clear that interactions within the tumor microenvironment (TME) have a strong effect on tumor growth. This is particularly relevant for PDAC research, where previous studies have revealed high heterogeneity and complexity in the TME, where a mixture of interacting immune cells, stromal tissue, and cancer cells coexist. However, much remains to be learned regarding how differences in the TME affect the behavior of cancer cells. For instance, there is a debate concerning whether stroma-cancer interactions are associated with progression of pancreatic cancer or, rather, provide protective measures [2]. Thus, to make progress in the treatment of PDAC, new strategies must be developed to improve our understanding of the effects of the TME on cancer states and progression.

*In silico* models are frequently used in systems biology for the discovery of general principles and novel hypotheses [3–5]. Moreover, it is eventually possible that when combined with relevant data, *in silico* models will be able to make predictions with sufficient accuracy for therapeutic treatment. Despite their potential, concrete examples of predictive models of cancer progression are scarce. One reason is that most models have focused on single–cell type dynamics, ignoring the interactions between cancer cells and their local microenvironment. Indeed, there have been a number of models that were used to study gene regulation at the single-cell scale, such as macrophage differentiation [6–8], T cell exhaustion [9], differentiation and plasticity of T helper cells [10, 11], cell cycle [12–14], and regulation of key genes in different tumor types [15].

Although not as numerous as single cell–type models, multicellular models have progressively been developed to study different aspects of cancer biology, including tumor immunosurveillance [16–20], hypoxia [21, 22], angiogenesis [23, 24], and epithelial-mesenchymal transition [25, 26], among others; we refer the reader to Metzcar et al. [27] for a recent and comprehensive review. Typically, these models are based on phenomenological rules to model cell behavior and therefore use limited data to calibrate their parameters. Although multicellular models are being increasingly used in cancer biology, there remains a need for a modeling framework that is capable of integrating different multiscale properties of the TME, such as molecular and cellular heterogeneity and non-uniform spatial distributions of cells, with the capacity to leverage diverse -omics datasets for model building, calibration, and validation, allowing researchers to explore novel molecular therapies *in silico* [3, 28–30].

**Figure 1:**
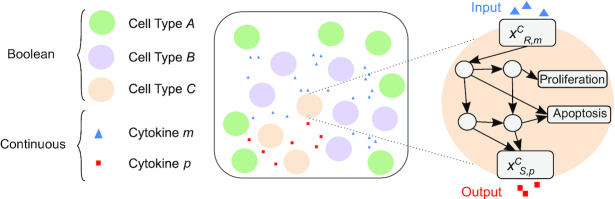
Schematic representation of the multiscale model including multiple cell types and cytokines of the TME.

In this work, we developed a modeling framework designed to study the interaction between cancer cells and their microenvironment. Fig. 1 shows a schematic of the modeling framework. The framework is a combination of two well-established approaches: Boolean networks [31] (BNs) and agent-based modeling [27] (ABM), used at the molecular and cellular levels, respectively. The cancer signaling and regulatory networks are modeled with BNs, while ABM is used to simulate intercellular networks consisting of different cell types and intercellular signaling molecules. We used BNs because of their efficient and simple formulation that minimizes the number of parameters in the multicellular model. This vertical (“multiscale”) integration, using ABM and BNs, enables the exploration of therapeutic interventions on the molecular level for inducing transitions of the tumor into less proliferative states, while using currently available high-throughput molecular data.

Voukantsis et al. [32] proposed a multicellular model for tumor growth in which cells are placed in a lattice. Each cell is endowed with a Boolean network that controls cellular actions, such as proliferation and apoptosis, that are key for tumor growth. Letort et al. [33] integrated stochastic Boolean signaling networks into ABMs by combining MaBoSS [34, 35], an open source package for BNs, with PhysiCell [17], an ABM-based simulation platform. The main goal of the previous ABM/BN combinations was the simulation of tumor growth, which requires not only parameters that regulate cell-cell communication and intracellular gene regulation but also parameters for cell division, cell death, oxygen uptake, mechanical interactions, extracellular matrix properties, and so forth, resulting in highly complex models that require data currently not available for validation and calibration [36]. In this article, our focus is modeling how the cancer cell state is affected by communication with other cells in the TME. Therefore, we included model components, such as gene regulation, cell proportions, and cellular spatial distributions, that can be directly compared with commonly used omics and imaging data, aiming at integration between the model and experimental data needed in cancer research [28].

We built a network of cell type–specific intracellular interactions and cytokine-mediated intercellular communications by combining published models of different cell types relevant to PDAC, namely, the ductal cancer cells, stellate cells, CD4^+^ T cells, CD8^+^ T cells, and macrophages. Through computational simulations, using an ensemble modeling approach whereby multiple simulations are aggregated into statistically summarized results, this framework was used to study how the TME, characterized by a set of cytokines, stromal cells, and somatically heterogeneous cancer cells, affects the viability of cancer cells.

## Modeling Framework

In this section, we describe our approach to model a block of cancerous tissue with a mixture of cancer, stromal, and immune cells randomly located inside a 3D rectangular simulation domain (Fig. 1). Each cell contains a BN that determines its cellular phenotype (functional state), such as proliferation or apoptosis, the possible secretion of cytokines, and the state of membrane receptors. The model is built on the following assumptions and considerations:

- Because our main goal was to study the interplay between cell-cell communication and gene regulation, other interactions and processes, such as cell motility and mechanical interactions, were not included in the model. Moreover, the model simulations focus on a time window relevant to cell signaling and gene regulation, which is a few hours. Considering these time scales, we assumed that the number of cells and the initial positions of cells do not change during simulations.
- The model uses two time scales, one for gene regulation and one for cell-cell communication. Although they are biologically related, we assume that cell communication takes place on a faster time scale than gene regulation.
- The parameters that characterize cell behavior are the same for all cells of a given type. Thus, all cells of a single type are governed by the same BN and share the same parameters of cell communication.

The following subsections present a detailed description of each component of the modeling approach.

### Cells as Boolean networks

Signal transduction and gene regulation in a given cell are modeled with synchronous BNs, a well-known modeling approach used to study several cellular processes important in cancer [37, 38]. It is termed synchronous because all nodes in the BN (in all cells) are updated simultaneously at each time step. The BN of a cell *i* is defined on a set of *n* binary-valued variables ${X_i} = \ \{ {x_1^i,\ \ldots ,\ x_n^i} \}$, where a node $x_j^i \in \{ {0,1} \}$ represents the expression of a gene, a cellular behavior, or secretion of a cytokine to the TME. The binary vector ${X_i}$ represents the phenotypic state of cell *i*. Thus, for a cellular BN of *n* nodes, there are ${2^n}$ possible states. We divided the binary nodes $x_j^i$ into 2 groups: signal receptors and regulatory nodes. Receptor nodes sense the presence of signaling molecules in the local TME, with their updating rules being specified in the next subsection. Regulatory nodes are updated in discrete time steps by conventional logic rules. Specifically, the regulatory node *j* of a cell *i* at time step $t + 1$ (i.e., the next time step) is determined by the values of the nodes (“genes”), $x_{{j_1}}^i,\ x_{{j_2}}^i,\ \ldots ,\ x_{{j_{{k_{j,i}}}}}^i$ at time *t* by means of the Boolean function $F_j^i:\ {\{ 0,\ 1\} ^{{k_{j,i}}}} \to \{ {0,\ 1} \}$. There are ${k_{j,i}}$ nodes assigned as inputs to regulatory node $x_j^i$, thereby determining the wiring of the BN. Thus, the Boolean value of a regulatory node $x_j^i$ is given by

(1) \begin{equation*}
x_j^i\left( {t + 1} \right)\ = \ F_j^i\left( {x_{{j_1}}^i\left( t \right),\ \ldots ,x_{{j_{{k_{j,i}}}}}^i\left( t \right)} \right). \end{equation*}

It is worth noting that regulatory genes of all cells are updated synchronously using the states of nodes of the same cell, whereas membrane receptors are updated by the TME, i.e., by the presence of cytokines right before the update of regulatory genes. Moreover, cells of the same type are regulated by the same set of Boolean functions. Thus, all cells of type *I* are regulated by $\{ F_1^I,\ F_2^I,\ \ldots \} $, which do not change during simulations. These regulatory functions represent existing knowledge of intracellular gene regulation in a given cell type and are typically obtained from the literature.

Additionally, to model stochastic dynamics, following the convention used in random BNs [31, 39, 40], we introduce a perturbation probability *q* and a random perturbation vector, $\gamma \ = \ [ {{\gamma _1},\ \ {\gamma _2},\ \ldots ,\ {\gamma _n}} ],$ where ${\gamma _j} \in \{ {0,1} \}$ and $P\{ {\ {\gamma _j} = \ 1} \}\ = \ q$, such that

\begin{equation*} \begin{array}{@{}*{1}{c}@{}} {{X_i}\left( {t + 1} \right) = {X_i}\left( t \right) \oplus \gamma ,\,\,{\mathrm{with\ probability}}\,\,1 - {{(1 - q)}^n}}\\ {{X_i}\left( {t + 1} \right) = \left[ {F_1^i,\ F_2^i,\ \ldots ,F_n^i} \right],\,\,{\mathrm{otherwise}},} \end{array} \end{equation*}

where $\oplus $ indicates the modulo-2 sum. The fact that any state transition has a nonzero probability under this perturbation model implies that the dynamics of the network are described by an ergodic Markov chain with a (unique) steady-state distribution [40, 41]. It is worth noting that we use the same gamma value $({\gamma _j} = \ q)$ for all the genes regardless of the cell type.

Some of the regulatory nodes are associated with important cellular behaviors, such as proliferation, apoptosis, or migration. Moreover, some of the regulatory nodes are associated with the secretion of cytokines in such a way that a state of 0 or 1 of these nodes corresponds to low or high rates of secretion, respectively.

### Cell-cell communication via diffusion of cytokines

We include communication between cells by modeling the secretion, sensing, and diffusion of cytokines. The formulation of cell-cell communication is similar to the model developed by Olimpio et al. [42]. For simplicity we made the following assumptions. First, the concentration of cytokines is not affected by cellular uptake of molecules. Second, the cytokine diffusion is much faster than gene regulation.

A cell *i* releases cytokine $m\ $ with a secretion rate of $\eta _m^i( {x_{{S_m}}^{\ i}} )$ molecules per time step, which depends on the Boolean state of its designated signal node $x_{{S_m}}^{\ i}$ (${S_m}$ is the label of one of the regulatory nodes of cell *i*). We assume that $\eta _m^i( 0 )\ = \ 1$ and $\eta _m^i( 1 )\ = R_m^i\ $, $R_m^i > 1$, to account for basal and active expression, respectively. We make this assumption with no loss of generality because it is equivalent to normalizing active expression by the lower basal expression [42]. The concentration, *C*, of cytokine *m* changes in space and time according to a diffusion degradation equation. For cells randomly scattered in a regular 3D lattice, the concentration of cytokine *m* in a voxel *v* is approximated by solving the following diffusion degradation equation with periodic boundary conditions:

(2) \begin{equation*}
\partial C_m^v/\partial t\ = \ D\vartriangle C_m^v\ - {\gamma _D}C_m^v\ + {h^{ - 3}}\mathop \sum \nolimits_{i \in v} \eta _m^i\left( {x_{{S_m}}^i} \right) \end{equation*}

for each voxel *v* of the lattice containing a set of cells. *D* is the diffusion coefficient, ${\gamma _D}$ is the constant degradation rate, and *h* is grid spacing used to solve the diffusion degradation equation by the finite difference method. Assuming that diffusion is much faster than gene regulation, we use the steady state of the diffusion equation above,

(3) \begin{equation*}
0\ = \ D\vartriangle C_m^v\ - {\gamma _D}C_m^v\ + {h^{ - 3}}\mathop \sum \nolimits_{i \in v} \eta _m^i\left( {x_{{S_m}}^i} \right), \end{equation*}

and use a numerical solver for calculating $C_m^v$ in the simulations. An important component of the steady state solution is the effective interaction distance, $\lambda $, where $\lambda \ = (D/\gamma _D)^{1/2} \ \ $ [43, 44].

### Integration of gene regulation and cell-cell communication

The coupling between signal diffusion and BNs was adapted from Olimpio et al. [42], where a cellular automata model was used to analyze the consequences of cell-cell communication. Figure 1 shows a representation of the integration between BNs and cell-to-cell signaling. The cellular BNs can influence the spatial distribution of cytokines. A cytokine *p* is secreted by cell *i* of type *C* with secretion rate $R_p^C$ (high) or 1 (low) according to the Boolean state of an output node of its BN, $x_{S,\ p}^C$, in Fig. 1.

The concentration of cytokines can influence the behavior of cellular BNs. To sense cytokine *m*, cell *i* checks the local concentration of the signal, i.e., the concentration at its containing voxel. If the local concentration of *m* is above a threshold value, $K_m^i$, then the signal receptor is activated; otherwise it is deactivated. This is depicted by the blue triangles in Fig. 1. Formally, the state of the receptor node $x_{R,\ m}^{\ i}$ of cell *i*, located in voxel *v*, follows the equations:

(4) \begin{equation*} \begin{array}{@{}*{1}{l}@{}} {x_{R,m}^{\ i}\left( {t + 1} \right) = 1,\ \,\,\mathrm{if}\,\,C_m^v\left( t \right) > K_m^i,}\\ {x_{R,m}^{\ i}\left( {t + 1} \right) = 0,\,\,{\mathrm{otherwise}},} \end{array}
\end{equation*}

where $C_m^v$ is the concentration of *m* in voxel *v* that contains cell *i*. The thresholds $K_m^i$ are parameters of the model that characterize the sensitivity of cells to cytokine concentration. All cells of the same type share the same activation threshold associated with a given cytokine.

Note that while our model assumes diffusion-based cell-cell communication, the effective interaction distance can be shortened, such that the system behaves as if signaling were contact-mediated, the latter effectively being a special case of the former. This is possible by setting a spacing resolution (*h*) equal to the cell diameter, such that changes in concentration between nearest-neighbor cells can be captured by the model of signal diffusion.

### Tissue architecture

We constructed a lattice-free model tissue as a 3D point process of cells, each represented by a BN and a spatial point in a rectangular block of size *L*. We assume a fixed density of cells, ${\mathrm{\rho }}$$,$ and divide cell types into cancer and stromal. The density of cancer cells is ${{\mathrm{\rho }}_C} = {r_C}{\mathrm{\rho }}$, where ${r_C}$ is the fraction of cancer cells in the tissue sample. The density of stromal cells is ${{\mathrm{\rho }}_S} = ( {1 - {r_C}} ){\mathrm{\rho }}$. The positions of cancer cells were generated by a Thomas process [45] in which points are scattered around cluster centers according to a 3D Gaussian distribution with zero mean and covariance matrix ${\sigma ^2}I$, where *I* is the 3 $\times $ 3 identity matrix. The cluster centers are generated by a simple Poisson process with intensity ${{\mathrm{\rho }}_{cc}}$. Stromal cells are generated by a void process [46] in which points are removed if they are within a distance ${R_{\mathrm{ex}}}$ from a cluster center. The same cluster centers were used for cancer and stromal cells. The cluster centers are generated using a Poisson process with density ${{\mathrm{\rho }}_{\mathrm{cc}}} = s{{\mathrm{\rho }}_C}$, where *s* is a parameter that determines the clustering of cancer cells.

**Figure 2:**
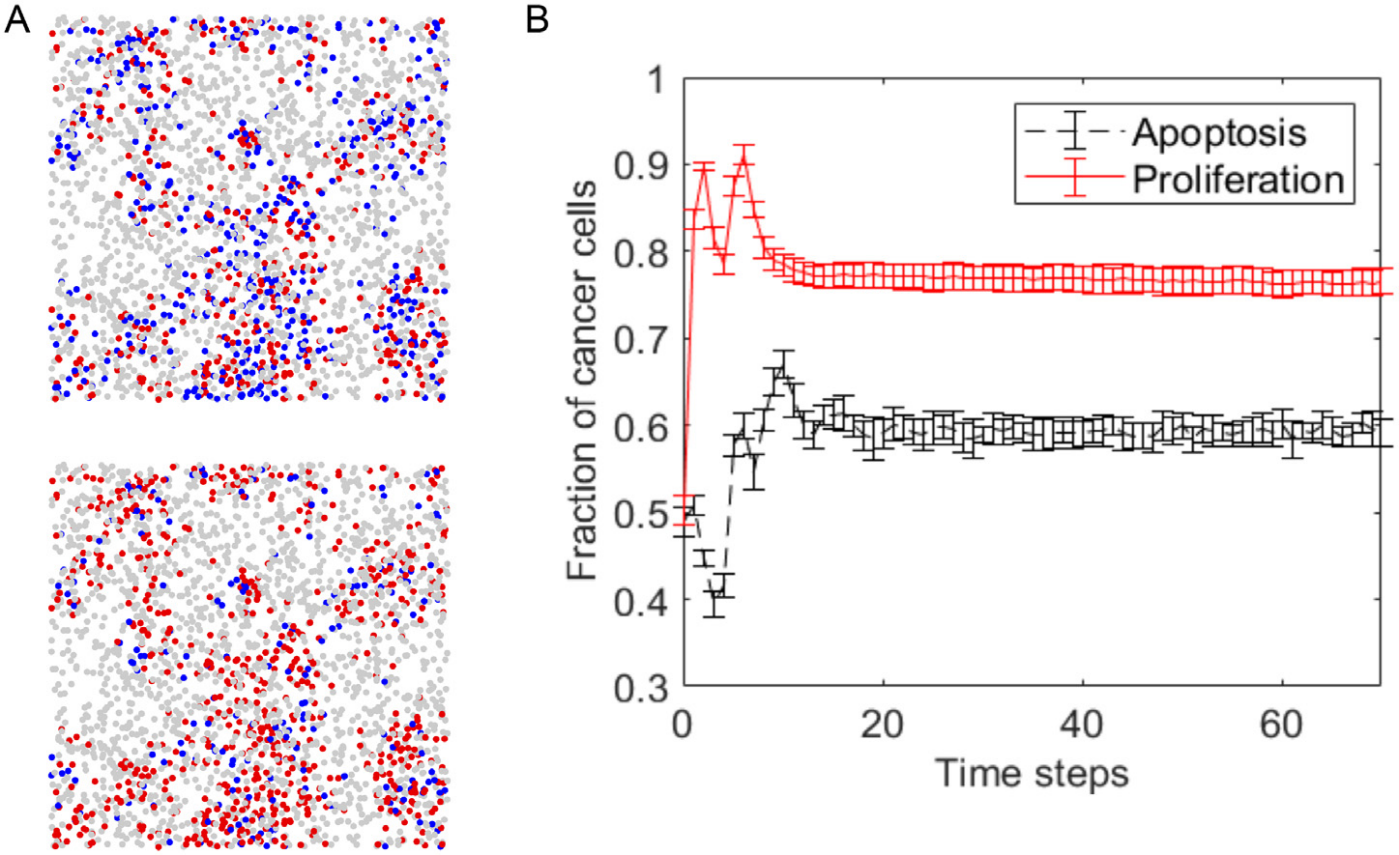
**A**. Top views of 3D spatial configuration of a 2-cell model; stellate cells are in grey while cancer cells are in red for those with proliferation nodes in ON state and blue for those with proliferation nodes in OFF state; for cancer cells we used $s\ = \ 0.07$. The top panel shows the spatial configuration at the beginning of a simulation and the bottom panel shows the configuration after 100 time steps. **B**. The average proportion of cancer cells with active proliferation (red solid line) and apoptosis (black solid line) as a function of time steps. Averages and standard deviations were computed from 10 simulations. More details about simulation parameters can be found in Supplementary Table S2.

To avoid unrealistic high densities of cancer cells, we used a fixed value of $\sigma ,$ such that the density of cells inside the sphere with radius $\sigma $ is limited by a parameter ${{\mathrm{\rho }}_{\mathrm{max}}}$. We set up ${{\mathrm{\rho }}_{\mathrm{max}}} = 8{\mathrm{\rho }}$, so that clusters of cancer cells are more concentrated than stromal cells. Fig. 2A shows an example of the spatial distribution of a system with 2 cell types using a value of $s\ = \ 0.7$, and Supplementary Fig. S1 shows the distribution of cells for different values of *s*, showing that changing *s* changes the distribution of cancer cells from clustered to homogeneous.

## Methods

### Simulations and simulation framework

In a tissue model with *N* cells and *n* genes per cell, there are ${2^{Nn}}$ possible states. Assuming the tissue model reaches a steady state distribution, owing to the ergodic dynamics induced by the perturbation probability *q* [31], the expectation of expression of node *g* in cancer cells is

(5) \begin{equation*}
E\left[ {{f_g}} \right]\ = \ \mathop \sum \nolimits_s {p_s}\ {f_s}\left( g \right), \end{equation*}

where ${p_s}$ is the probability of state $s\ \in \{ 1,2,\ \ldots ,{2^{Nn}}\} $ in the steady state distribution and ${f_s}( g )$ is the fraction of cancer cells with gene *g* in the ON state. Similar equations are used for the expression of other cell types of the system. The distribution of ${p_s}$ depends on model parameter set $\theta $ and the BN for each cell type. Because the number of possible states is very large, we need to approximate the expectation above by performing *M* independent simulations and considering the last *K* steps of each simulation. Thus, the approximation of the expression of gene *g* is

(6) \begin{equation*}
{\hat{f}_g}\left( \theta \right)\ = \frac{1}{{MK}}\ \ \mathop \sum \nolimits_i^M \mathop \sum \nolimits_j^K {f_{{s_{ij}}}}\left( g \right), \end{equation*}

where ${f_{{s_{ij}}}}( g )$ is the fraction of cancer cells with active gene *g* in the state ${s_{ij}}$ of the system in step *j* of simulation *i*. The gene expression profile of cancer cells from the simulations is as follows:

(7) \begin{equation*}
\bar{G}\left( \theta \right)\ = \ \left\{ {{{\hat{f}}_1}\left( \theta \right),\ {{\hat{f}}_2}\left( \theta \right),\ \ldots ,\ {{\hat{f}}_n}\left( \theta \right)} \right\}. \end{equation*}

Simulations of our model were implemented in Biocellion [47], a high-performance computing platform designed for simulation of multicellular systems. At every time step *t* of the simulation, the concentration of signaling molecule *m* is updated by numerically solving equation (3), after which the Boolean states of the cells are updated using the computed concentrations. Figure 2A shows the spatial cellular distribution of a system with 2 cell types (pancreatic cancer cells and stellate cells). Figure 2B shows how the fraction of cancer cells with activated proliferation and apoptosis nodes changes during the simulation; the proportion of cells reaches a steady state after ~25 time steps. The standard deviations and averages of cell fractions were computed from 10 independent simulations using the same parameter values. In remaining sections, if the values are not specified, then results were collected from $M\ = \ 20$ independent simulations of 400 time steps, using the last $K\ = \ 200$ time steps.

**Figure 3:**
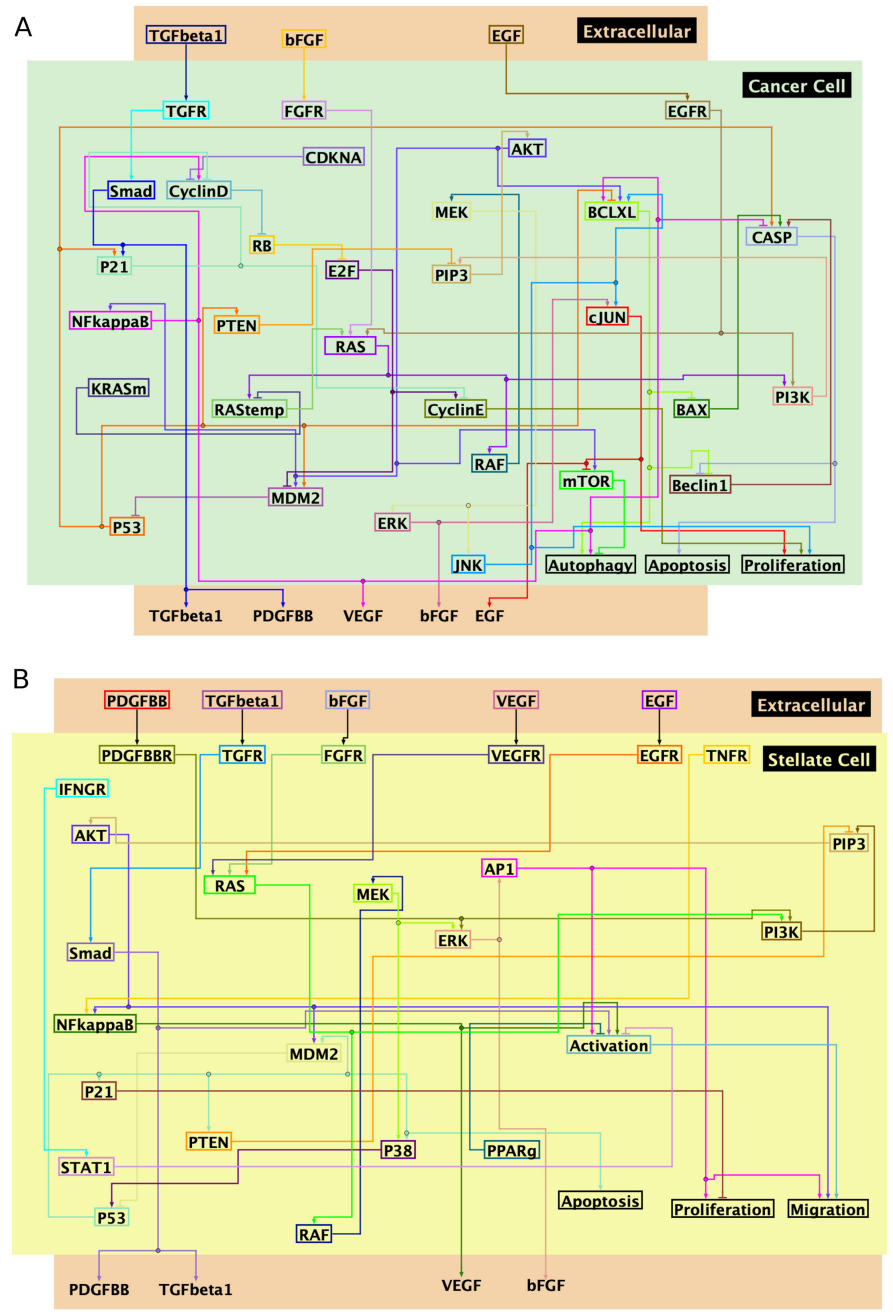
Network of molecular interactions in pancreatic cancer cells (A, green area) and pancreatic stellate cells (B, yellow area). Extracellular cytokines between these 2 cells are in the orange area. Adapted from Wang et al. [48] and illustrated in Biotapestry [59]. The Boolean functions for each gene of the 2 cells are available in Supplementary Tables S3 and S4.

Within the proposed model, the phenotype of the tissue segment is characterized by the average proportion of cells with the corresponding phenotypic node in the ON state (activated); for instance, the cancer proliferation phenotype of the 2 cell types system in Fig. 3 is estimated by averaging the fraction of cancer cells with an activated proliferation node over the last 50 steps of the simulation, which is the time window in which the system is stable (see Fig. 2B).

### Boolean networks

#### Cancer and stellate cells

The BNs of pancreatic cancer cells (PCCs) and pancreatic stellate cells (PSCs) were obtained from Wang et al. [48]. The network includes pathways that were found to be important in PDAC progression, such as the Ras-ERK and PI3K-Akt, TGF$\beta $-SMAD4, and p53 signaling. The network also includes pathways that are important for activation of stellate cells. The cytokines that are used to communicate between these 2 BNs are also available in Wang et al. [48]. Furthermore, we have modified the model to include relevant mutations of PDAC cells including *KRAS,TP53, CDKN2A*, and *SMAD4* mutations, which are present in >30% of the samples from patients with PDAC in The Cancer Genome Atlas (TCGA) [49]. The effect of mutations is modeled by permanently setting nodes to ON or OFF, depending on whether the mutation is functionally activating or inactivating. The mutations are applied to a randomly selected fraction of cancer cells, which in our model is characterized by a parameter ($\alpha $). Moreover, we have removed the HER2-JAK1-STAT pathway because mutations in *HER2* only appear in a small number of TCGA PDAC samples.

#### CD4^+^ T cells

The BN for CD4^+^ cells was obtained from Tieri et al. [11], who model the differentiation of naive CD4^+^ T cells into 4 commonly characterized subtypes: 3 effector cells, type 1 helper (T_H_1) T cell, T_H_2, and T_H_17, and regulatory T cells (Tregs). Each subtype secretes specific sets of cytokines that can influence the behaviors of other cells. The model includes cytokines such as interferon-$\gamma $ (IFN-) secreted by T_H_1 subtypes, interleukin 10 (IL-10) and IL-4 secreted by T_H_2, and IL-17 and IL-6 secreted by T_H_17.

#### Macrophages

We implemented the BN model of macrophage cells developed by Palma et al. [6]. Their BN models macrophage differentiation into 4 commonly characterized subtypes: the immunogenic M1 and 3 immunosuppressive subtypes, M2a, M2b, and M2c. Each of these subtypes is determined by a particular set of expressed genes and cytokines including IL-12 and IL-10. We have extended the model by adding the secretion of TNF-α and IL-6 secreted by M1 and M2b subtypes, and TGF- $\beta $ secreted by M2a and M2c [50, 51].

#### CD8^+^ T cells

We obtained a BN model of CD8^+^ T cells from a recently published article by Bolouri et al. [9], in which the authors study TCR activation and the response of CD8^+^ T cells to cytokines. They developed a BN that models the transition of T cells from naive to acute and exhausted states in response to chronic antigen stimulation. The exhausted CD8^+^ T cell state is characterized by high expression of immune checkpoint molecules and lowered proliferation capacity, cytokine production, and cytotoxic activity compared with effector or memory CD8^+^ T cells [52, 53].

### Parameter calibration

Our tissue model is characterized by a set of parameters listed in Supplementary Table S1; some of these parameters are estimated from data available in TCGA. Specifically, cell fractions were estimated from gene expression data using “cell deconvolution” [54]. The mutation states of patient samples were summarized from a TCGA Pan-Cancer dataset [55], and deconvolved gene expression of cancer cells was generated using the DeMix algorithm [56]; see Estimation of cell fractions section for details concerning cell fraction estimation. Most parameters were calibrated using deconvolved gene expression data. It is worth noting that BNs are static and are not optimized.

The optimization protocol is represented in Supplementary Fig. S2. Our strategy is to optimize the unknown parameter set $\theta $, including secretion rates, activation thresholds, and mutation rates, by minimizing a cost function ${C_p}( \theta )$ defined as the deviation ($\varepsilon \ $ in Supplementary Fig. S2) between the gene expression ${G^{\mathrm{model}}}( \theta )$ of cancer cells in the model and the gene expression of cancer cells obtained from TCGA samples ${G^{\mathrm{TCGA}}}( p )$:

(8) \begin{equation*}
{C_p}\left( \theta \right)\ = \ \varepsilon \left( {{G^{\mathrm{model}}}\left( \theta \right),{G^{\mathrm{TCGA}}}\left( p \right)} \right), \end{equation*}

where *p* is a TCGA sample. We used $\varepsilon ( {x,\ y} )\ = \ 1\ - \ R( {x,\ y} )$ as a cost function ${C_p}( \theta )$, where $R( {x,\ y} )$ is the Spearman correlation coefficient between *x* and *y*. Other alternatives of $\varepsilon ( {{G^{\mathrm{model}}}( \theta ),\ {G^{\mathrm{TCGA}}}( p )} )$ can be tested in the future.

Thus, the optimization problem is to find the set of optimal parameters:

(9) \begin{equation*}
\theta _p^* = \ \mathrm{arg}\ \left[ {\mathrm{min}_{\theta \subset \Theta }\ {C_p}\left( \theta \right)} \right]
\end{equation*}

for each TCGA sample *p*. We used simulated annealing (SA) [57, 58] to minimize ${C_p}( \theta )$. For our particular case, SA consists of the following steps:

1. Initialize ${\theta _i}$ randomly from $\Theta $, the space of parameters listed in Supplementary Table S1.
2. Run *W* steps of the Metropolis algorithm [57] at temperature ${T_k}$. Select a new parameter ${\theta _j}$ from a distribution ${P_{ij}}$ and compute $\Delta \ {C_{ij}} = {C_p}\ ( {{\theta _j}} )\ - {C_p}( {{\theta _i}} )$. If $\Delta {C_{ij}} \le 0$, accept the new parameter set, letting ${\theta _i} = {\theta _j}\ $; otherwise accept the new parameter set ${\theta _j}$ with probability $\mathrm{exp}( { - \Delta {C_{ij}}/{T_k}} )$.
3. Update the temperature, ${T_{k + 1}} = \ 0.8{T_k}$. If ${T_{k + 1}} < \ {T_{\mathrm{min}}}$, then stop the algorithm; otherwise, go to step 2.

We used ${P_{ij}} = \ P({\theta _j}|{\theta _i})\ = \ \mathrm{Gaussian}( {{\theta _i},\ \sigma ( T )} )$, where $\sigma ( T )\ = {\sigma _o}\ T$ and *T* is the temperature. We have used ${T_o} = \ 0.5$, ${\sigma _0} = \ 1.0$, and $W\ = \ 60$ (number of steps in step 2) to generate the optimum parameters for each TCGA sample.

### Estimation of cell fractions

Cellular deconvolution [60] was used to estimate cellular fractions from bulk RNA sequencing (RNA-seq) data. In this work, we used the ADAPTS R package [54] and in particular, the SVMDECON method, which makes estimations based on support vector regression. This method solves the linear model $Y\ = \ AX$, where *Y* is the gene expression of a given sample and *A* is a matrix of gene expression signatures for each cell (in columns). This matrix (*A*) is typically derived from experiments where cells have been physically isolated and then measured in bulk for gene expression. However, the pancreas is composed of cell types not typically found in deconvolution resources. To create a signature matrix that includes pancreatic cells, similar to the methods found in the ADAPTS package [54], we used a pancreatic single-cell RNA sequencing (scRNA-seq) dataset in conjunction with expression signatures for 22 immune cell types (LM22) [61]. The cells found in the scRNA-seq data were previously labeled, providing a set of cells for each type. The median expression for each gene was computed by cell type, giving an expression value per gene per cell type. The goal is to produce a matrix of genes by cell types, where each signature is predictive of that particular cell type, and the matrix overall has a low condition number.

Iterating over cell types using a *t*-test, we selected genes to maximize the difference between one cell type and all others, building up the matrix. As the matrix grows in the number of genes, the condition number is also computed. The number of genes is selected to minimize the condition number. The final cell signature matrix comprises 566 genes for 33 cell types, with 11 cell types specific to the pancreas. The expression values were normalized first independently by data source, then merged and renormalized. The final cell signature matrix is available in Additional File 1.

Non-metastatic pancreatic tumor data from TCGA (PDAC) were used, providing 119 samples. The cancer cell quantities were estimated using ductal cells as a proxy and were found to correlate with tumor purity, the proportion of cancer cells in each sample, which is calculated from publicly available TCGA copy number variation data (Supplementary Fig. S4). A file with estimated cellular fractions for cancer cells, stellate cells, CD4^+^ T cells, macrophages, and CD8^+^ T cells is available in Additional File 2.

### Mutation state of cancer cells from TCGA

For each TCGA sample, we used the MC3 Pan-Cancer somatic mutation table to generate a probability of a cancer cell having a mutated gene [55]. We compute probabilities for *KRAS, TP53, CDKN2A*, and *SMAD4* mutations, which are present in 93%, 73%, 30%, and 32% of the TCGA samples of PDAC, respectively [49]. This was done by taking the number of sequencing reads with a detected mutation and dividing that count by the number of total reads, assuming that the mutated reads come from cancer cells. Thus, for each sample and each gene, we have a probability of gene mutation. A sample-level instantiation is produced by sampling from these Bernoulli distributions. A file with the presence (1) or absence (0) of mutation in *TP53, CDKN2A, SMAD4*, or *KRAS* for each TCGA sample of PDAC is available in Additional File 3.

### Gene expression of cancer cells

Deconvolution of expression into portions of cancer cells and stromal (and immune) tissue compartments was performed using the DeMix software [56]. Expression values had previously been computed and were supplied by the authors of the software. A file with the expression values of cancer genes is available in Additional File 4.

## Results

### Analysis of the interplay of cancer and stellate cells

Previous experimental studies in mice and *in vitro* experiments [62] show that PSCs promote the proliferation of PCCs during the progression of disease. In this section, we use our framework to study the mechanisms that drive the interactions between these two cell types. The BNs and the cytokines that regulate the phenotypic behavior of PSCs and PCCs were adapted from the model published by Wang et al. [48]. Fig. 3 shows the network of interactions between nodes that regulate proliferation, apoptosis, and other important phenotypic behaviors of PSCs and PCCs. We used a standard sensitivity analysis [63] in which random parameter sets are generated using Latin hypercube sampling [63] (LHS) and used for performing simulations. Partial ranked correlation coefficients [63] (PRCC) let us determine the strength of association between model parameters and important properties of tumor samples, such as cancer proliferation and apoptosis states. These properties are characterized in simulations by the average fraction of cells with the corresponding phenotypic node set to ON (see Methods section for details).

**Figure 4:**
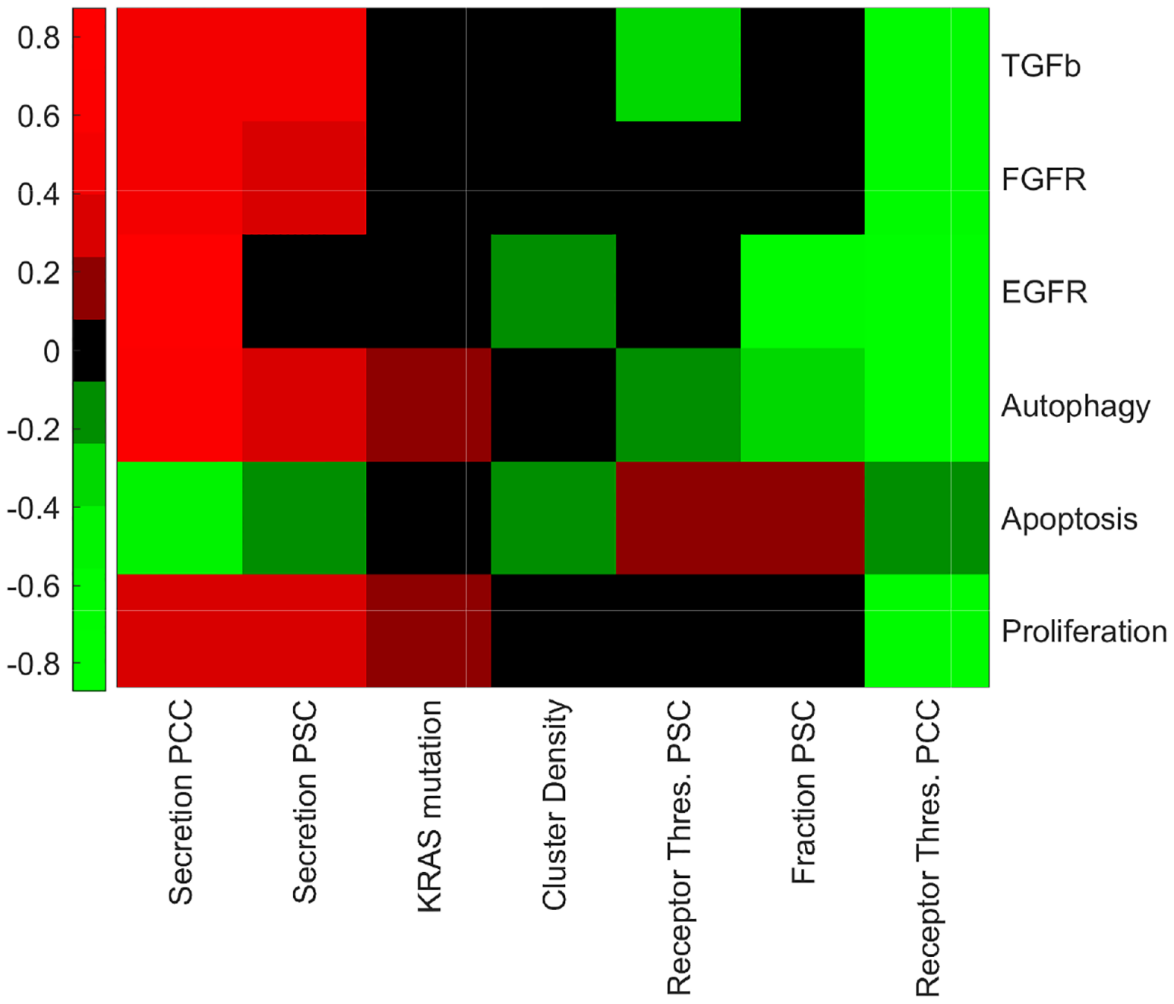
Association of model parameters (columns) with cancer cell phenotypes (rows). Color scale shows partial rank correlation coefficient (PRCC) obtained from simulations of 1,000 random parameters.

The heat map in Fig. 4 shows the PRCC between model parameters and population-level properties. The parameters considered in the sensitivity analysis, parameter ranges, and more details of model simulations are specified in Supplementary Table S2. We generated 1,000 parameter sets using LHS and then performed 100 simulations for each parameter set using the networks in Fig. 3. Each of the 100 simulations started from random initial conditions of the Boolean genes and random cellular positions. The tissue-level properties were averaged over the 100 simulations.

The results suggest that the interaction between cancer and stellate cells can be harmful for cancer cells, inducing apoptosis, or helpful for cancer cells, inducing cancer proliferation. This is evident by the significant positive (red) and negative (green) correlations between model parameters and apoptosis and proliferation, as shown in Fig. 4. Moreover, the results of Fig. 4 show that the secretion rate of cytokines by PCCs and the sensitivity of cytokine receptors in PCCs are most associated with cancer cell behavioral states. Specifically, an increase in secreted cytokines by cancer cells trends with increases (positive correlation) in proliferation and reductions (negative correlation) in apoptosis. The secretion and the sensitivity of receptors of PSC cells also play a role in the phenotypes of cancer cells. In summary, parameters related to cell-cell communications, e.g., secretion rates and activation thresholds, have a significant impact on cancer cell behavior.

Although the correlation between cancer cluster density (a measure of spatial structure) and most phenotypic properties of cancer cells is almost zero, there are several properties that are influenced by spatial organization of cells, namely, the population-level expression of EGFR and the apoptosis state of cancer cells. Thus, the spatial organization of cells, in this case the clustering of cancer cells, is another multicellular property that can potentially influence the interplay between cancer and stellate cells and should be explored in future studies.

A surprising result is the negligible correlation between the fraction of stellate cells and cancer cell proliferation. A positive correlation was expected because it has been previously reported that the stellate cells increase the survival of cancer cells [62, 64, 65]. This may point to the possibility that intercellular communication mechanisms between stellate and cancer cells may play a more dominant role than population numbers alone.

### The role of paracrine and autocrine loops

To explore potential molecular interactions that are key in the relationship between PSC population and PCC proliferation, we have performed a sensitivity analysis after fixing the secretion rates of cancer or stellate sets. These parameters effectively change the strength of intercellular communication and autocrine loops present in both cell types (see Fig. 3). First, simulations with constant and equal secretion rates of cancer and stellate cells were run (R^PSC^ = R^PCC^ = 5). In these simulations all paracrine and autocrine loops are allowed and were given similar weights. The results (Table 1) showed that in this case there are negligible correlations between the population of stellate cells and cancer phenotypes. When the secretion rate of R^PSC^ ≥ R^PCC^, e.g., when the signal from PSC to PCC is stronger, the correlation between the stellate fraction and cancer proliferation increases substantially. This correlation increases to 0.5 when R^PSC^ ≥ R^PCC^ = 2. In summary, these results suggest that asymmetric cytokine-mediated communication between stellate and cancer cells plays a role in the observed positive effect on cancer survival.

**Table 1:** Partial rank correlation coefficient (PRCC) between the fraction of stellate cells (*r*_PSC_) and cancer phenotypes (proliferation, apoptosis, and autophagy).

Secretion rates	PRCC		
	*r* _PSC_ vs proliferation	*r* _PSC_ vs apoptosis	*r* _PSC_ vs autophagy
R^PSC^ = R^PCC^ = 5	0.0555	0.0718	−0.0841
R^PSC^ > R^PCC^ = 5	0.1173	0.1121	−0.1204
R^PSC^ > R^PCC^ = 2	0.4999	−0.2651	0.4406

Simulations were performed with constant values of R^PCC^ and for different ranges of R^PSC^. For the second and third row, 500 random values for R^PSC^ were selected in the range [R^PCC^, 10.0].

According to the model (Fig. 3), cancer cells secrete 4 cytokines, 3 of which (EGF, bFGF, TGF$\beta $) are involved in autocrine loops. To determine the relevance of cancer autocrine loops in the stellate-cancer cells relationship, we assigned different values of secretion rates to the different cytokines secreted by cancer cells, namely, R_EGF_, R_bFGF_, and R_TGF_*_β_*. Table 2 shows that when only the EGF autocrine loop is active (R_EGF_ > R_bFGF_ = R_TGF_*_β_* = 2.0) the population of stellate cells is negligibly correlated with cancer phenotypes. The correlation between stellate cell population and cancer proliferation increases to 0.3 when the bFGF autocrine loop is the only active autocrine loop. The highest (lowest) correlation between stellate cell correlation and cancer proliferation (apoptosis) occurs when the only autocrine loop involved is TGF$\beta $. These results suggest that cancer cell autocrine loops that involve EGFR are key modulators of the interaction between stellate and cancer behaviors. This is consistent with the known role of EGFR in modulating the stroma to support cancer growth [66].

**Table 2:** Partial rank correlation coefficient (PRCC) between fraction of stellate cells (*r*_PSC_) and cancer phenotypes

Secretion rates	PRCC	
	*r* _PSC_ vs proliferation	*r* _PSC_ vs apoptosis
R_EGF_ > R_bFGF_ = R_TGF_*_β_* = 2.0	−0.0474	0.0549
R_bFGF_ > R_EGF_ = R_TGF_*_β_* = 2.0	0.2974	−0.0293
R_TGF_*_β_* > R_EGF_ = R_bFGF_ = 2.0	0.5203	−0.4422

Simulations were performed with different values of secretion rates of EGF, bFGF, and TGFβ secreted by cancer cells.

### Patient-specific models for TCGA samples

**Figure 5:**
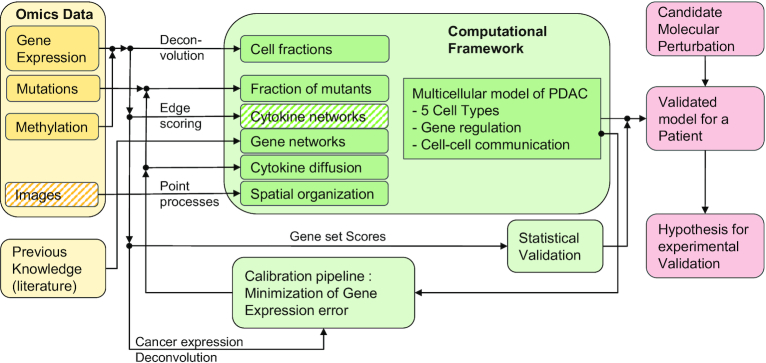
Diagram of the data-driven computational framework to instantiate, calibrate, validate, and explore patient-specific multiscale models of the TME to generate actionable and therapeutically relevant hypotheses.

Owing to inter-patient heterogeneity in terms of somatic alterations or tissue-level properties such as cell fractions, it is important to construct patient-specific models. Toward that end, we have developed methods for the integration of high-throughput molecular data into our modeling framework. Fig. 5 shows a diagram of the analysis workflow, including the used data types from TCGA (yellow), and the methods (arrow labels) for integrating the data and existing knowledge into the process of initialization, parameter calibration, and model validation (green rectangles). Moreover, Table 3 provides additional details of the different data types, the software we used to analyze the data, the outputs, and how those are integrated into the analysis workflow.

**Table 3:** List of molecular data (inputs), methods, and descriptions of how the data are integrated into the modeling framework and the analysis pipeline

Input	Method	Output	Usage
Gene expression from RNA-seq data	ADAPTS [54]	Cellular composition for each sample	Instantiate the proportions of cell types for each model sample
Gene expression from RNA-seq data	DeMix [56]	Gene expression of cancer cells	Used in the parameter optimization process to find errors between simulations and data
Gene expression from RNA-seq data	ssGSEA [67]	Proliferation and apoptosis scores for each sample	Evaluate the optimized models. These scores are compared with phenotypic scores from simulations
Somatic mutation calls from DNA sequence data	CGC [68]	The presence/absence of important mutations found in samples	Used in the calibration process to determine the set of mutation parameters that will be optimized

We built a network of interactions involving intracellular relationships and cytokine-mediated intercellular relations that combine published models of different cell types relevant to PDAC, namely, (epithelial) cancer cells, stellate cells, CD4^+^ T cells, CD8^+^ T cells, and macrophages. The set of BNs for each cell is provided in Supplementary Tables S3–S7. Furthermore, we used cellular deconvolution techniques to estimate cell fractions for each TCGA sample to be used in our model instantiation (see Methods for details of the deconvolution methods). For each sample, DNA sequencing data were used to determine the presence or absence of mutations in *KRAS, TP53, CDKN2A*, or *SMAD4*. If a mutation in one of the 4 genes (*g*) is absent in a sample, then ${\alpha _g} = \ 0$; otherwise ${\alpha _g}$ is calibrated by SA, as described in the Methods section. Although data from histology images can be used to get estimates of the density of cancer clusters [46], these data are not available in TCGA for PDAC samples.

**Figure 6:**
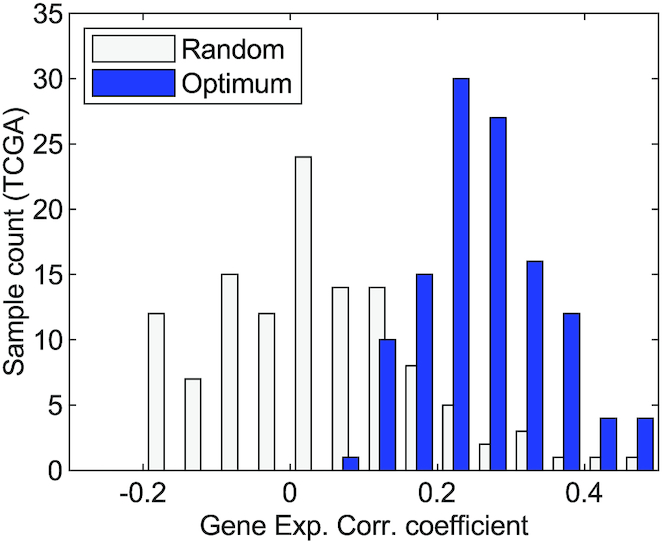
Histogram of correlation (corr.) coefficient between gene expression (exp.) obtained from simulations and those from DeMix expression deconvolution. Blue bars represent the best correlation coefficient obtained testing an ensemble of random parameters. The grey bars represent the correlation coefficient from a random set of parameters.

Model parameters that cannot be directly estimated from TCGA data are listed in Supplementary Table S1. These include rates of cytokine secretion by cancer cells and other cell types, spatial distribution of cancer cells, and receptor activation thresholds. These parameters are calibrated by an optimization process that aims to find an optimum parameter set (${\theta ^*}$) to maximize the Spearman correlation between the deconvolved gene expression of cancer cells obtained from TCGA and simulations of the framework; Supplementary Fig. S2 shows a diagram of the optimization protocol; more details of the optimization process can be found in the Parameter Calibration section above. The optimum parameters (${\theta ^*}$) together with parameters estimated directly from TCGA samples represent personalized models for each TCGA patient sample. Figure 6 shows the histograms of the correlation coefficient of the optimal parameter set compared to random parameters. On average, the correlation coefficient of optimum models over TCGA samples is 0.26, considerably higher than random parameter models, which had an average correlation coefficient of 0.04. Although on average, 0.26 can be improved, there are some samples with correlation coefficient closer to 0.5. By adding more data such as histology images and more detailed models of gene regulation and cell communication, we expect that the accuracy can be further improved. For validating these personalized models, we used gene set scores that can be computed from TCGA gene expression data, using ssGSEA, which is part of the GSVA R package [67]. The Spearman correlation between the fraction of cancer cells in the proliferation state and the proliferation gene set scores from TCGA samples was 0.17, while the correlation between the fraction of cells in the apoptosis state and the apoptosis gene set scores was 0.2.

### Characterizing TCGA subtypes with model parameters

We investigated whether the model parameters, calibrated on TCGA samples, were associated with the previously described subtypes of PDAC. If so, this may reveal an aspect of the model that is more important in particular subtypes, possibly leading to mechanistic hypotheses. Specifically, we measured the difference in parameter values using analysis of variance (ANOVA) followed by Tukey Honest Statistical Difference. The association of model parameters (Supplementary Table S1) was performed using the 4 subtypes discovered by Bailey et al. [69] (squamous, immunogenic, progenitor, and aberrantly differentiated endocrine exocrine [ADEX]) and 2 from Moffitt et al. [70] (basal and classical subtypes).

**Figure 7:**
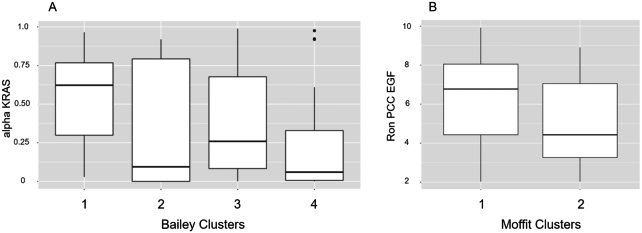
**A**. Fraction of cells with *KRAS*mutation (alpha KRAS) within each subtype defined by Bailey et al. [69], squamous (1), immunogenic (2), progenitor (3), and ADEX (4). **B**. EGF secretion rate of pancreatic cancer cells (Ron PCC EGF) within each subtype defined by Moffitt et al. [70], basal (1) and classical (2). We used 119 samples of PDAC available in TCGA; the barcode identifiers of these samples are available in Additional File 2.

Our results (Fig. 7) showed that among the model parameters, both the probability of *KRAS* mutation (α_KRAS_, ANOVA *P*-value = 0.013) and the secretion rate of EGF from cancer cells ($R_{\mathrm{EGF}}^{\mathrm{PCC}}$, ANOVA *P*-value = 0.038) were associated with Bailey subtypes (Fig. 7A). Also, for Moffitt et al. [70] subtypes (Fig. 7B) associations were found with probability of *TP53* mutation (α_TP53_, *P*-value = 0.01) and EGF secretion rate ($R_{\mathrm{EGF}}^{\mathrm{PCC}}$, *P*-value = 0.009). Probability of *KRAS* mutation was not significantly associated with the Moffitt subtypes (*P*-value = 0.08). It is worth noting that these results and the results of the PCC and PSC interactions (Table 2) reinforce the notion that the EGF autocrine loop plays an important role in PDAC.

## Exploration of Therapeutic Interventions

After the process of parameter calibration and validation, the personalized models can be used to explore the effect of molecular perturbations. A molecular perturbation of a gene is modeled by forcing the state of the gene (a node *k* in the BN of cell type *T*) to 0 to model gene repression, or to 1, to model gene overexpression on a fraction ($\alpha _k^T$) of the cells in the model. By increasing $\alpha _k^T$, we model the strength of the potential therapeutic intervention.

**Figure 8:**
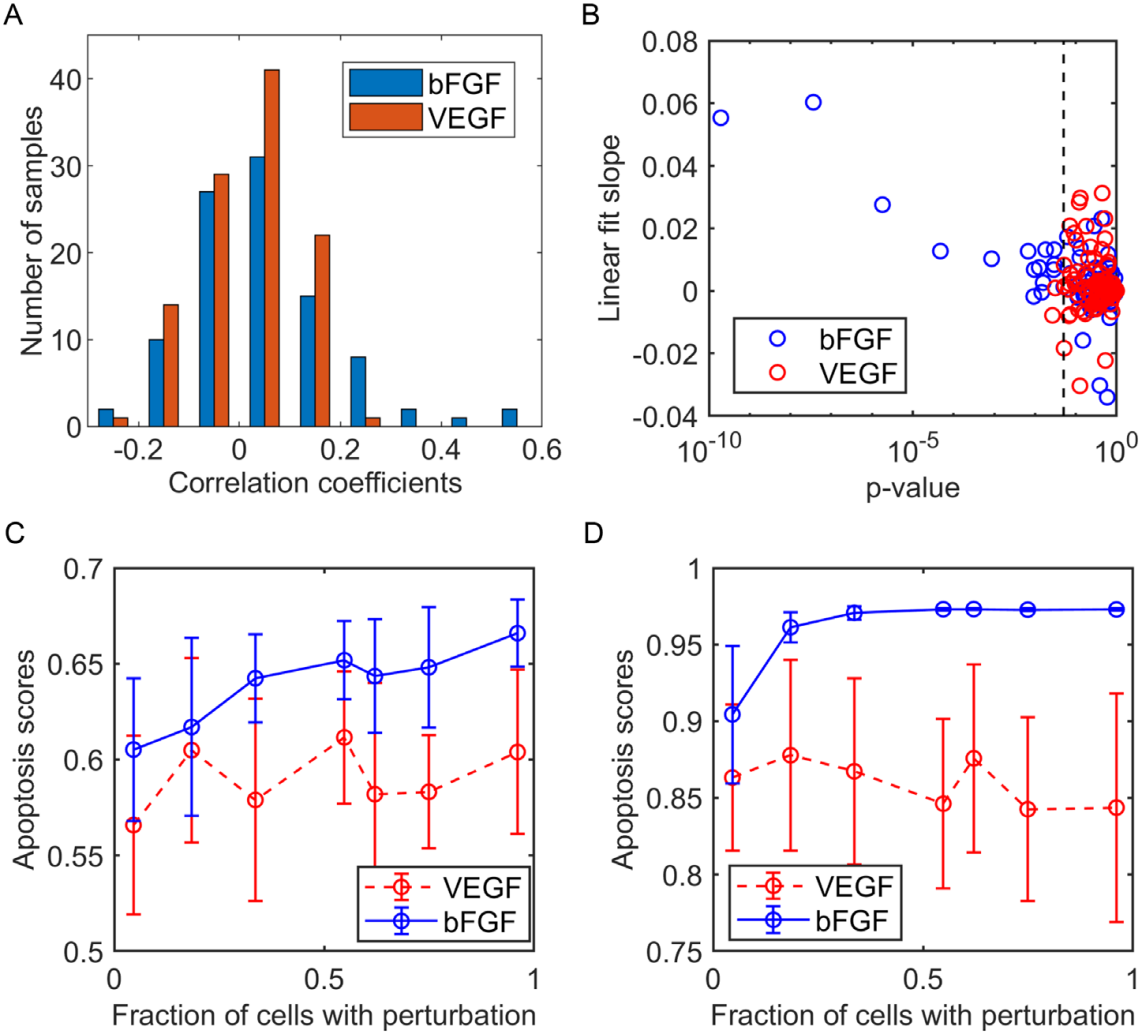
Effects of gene perturbation in stellate cells on apoptosis states in cancer cells. **A**. Distribution of correlation coefficients between apoptosis scores and the percentage of perturbation in bFGF and VEGF in stellate cells, over 119 TCGA samples of PDAC (Additional File 2). **B**. The slope of the linear fit between apoptosis scores and the percentage of perturbed stellate cells vs the *P*-value of the hypothesis that the slope is zero. The blue (red) circles represent samples with a perturbation in bFGF (VEGF) and the dashed vertical line represents a *P*-value = 0.05. **C**. Average apoptosis scores for cancer cells within 1 sample as a function of the percentage of perturbations of bFGF and VEGF for the 2 samples with the largest correlation coefficient; error bars represent standard deviations. Averages and standard deviations were computed from 15 simulations performed with a constant percentage of perturbed cells.

To do this, we performed simulations with different values of $\alpha _k^T$ and computed Spearman correlation coefficients between the values of $\alpha _k^T$ and the apoptosis state of cancer cells to determine whether the perturbation would have an effect. Figure 8A shows the histogram of correlation coefficients between perturbation fractions and apoptosis scores across TCGA samples, focusing on perturbations of bFGF and VEGF nodes in stellate cells. On average perturbing VEGF secretion of stellate cells had a small but negligible effect on cancer apoptosis (average correlation of 0.01). On the other hand, perturbing bFGF had on average a slightly positive impact on cancer apoptosis, with an average of 0.05 across all TCGA samples. It is worth noting that although the estimated effect of bFGF perturbation on apoptosis is small, there are samples with significant positive correlation between perturbation in bFGF in stellate cells and apoptosis of cancer cells. With the null hypothesis that the slope between perturbation fractions and cancer apoptosis is zero, we computed *P*-values and found several samples with *P*-values <0.05, and some examples with *P*-values considerably <0.05 (Fig. 8B).

Fig. 8C and D, comparing 2 TCGA samples, show the apoptosis scores for different fractions of perturbed cells, clearly showing the positive trend of apoptosis induced by perturbation in bGFG, in contrast to the perturbation of VEGF. These results show that TCGA PDAC samples have a heterogeneous response to a perturbation in bFGF cytokine secretion, accounting for the rather weak overall correlation across all samples. Using the model, we can speculate that perturbing the secretion of bFGF by stellate cells could increase cancer cell apoptosis rates for some patients.

## Discussion

It is becoming increasingly evident that interactions between cancer cells and the TME are closely linked to patient outcomes. In this work, we developed a multicellular modeling framework designed to study the molecular interactions between cancer cells and the TME, including stromal and immune cells. This allows model-driven hypotheses to be generated regarding therapeutically relevant PDAC states with potential molecular and cellular drivers, indicating specific potential intervention strategies for further analysis.

The focus of this work is to study how cancer cell states are affected by cell-cell communication within the TME. Only the components of the TME necessary to determine cellular states and intercellular signaling are considered, including gene regulation, spatial distribution of cells, cytokine diffusion, and cell type proportions; other interactions that play a role in tumor growth such as oxygen uptake, mechanical interactions, cell migration, and so forth are not included. Our motivation was to generate multicellular models of cancer with a tractable number of parameters that permits the validation and instantiation of the model with omics data, and efficient parameter exploration. Importantly, many of the model parameters can be directly estimated from omics and imaging data.

Our modeling framework can incorporate intracellular interactions by implementing BNs for each cell type of the TME as well as cell-cell communication by modeling the diffusion of cytokines secreted by the cells in the TME. Moreover, each cell is determined by its spatial position and the state of its corresponding BN. The molecular interactions can be obtained from previous studies that use gene networks to study cell behaviors relevant to the TME. Public datasets of molecular interactions can further facilitate model creation and expansion [71, 72]. Thus, the BNs represent current knowledge about gene regulation of cell behavior. The BNs are not further optimized with experimental data, although BN optimization is a future venue worth exploring.

Given the specific features of the modeling approach, it is worth discussing the implications of the model assumptions. The main assumption is that, with the time scales considered by the model (hours), population changes induced by proliferation, migration, and so forth will not substantially affect the interplay between gene regulation and cell signaling. This implies that the phenotypic estimates generated by model simulations represent instantaneous properties of a sample; extensions need to be added to the model for longer time scales. Another important assumption is that the gene expression data used for parameter calibration is assumed to represent a steady state regime of cellular behavior. This assumption is imposed by the nature of the data used for calibration and validation, which is static; it represents a single time point in the cancer dynamics. The consequences of this assumption can be evaluated using high-throughput data at multiple time points that are currently not available.

Using ensemble simulations over random model parameters, one can investigate the degree of association between potential molecular interactions and important multicellular properties, such as tumor survival or degrees of apoptosis. We have used that strategy on a previously developed 2-cell model of pancreatic cancer. The model consists of interactions between pancreatic cancer cells and stellate cells, connected by inter-cellular interactions mediated by cytokines. Our results show that the EGF-mediated autocrine loop in cancer cells is a potential player in the interactions between stellate and cancer cells. When the EGF autocrine loop is partially repressed, increases in the stellate cell population lead to increases in the proliferation of cancer cells. Moreover, the spatial clustering of cancer cells can affect the expression of important genes, such as the EGF receptor. The last result highlights one of the key components of this modeling framework, namely, the ability to study the influence of spatial cellular properties on the tumor phenotype. A more detailed analysis of the role of the spatial distribution of cells on cancer behavior will require further extension of the model because, for simplicity, we assumed that the stromal cells are uniformly distributed in space and that signal degradation is independent of the spatial organization of the cells.

The molecular scale of the computational framework permits the integration of molecular data from high-throughput omics technologies, such as gene expression and sequencing data. We have developed methods for data integration that allow for the construction of personalized models of PDAC samples. Specifically, gene expression was used to estimate the relative fractions of the cell types included in the models while sequencing data were used to estimate the percentage of cells with mutations in relevant genes. Additionally, tissue histology images could potentially be integrated in the model framework using methods such as those described by Saltz et al. [73]. Images could be used to estimate parameters of spatial properties of tissue samples and improve model instantiation. We have used knowledge of point processes to generate the positions of cancer cells with a user-specified parameter of cancer cell clustering. Recently, it was demonstrated that this parameter can be estimated from histological images [46]. This could lead to complex point processes able to generate more realistic spatial arrangements of cancer or stromal and immune cells.

We built a network of interactions by combining published models of different cell types relevant to PDAC, namely, stellate cells, CD4^+^ T cells, CD8^+^ T cells, and macrophages. Additional BN models can be added to the framework in a straightforward manner. Using this 5–cell type model, we found that *KRAS* mutations and the secretion rate of EGF from cancer cells were associated with Bailey subtypes while *TP53* mutations and EGF secretion rate were associated with the Moffitt subtypes, indicating their potential clinical significance.

In addition to cellular BNs, the modeling framework requires parameters related to cell-cell communication and spatial organization of cells. Some of the parameters can be estimated from molecular data; but for the estimation and calibration of the rest of the parameters (Supplementary Table S1), we proposed an optimization procedure that minimizes the difference in gene expression obtained by simulations and those observed in deconvolved samples from TCGA. The expression of other cell types can also be used in the procedure, but that would require more involved deconvolution techniques or perhaps scRNA-seq. Our optimization procedure is based on SA, but other optimization methods suitable for discrete stochastic dynamics can also be implemented [74]. In particular, recent parameter exploration methods based on machine learning techniques applied to ABM have the potential to generate new and more robust conclusions regarding the influence of cell-cell communication on cancer behavior [75, 76].

The estimation and calibration of the model parameters by using data available in TCGA generates personalized models that are characterized by unique model parameter sets. The generated sample-level models have a mean correlation coefficient of 0.26 between simulated and TCGA-based cancer gene expression, with some samples reaching values of 0.5. We also compute gene set scores of proliferation and apoptosis for each TCGA sample and use these values to assess the personalized models. Overall, the correlation coefficients between gene set scores of apoptosis and proliferation and the fraction of cells in apoptosis and proliferation states obtained from the model simulations are 0.17 and 0.2, respectively. Although these correlation coefficients are relatively low, they are much better than random parameter sets and are expected to improve progressively with the addition of more data, such as imaging data, as well as with more detailed models of gene regulation and cell-cell communication. However, it is worth considering that more detailed models typically require more unknown parameters, which, in the absence of pertinent data, can compromise the model validation process and parameter exploration. Because the proposed model already includes spatial distributions of cells, we anticipate that the integration of images into the proposed model will not substantially increase the model complexity (number of parameters).

The calibrated model parameters can provide additional knowledge about the PDAC samples that cannot readily be obtained by pure data analysis. We have shown that the model parameters are associated with known disease subtypes defined by 2 different studies [69, 70]. This framework also allows researchers to model the effect of potential molecular perturbations, generating hypotheses to be tested using more comprehensive models and analysis, and subsequent experimental set-ups.

## Availability of Source Code and Requirements

Project name: Multicellular BNs

Project home page: https://github.com/boaguilar/multicell_boolean_networks

Code Ocean reproducible capsule: https://doi.org/10.24433/CO.2337238.v1

Operating system(s): Linux

Programming language: C++ and Python

Other requirements: The code requires Biocellion1.2 and Threading Building Blocks library, both free for academic use. We included both dependencies in the repository, so the code is self-contained and ready to be compiled and executed.

License: MIT License

## Availability of Supporting Data and Materials

Snapshots of our code and other supporting data are openly available in the *GigaScience* repository, GigaDB [77].

## Additional Files

Supplementary Figure S1. Examples of generated spatial cellular distributions.

Supplementary Figure S2. Optimization protocol used to calibrate the model parameters.

Supplementary Figure S3. An example of the Simulated Annealing process.

Supplementary Figure S4. Comparison of the estimated cell quantities with tumor purity and leukocyte content.

Supplementary Table S1. List of parameters used in the parameter calibration pipeline.

Supplementary Table S2. List of parameters used in the Sensitivity Analysis.

Supplementary Table S3. Boolean network of pancreatic cancer cells.

Supplementary Table S4. Boolean network of pancreatic stellate cells.

Supplementary Table S5. Boolean network of CD4^+^T cells.

Supplementary Table S6. Boolean network of macrophages.

Supplementary Table S7. Boolean network of CD8^+^T cells.

Additional File 1. Signature matrix including pancreatic cells for the estimation of cell fractions.

Additional File 2. Barcodes and cellular fractions for each TCGA sample of PDAC.

Additional File 3. Presence (1) or absence (0) of mutation in *TP53, CDKN2A, SMAD4*, or *KRAS* for each TCGA sample of PDAC.

Additional File 4. Gene expression of cancer cells obtained by DeMix [56].

giaa075_GIGA-D-19-00272_Original_Submission

giaa075_GIGA-D-19-00272_Revision_1

giaa075_GIGA-D-19-00272_Revision_2

giaa075_Response_to_Reviewer_Comments_Original_Submission

giaa075_Response_to_Reviewer_Comments_Revision_1

giaa075_Reviewer_1_Report_Original_Submission Thomas Gorochowski -- 8/30/2019 Reviewed

giaa075_Reviewer_2_Report_Original_Submission Francesca Buffa -- 10/15/2019 Reviewed

giaa075_Reviewer_2_Report_Revision_1 Francesca Buffa -- 4/14/2020 Reviewed

giaa075_Reviewer_3_Report_Original_Submission Paul Macklin, Ph.D. -- 10/15/2019 Reviewed

giaa075_Reviewer_3_Report_Revision_1 Paul Macklin, Ph.D. -- 2/17/2020 Reviewed

giaa075_Supplemental_Files

## Abbreviations

ABM: agent-based modeling; ADAPTS: Automated Deconvolution Augmentation of Profiles for Tissue Specific Cells; ADEX: aberrantly differentiated endocrine exocrine; ANOVA: analysis of variance; BN: Boolean networks; CGC: Cancer Genomics Cloud; ERK: extracellular-signal-regulated kinase; HER2: human epidermal growth factor receptor 2; LHS: Latin hypercube sampling; MaBoSS: Markovian Boolean Stochastic Simulator; PDAC: pancreatic ductal adenocarcinoma; PCC: pancreatic cancer cell; PI3K: phosphoinositide 3-kinase; PRCC: partial ranked correlation coefficients; PSC: pancreatic stellate cell; RNA-seq: RNA sequencing; SA: simulated annealing; scRNA-seq: single-cell RNA sequencing; ssGSEA: Single-Sample Gene Set Enrichment Analysis; TAM: tumor-associated macrophages; TCGA: The Cancer Genome Atlas; TCR: T cell receptor; TGF: transforming growth factor; TME: tumor microenvironment.

## Competing Interests

B.A., D.L.G., and I.S. declare no competing interests.

D.J.R., M.M., S.A.D., A.D., M.T., D.B., and A.V.R.: Bristol-Myers Squibb: Employment, Equity Ownership.

A.D.: Twinstrand Biosciences: Equity Ownership; Bristol-Myers Squibb: Employment, Equity Ownership.

R.H.: Adaptive Biotechnologies: Membership on an entity's Board of Directors or advisory committees; Fraizer Healthcare Partners: Consultancy; NanoString Technologies: Membership on an entity's Board of Directors or advisory committees; Silverback Therapeutics: Membership on an entity's Board of Directors; Celgene: Former Employment, Equity Ownership.

## Funding

This study was funded by Celgene Corporation through a Sponsored Research Agreement between Celgene Corporation and the Institute for Systems Biology.

## Authors' Contributions

I.S. and A.V.R. conceived the study; B.A., D.L.G., A.V.R., and I.S. designed the research; R.H., A.D., M.T., and D.B. provided feedback on the research design; B.A., D.L.G., D.J.R., A.D., R.H., A.V.R, and I.S.: conceptualization; B.A. and D.L.G.: investigation and formal analysis; B.A., D.L.G., D.J.R., M.M., S.A.D., A.V.R., and I.S.: methodology design; M.T., D.B., R.H., A.V.R., and I.S.: project administration and supervision; B.A. and D.L.G. wrote the manuscript; I.S. and A.V.R. revised the manuscript. All authors read and approved the final draft.
