# Supplementary material for: A generalizable data-driven multicellular model of pancreatic ductal adenocarcinoma
Source: Gigascience. 2020 Jul 22;9(7):giaa075. doi: 10.1093/gigascience/giaa075 (PMC7374045; doi:10.1093/gigascience/giaa075)
Supplement: giaa075_GIGA-D-19-00272_Original_Submission [file giaa075_giga-d-19-00272_original_submission.pdf]

GigaScience

A generalizable data-driven multicellular model of pancreatic ductal adenocarcinoma

--Manuscript Draft--

Manuscript Number:	GIGA-D-19-00272
Full Title:	A generalizable data-driven multicellular model of pancreatic ductal adenocarcinoma
Article Type:	Research
Funding Information:	
Abstract:	Background<br><br>Mechanistic models, when combined with pertinent data, can improve our knowledge regarding important molecular and cellular mechanisms found in cancer. These models make the prediction of tissue level response to drug treatment possible, which can lead to new therapies and improved patient outcomes. We present a data-driven multiscale modeling framework to study molecular interactions between cancer, stromal, and immune cells found in the tumor microenvironment. We also develop methods to use molecular data available in The Cancer Genome Atlas (TCGA) to generate patient-specific models of cancer.<br><br>Results<br><br>By combining published models of different cells relevant to pancreatic ductal adenocarcinoma (PDAC), we built an agent-based model of the multicellular pancreatic tumor microenvironment, formally describing cell type-specific molecular interactions and cytokine mediated cell-cell communications. We used an ensemble-based modeling approach to systematically explore how variations in the tumor microenvironment affect the state of cancer cells. The results suggest that the autocrine loop involving EGF signaling is a key interaction modulator between ductal cancer and stellate cells. EGF is also found to be associated with previously described subtypes of PDAC. Moreover, the model allows a systematic exploration of the effects of possible therapeutic perturbations; our simulations suggest that reducing bFGF secretion by stellate cells will have, on average, a positive impact on cancer cell apoptosis.<br><br>Conclusions<br><br>The developed framework allows model-driven hypotheses to be generated regarding therapeutically relevant PDAC states with potential molecular and cellular drivers indicating specific intervention strategies.
Corresponding Author:	Boris Aguilar<br>Institute for Systems Biology<br>Seattle, WA UNITED STATES
Corresponding Author Secondary Information:	
Corresponding Author's Institution:	Institute for Systems Biology
Corresponding Author's Secondary Institution:	
First Author:	Boris Aguilar
First Author Secondary Information:	
Order of Authors:	Boris Aguilar
	David L Gibbs
	David L Reiss
	Mark McConnell

Powered by Editorial Manager® and ProduXion Manager® from Aries Systems Corporation

	Samuel A Danziger
	Andrew Dervan
	Matthew Trotter
	Douglas Bassett
	Rob Hershberg
	Alexander V Ratushny
	Ilya Shmulevich
**Order of Authors Secondary Information:**	
**Additional Information:**	
**Question**	**Response**
Are you submitting this manuscript to a special series or article collection?	No
**Experimental design and statistics**<br><br>Full details of the experimental design and statistical methods used should be given in the Methods section, as detailed in our Minimum Standards Reporting Checklist. Information essential to interpreting the data presented should be made available in the figure legends.<br><br>Have you included all the information requested in your manuscript?	Yes
**Resources**<br><br>A description of all resources used, including antibodies, cell lines, animals and software tools, with enough information to allow them to be uniquely identified, should be included in the Methods section. Authors are strongly encouraged to cite Research Resource Identifiers (RRIDs) for antibodies, model organisms and tools, where possible.<br><br>Have you included the information requested as detailed in our Minimum Standards Reporting Checklist?	Yes
**Availability of data and materials**	Yes

All datasets and code on which the conclusions of the paper rely must be either included in your submission or deposited in publicly available repositories (where available and ethically appropriate), referencing such data using a unique identifier in the references and in the “Availability of Data and Materials” section of your manuscript.<br><br>Have you have met the above requirement as detailed in our Minimum Standards Reporting Checklist?	

Powered by Editorial Manager® and ProduXion Manager® from Aries Systems Corporation

Manuscript
Click here to download Manuscript PDAC Manuscript Gigascience.docx

Title: A generalizable data-driven multicellular model of pancreatic ductal adenocarcinoma

Authors:

Boris Aguilar[1] boris.aguilar@systemsbiology.org

David L Gibbs[1], david.gibbs@systemsbiology.org

David L Reiss[2], dreiss@celgene.com

Mark McConnell[2], mmcconnell@celgene.com

Samuel A Danziger[2], sdanziger@celgene.com

Andrew Dervan[2], adervan@celgene.com

Matthew Trotter[3], mtrotter@celgene.com

Douglas Bassett[2], dbassett@celgene.com

Rob Hershberg[2], rhershberg@celgene.com

Alexander V Ratushny[2*], aratushny@celgene.com

Ilya Shmulevich[1*], ilya.shmulevich@systemsbiology.org

* corresponding authors

Affiliations:

[1] Institute for Systems Biology, Seattle WA, 98109, USA

[2] Celgene, Seattle WA, 98109, USA

[3] Celgene Institute for Translational Research Europe (CITRE), Seville 41092, Spain

Abstract

Background

Mechanistic models, when combined with pertinent data, can improve our knowledge regarding important molecular and cellular mechanisms found in cancer. These models make the prediction of tissue level response to drug treatment possible, which can lead to new therapies and improved patient outcomes. We present a data-driven multiscale modeling framework to study molecular interactions between cancer, stromal, and immune cells found in the tumor microenvironment. We also develop methods to use molecular data available in The Cancer Genome Atlas (TCGA) to generate patient-specific models of cancer.

Results

By combining published models of different cells relevant to pancreatic ductal adenocarcinoma (PDAC), we built an agent-based model of the multicellular pancreatic tumor microenvironment, formally describing cell type-specific molecular interactions and cytokine mediated cell-cell communications. We used an ensemble-based modeling approach to systematically explore how variations in the tumor microenvironment affect the state of cancer cells. The results suggest that the autocrine loop involving EGF signaling is a key interaction modulator between ductal cancer and stellate cells. EGF is also found to be associated with previously described subtypes of PDAC. Moreover, the model allows a systematic exploration of the effects of possible therapeutic perturbations; our simulations suggest that reducing bFGF secretion by stellate cells will have, on average, a positive impact on cancer cell apoptosis.

Conclusions

The developed framework allows model-driven hypotheses to be generated regarding therapeutically relevant PDAC states with potential molecular and cellular drivers indicating specific intervention strategies.

Keywords

Cancer modeling, data-driven model, pancreatic ductal adenocarcinoma, multicellular model

Introduction

Pancreatic ductal adenocarcinoma (PDAC), the most common form of pancreatic cancer, is the fourth leading cause of cancer associated death in the United States and is predicted to be the second in 2030 [1]. With a 5-year survival rate of only 3%, it has a very poor prognosis. Across all types of cancer, it is becoming increasingly clear that interactions within the tumor microenvironment (TME) have a strong effect on tumor growth. This is particularly relevant for PDAC research where previous studies have revealed high heterogeneity and complexity in the tumor microenvironment, where a mixture of interacting immune cells, stromal tissue and cancer cells are resident. However, much remains to be known regarding how differences in the TME affect the behavior of cancer cells. For instance, there is a debate concerning whether stroma-cancer interactions are associated with progression of pancreatic cancer or, rather, provide protective measures [2]. Thus, to make progress in the treatment of PDAC, new strategies must be developed to improve our understanding of the effects of the tumor microenvironment on cancer states and progression.

In silico models are frequently used in systems biology for the discovery of general principles and novel hypotheses [3–5]. Moreover, it is eventually possible that when combined with relevant data, *in silico* models will make predictions with sufficient accuracy for therapeutic treatment. Despite their potential, concrete examples of predictive models of cancer progression are scarce. One reason is that most models have focused on single cell dynamics, ignoring the

interactions between cancer cells and their local microenvironment. Indeed, there have been a number of models that were used to study gene regulation at the single cell scale, such as macrophage differentiation [6–8], T cell exhaustion [9], differentiation and plasticity of T helper cells [10,11], cell cycle [12–14], and regulation of key genes in different tumor types [15].

Although not as numerous as single cell models, multicellular models have progressively been developed to study different aspects of cancer biology, including tumor immunosurveillance [16–19], hypoxia [20,21], angiogenesis [22,23], and epithelial-mesenchymal transition [24,25], among others; we refer the reader to Metzcar et al. [26] for a recent and comprehensive review. Typically, these models are based on phenomenological rules to model cell behavior and therefore use limited data to calibrate their parameters. Although multicellular models are being increasingly used in cancer biology, there remains a need for a modeling framework that is capable of integrating different multiscale properties of the TME, such as molecular and cellular heterogeneity and non-uniform spatial distributions of cells, with the capacity to leverage diverse -omics datasets for model building, calibration and validation, allowing researchers to explore novel molecular therapies *in silico* [3,27–29].

In this work, we developed a modeling framework designed to study the interaction between cancer cells and their microenvironment. Figure 1 shows a schematic of the modeling framework and the methods of patient-specific model generation. The framework is a combination of two well established approaches: Boolean Networks [30] (BNs) and Agent Based Modeling (ABM), used at the molecular and cellular levels, respectively. The cancer signaling and regulatory networks are modeled with BNs, while ABM is used to simulate intercellular networks consisting of different cell types and intercellular signaling molecules. This vertical (“multiscale”) integration, using ABM and BNs, enables the exploration of therapeutic interventions on the molecular level for inducing transitions of the tumor into less harmful states, while utilizing currently available high-throughput molecular data.

We built a network of cell type-specific intracellular interactions and cytokine mediated intercellular communications, by combining published models of different cell types relevant to PDAC, namely, the ductal cancer cells, stellate cells, $CD4^+$ T cells, $CD8^+$ T cells, and macrophages. Through computational simulations, using an ensemble modeling approach, we have used this framework to study how the tumor microenvironment, characterized by a set of cytokines, stromal cells, and somatically heterogeneous cancer cells, affects the viability of cancer cells.

Modeling framework

In this section, we describe our approach to model a block of cancerous tissue with a mixture of cancer, stromal, and immune cells randomly located inside a 3D rectangular simulation domain (Figure 1A). Each cell possesses a Boolean network that determines its cellular phenotype (functional state), such as proliferation or apoptosis, and the possible secretion of key cytokines. Membrane receptors, sensitive to cytokine concentrations, are also part of the Boolean networks. The following subsections present a detailed description of each component of our modeling approach:

Cells as Boolean networks Signal transduction and gene regulation in a given cell is modeled with Boolean networks (BNs), a well-known modeling approach used to study several cellular processes important in cancer [31,32]. The BN of a cell i is defined on a set of n binary-valued variables $X_i = \{x_1^i, \dots, x_n^i\}$, where a node $x_j^i \in \{0, 1\}$ represents the expression of a gene, a cellular behavior, or secretion of a cytokine to the TME. We divided the nodes into two groups: signal receptors and regulatory nodes. Receptor nodes sense the presence of signaling molecules in the local TME, with their updating rules being specified in the next subsection. Regulatory nodes are updated in discrete time steps by conventional logic rules. Specifically, the regulatory node j

of a cell i at $t+1$(next time step) is determined by the values of the nodes ("genes") , $x^i_{j_1}, x^i_{j_2}, \ldots, x^i_{j_{k_{j,i}}}$at time t by means of the Boolean function, $F^i_j\colon \{0,1\}^{k_{j,i}} \to \{0,1\}$. There are $k_{j,i}$ nodes assigned as inputs to regulatory node x^i_j, thereby determining the wiring of the BN. Thus, the Boolean value of a regulatory node x^i_j is given by

$$x^i_j(t+1) \;=\; F^i_j(\, x^i_{j_1}(t), \ldots, x^i_{j_{k_{j,i}}}(t)\,) \;\ldots (1).$$

It is worth noting that regulatory genes are updated using the states of nodes of the same cell, whereas membrane receptors are updated by the TME, that is, by the presence of cytokines.

Additionally, to model stochastic dynamics, following the convention used in random Boolean networks [30,33], we introduce a perturbation probability q and a random perturbation vector, $\gamma = [\,\gamma_1,\, \gamma_2, \ldots, \gamma_n\,]$, where $\gamma_j \in \{0,1\}$and $P\{\gamma_j = 1\} = q$, such that:

$$X_i(t+1) \;= X_i(t) \;\oplus\; \gamma, \text{ with probability } (1-(1-q)^n)$$

$$X_i(t+1) \;=\; [\,F^i_1, F^i_2, \ldots, F^i_n\,], \text{ otherwise,}$$

where $\oplus$ indicates the modulo-2 sum. The fact that any state transition has a nonzero probability under this perturbation model implies that the dynamics of the network are described by an ergodic Markov chain with a (unique) steady-state distribution.

The state, X_i, of every cell at time step t, is determined by the state of its Boolean nodes. Some of the regulatory nodes are associated with important cellular behaviors, such as proliferation, apoptosis, or migration. Moreover, some of the regulatory nodes are associated with the secretion of cytokines in such a way that a state of 0 or 1 of these nodes corresponds to low or high rates of secretion, respectively.

Cell-cell communication via secretion and sensing of cytokines We include communication between cells by modeling the secretion and sensing of diffusible cytokines. A cell i releases

cytokine m with a secretion rate of $\eta_m^i(x_{S_m}^i)$ molecules per time step, which depends on the Boolean state of its designated signal node $x_{S_m}^i$ (S_m is the label of one of the regulatory nodes of cell i). We assume that $\eta_m^i(0) = 1$ and $\eta_m^i(1) = R_m^i$, $R_m^i > 1$, to account for basal and active expression, respectively. We make this assumption with no loss of generality since it is equivalent to normalizing active expression by the lower basal expression. The concentration, C, of cytokine m changes in space and time according to a diffusion degradation equation. For cells randomly scattered in a rectangular lattice, the concentration of cytokine m in a voxel v is approximated by

$$\partial C_m^v/\partial t = D \vartriangle C_m^v - \gamma C_m^v + V^{-1} \sum_{i \in v} \eta_m^i(x_{S_m}^i) \quad \ldots (2)$$

for each voxel v of the lattice containing the set of cells. D is the diffusion coefficient, γ is the constant degradation rate, and V is the volume of the voxels. Assuming that diffusion is much faster than gene regulation, we use the steady state of the diffusion equation above,

$$0 = D \vartriangle C_m^v - \gamma C_m^v + V^{-1} \sum_{i \in v} \eta_m^i(x_{S_m}^i) \quad \ldots (3)$$

and use a numerical solver for calculating C_m^v in simulations. An important component of the steady state solution is the effective interaction distance, λ, where $\lambda = \sqrt{D/\gamma}$ [34,35].

To sense cytokine m, cell i checks the local concentration of the signal, i.e., the concentration at its containing voxel. If the local concentration of m is above a threshold value, K_m^i , then the signal receptor is activated, otherwise it is deactivated. Formally, the state of the receptor node x_R^i of cell i, located in voxel v, follows the equations:

$$x_R^i(t+1) = 1, if\ C_m^v(t) > K_m^i, \quad \ldots (4)$$

$$x_R^i(t+1) = 0, \text{ otherwise,}$$

where C_m^v is the concentration of m in voxel v that contains cell i. We will vary λ and K_m^i to change communication within the tissue.

Note that while our model assumes diffusion-based cell-cell communication, the effective interaction distance can be shortened such that the system behaves as if signaling were contact-mediated, the latter effectively being a special case of the former.

Tissue Architecture We have constructed a model tissue as a 3D point process of cells, each represented by a Boolean network and a spatial point in a rectangular block of size L. We assume a fixed density of cells, ρ, and divide cell types into cancer and stromal. The density of cancer cells is $\rho_C = r_C\, \rho$ where r_C is the fraction of cancer cells in the tissue sample. The density of stromal cells is $\rho_S = (1 - r_C)\, \rho$. The positions of cancer cells were generated by a Thomas process [36] in which points are scattered around cluster centers according to a 3D Gaussian distribution with zero mean and covariance matrix $\sigma^2 I$, where I is the 3×3 identity matrix. The cluster centers are generated by a simple Poisson process with intensity ρ_{cc}. Stromal cells are generated by a Void process [37] in which points are removed if they are within a distance R_{ex} from a cluster center. The same cluster centers were used for the cancer and stromal cells. The cluster centers are generated using a Poisson process with density $\rho_{cc} = s\rho_C$, where s is a parameter that determines the clustering of cancer cells.

In order to avoid unrealistic high densities of cancer cells, we have used a fixed value of σ, such that the density of cells inside the sphere with radius σ is limited by a parameter ρ_{max}. We have set up $\rho_{max} = 8\rho$, so that clusters of cancer cells are more concentrated than stromal cells. Figure S1 (supporting material) shows the distribution of cells for different values of s, showing that changing s changes the distribution of cancer cells from clustered to homogeneous.

Methods

Simulations and simulation framework

In a tissue model with N cells and n genes per cell, there are $2^{N\,n}$ possible states. Assuming the tissue model reaches a steady state distribution, owing to the ergodic dynamics induced by the perturbation probability q [30], the average expression of node g in cancer cells is:

$$E[f_g] = \sum_{s} p_s\, f_s(g) \quad \ldots(5)$$

where p_s is the probability of state $s \in \{1,2,\ldots,2^{N\,n}\}$ in the steady state distribution and $f_s(g)$ is the fraction of cancer cells with gene g in the ON state. Similar equations are used for the expression of other cell types of the system. The distribution of p_s depends on model parameter set θ and the Boolean network for each cell type. Since the number of possible states is astronomical, we need to approximate the expectation above by performing M independent simulations and considering the last K steps of each simulation. Thus, the approximation of the average expression of gene g is:

$$\hat{f}_g(\theta) = \frac{1}{MK} \sum_{i}^{M} \sum_{j}^{K} f_{s_{ij}}(g) \quad \ldots(6)$$

where $f_{s_{ij}}(g)$ is the fraction of cancer cells with active gene g in the state s_{ij} of the system in step j of experiment i. The gene expression profile of cancer cells from the simulations is:

$$\bar{G}(\theta) = \{\hat{f}_1(\theta), \hat{f}_2(\theta), \ldots, \hat{f}_n(\theta)\} \ \ldots(7)$$

Simulations of our model were implemented in *Biocellion* [38], a high-performance computing platform designed for simulation of multicellular systems. At every time step t of the simulation, the concentration of signaling molecule m is updated by numerically solving the

equation 3, after which the Boolean states of the cells are updated using the computed concentrations.

Boolean networks

Cancer and stellate cells The Boolean networks of pancreatic cancer cells (PCC) and pancreatic stellate cells (PSC) were obtaining from Wang et al. [39]. The network includes pathways that were found to be important in PDAC progression, such as the RAS-ERK and PI3K-AKT, TGF□-SMAD4 and p53 signaling. The network also includes pathways that are important for activation of stellate cells. The cytokines that are used to communicate between these two Boolean networks are also available in Wang et al. [39]. Further, we have modified the model in order to include relevant mutations of PDAC cells including KRAS, TP53, CDKN2A, and SMAD4 mutations, which are present in more than 30% of the PDAC patient samples in The Cancer Genome Atlas (TCGA) [40]. The effect of mutations is modeled by permanently setting nodes to ON or OFF. The mutations are applied to a randomly selected fraction of cancer cells, which in our model is characterized by a parameter (α). Moreover, we have removed the HER2-JAK1-STAT pathway as mutations in HER2 only appear in a small number of TCGA PDAC samples.

$CD4^+$ T cells The Boolean network for $CD4^+$ cells was obtained from Tieri et al. [11] which model the differentiation of naive $CD4^+$ T cells into four commonly characterized subtypes: three effector cells, Th1, Th2, and Th17, and protective regulatory cells, Tregs. Each subtype secretes specific sets of cytokines that can influence the behaviors of other cells. The model includes cytokines such as IFN□ secreted by Th1 subtypes, IL-10 and IL-4 secreted by Th2, and IL-17 and IL-6 secreted by Th17.

Macrophages We implemented the Boolean network model of macrophage cells developed by Palma et al. [6] Their BN models macrophage differentiation into 4 commonly characterized subtypes: the immunogenic M1 and three immunosuppressive subtypes, M2a, M2b, and M2c. Each of these subtypes is determined by a particular set of expressed genes and cytokines including IL-12 and IL-10. We have extended the model adding the secretion of TNF and IL-6 secreted by M1 and M2b subtypes, and TGF□ secreted by M2a and M2c [41,42].

CD8⁺T cells We obtained a BN model of CD8⁺ T cells from a recently published paper by Bolouri et al. [9], in which the authors study TCR activation and the response of CD8⁺ T cells to cytokines. They developed a BN that models the transition of T cells from naïve to acute and exhausted states in response to chronic antigen stimulation. The exhausted CD8⁺ T cell state is characterized by high expression of immune checkpoint molecules, and lowered proliferation capacity, cytokine production, and cytotoxic activity compared to effector or memory CD8⁺ T cells [43,44].

Parameter Calibration

Our tissue model is characterized by a set of parameters listed in Table S2; some of these parameters, such as cellular fractions, are estimated from data available in TCGA. However, the majority of parameters need to be calibrated from the available gene expression data. Our strategy for parameter calibration is to optimize the unknown parameter set θ by minimizing a cost function $C_p(\theta)$ defined as the deviation between the gene expression $\underline{G}(\theta)$ of cancer cells in the model and the gene expression of cancer cells obtained from TCGA samples $G^{tcga}(p)$ using the deconvolution algorithm DeMix [45]:

$$C_p(\theta) = d(\bar{G}(\theta), G^{tcga}(p)) \ldots (8)$$

where p is a TCGA sample. We used $d(x,y) = 1 - R(x,y)$ as a cost function $C_p(\theta)$, where $R(x,y)$ is the Spearman correlation coefficient between x and y. Other alternatives of $d(\bar{G}(\theta), G^{tcga}(p))$ can be tested in the future.

Thus, the optimization problem is to find the set of optimal parameters:

$$\theta_p^* = arg\ [\ min_{\theta \subset \Theta}\ C_p(\theta)\ ]\ \ldots(9)$$

for each TCGA sample p. We have used simulated annealing (SA) [46,47] to minimize $C_p(\theta)$. For our particular case, SA consists of the following steps:

1. Initialize the $\theta_i$randomly from Θ, the space of parameters listed in Table 1.
2. Run W steps of the Metropolis algorithm [46] at Temperature T_k. Select a new parameter θ_j from a distribution P_{ij} and compute $\Delta C_{ij} = C_p(\theta_j) - C_p(\theta_i)$. If $\Delta C_{ij} \leq 0$, accept the new parameter set, letting $\theta_i = \theta_j$, otherwise accept the new parameter set θ_j with probability $exp(-\Delta C_{ij}/T_k\ )$.
3. Change the temperature, $T_{k+1} = 0.8T_k$. If $T_{k+1} < T_{min}$then stop the algorithm, otherwise, go to step 2.

We used $P_{ij} = P(\theta_j|\theta_i\ ) = Gaussian(\ \theta_i, \sigma(T)\ )$ where $\sigma(T) = \sigma_o T$and T is the temperature. We have used $T_o = 0.5$, $\sigma_0 = 1.0$ and $W = 60$ (number of steps in step 2) to generate the optimum parameters for each TCGA sample.

Estimation of cell fractions

Cellular deconvolution [48] was used to estimate cellular fractions from bulk RNA-seq data. Deconvolution of expression data depends on having gene expression signatures that are derived from cell sorting experiments. However, the pancreas is composed of cell types not typically found in deconvolution resources. To create a signature matrix that includes pancreatic

cells, we used a pancreatic single cell RNA-seq (scRNA-seq) data set in conjunction with expression signatures for 22 immune cells (LM22) [49]. The cells measured in the scRNA-seq data were previously labeled, providing a set of cells for each type. The median expression for each gene was computed by cell type, giving an expression value per gene per cell type. The goal is to produce a matrix of genes by cell types, where each signature is predictive of that particular cell type, and the matrix overall has a low condition number.

Iterating over cell types using a *t*-test, we selected genes so as to maximize the difference between one cell type and all others, building up the matrix. As the matrix grows in the number of genes, the condition number is also computed. The number of genes are selected to minimize the condition number. The final cell signature matrix was 566 genes for 33 cell types, with 11 cell types specific to the pancreas. The expression values were normalized first independently by data source, then merged and renormalized. The final cell signature matrix is included in the supplementary data.

TCGA PAAD data was used, with metastatic samples removed, leaving 119 samples. Using support vector regression in ADAPTS [49], we deconvolved the TCGA samples, generating cell quantities for each sample. The cancer cell quantities were estimated using ductal cells as a proxy, and were found to correlate with tumor purity, the proportion of cancer cells in a given sample, which is calculated from publicly available TCGA copy number variation data (Figure S3 in Supporting material).

Mutation state of cancer cells from TCGA

For each previously selected TCGA sample, we used the MC3 PanCancer somatic mutation table to generate a probability of a cancer cell having a mutated cell [50]. We compute probabilities for KRAS, TP53, CDKN2A, and SMAD4 mutations, which are present in more than 30% of the TCGA PAAD samples [40] . This was done by taking the number of sequencing reads

with a detected mutation and dividing that count by the number of total reads, assuming that the mutated reads come from cancer cells. Thus, for each sample and each gene, we have a probability of gene mutation. A sample level instantiation is produced by sampling from these Bernoulli distributions.

Gene expression of cancer cells

Deconvolution of expression into portions of cancer cells and stromal (and immune) tissue compartments was performed using the DeMix software [45]. Expression values had previously been computed and were supplied by the authors of the software.

Results

Analysis of the interplay of cancer and stellate cells

Previous experimental studies in mice and *in vitro* experiments [51] show that pancreatic stellate cells (PSC) promote the proliferation of pancreatic cancer cells (PCC) during the progression of disease. In this section, we use our framework to study the mechanisms that drive the interactions between these two cell types. The Boolean networks and the cytokines that regulate the phenotypic behavior of PSC and PCC were adapted from the model proposed by Wang et al. [39]. Figure 2 shows the network of interactions between nodes that regulate the proliferation, apoptosis, and other important phenotypic behaviors of PSCs and PCCs. We used a standard sensitivity analysis [52] in which random parameter sets are generated using Latin Hypercube Sampling [52] (LHS) and used for performing simulations. Partial Ranked Correlation

Coefficients [52] (PRCC) let us determine the strength of association between model parameters and important properties of tumor samples, such as cancer proliferation and apoptosis states.

The heatmap in Figure 3 shows the PRCC between model parameters and population level properties. The parameters considered in the SA and their ranges are specified in Table S1 of the supporting material. We generated 1000 parameter sets using LHS and then performed 100 simulations for each parameter set using the network in Figure 2. Each of the 100 simulations started from random initial conditions of the Boolean genes and random cellular positions. The tissue level properties were averaged over the 100 simulations.

The results show that the secretion rates of cytokines by PCCs and the sensitivity of cytokine receptors in PCCs are most associated with cancer cell behavioral states. Specifically, an increase in secreted cytokines by cancer cells trends with increases (positive correlation) in proliferation and reductions (negative correlation) in apoptosis. The secretion and the sensitivity of receptors of PSC cells also play a role in the phenotypes of cancer cells. In summary, parameters related to cell-cell communications, e.g. secretion rates and activation thresholds, have a significant impact on cancer cell behavior.

Although the correlation between cancer cluster density (a measure of spatial structure) and most phenotypic properties of cancer cells is almost zero, there are several properties that are influenced by spatial organization of cells, namely the population-level expression of EGFR and the Apoptosis state of cancer cells. Thus, spatial organization is another multicellular property that can potentially influence cancer cells and should be explored in future studies.

A surprising result is the negligible correlation between the fraction of stellate cells and cancer cell proliferation. A positive correlation was expected as it has been previously reported that the stellate cells increase the survival of cancer cells [51,54,55].

The role of autocrine loops

To explore potential molecular interactions that are key in the relationship between PSC population and PCC proliferation, we have performed a sensitivity analysis after fixing the secretion rates of cancer or stellate sets. These parameters effectively change the strength of intercellular communication and autocrine loops present in both cell types (see Figure 2). First, experiments with constant and equal secretion rates of cancer and stellate cells were run ($R^{PSC} = R^{PCC} = 5$). In these experiments all paracrine and autocrine loops are allowed and were given similar weights. The results (Table 1) showed that in this case there are negligible correlations between the population of stellate cells and cancer phenotypes. When the secretion rate of R^{PSC} is greater or equal to R^{PCC}, e.g., when signal from PSC to PCC is stronger, the correlation between the stellate fraction and the cancer proliferation increases substantially. This correlation increases to 0.5 when R^{PSC} is greater or equal to R^{PCC}=2. In summary, these results show that the previously observed positive effect on cancer survival exerted by stellate cells requires an asymmetric cytokine mediated communication between these two cells.

	**PRCC**		
**Secretion rates**	$\boldsymbol{r_{PSC}}$ **vs Proliferation**	$\boldsymbol{r_{PSC}}$ **vs Apoptosis**	$\boldsymbol{r_{PSC}}$ **vs Autophagy**
$R^{PSC} = R^{PCC} = 5$	0.0555	0.0718	-0.0841
$R^{PSC} > R^{PCC} = 5$	0.1173	0.1121	-0.1204
$R^{PSC} > R^{PCC} = 2$	0.4999	-0.2651	0.4406

Table 1: Partial Rank Correlation Coefficient (PRCC) between the fraction of stellate cells ($\boldsymbol{r_{PSC}}$) and cancer phenotypes (Proliferation, Apoptosis, and Autophagy). Simulations were performed with constant values of R^{PCC} and for different ranges of R^{PSC}. For the second and third row R^{PSC}, 500 random values were selected from the range [R^{PCC} ,10.0].

According to the model (Figure 2), cancer cells secrete 4 cytokines, 3 of which (EGF, bFGF, TGF□) are involved in autocrine loops. To determine the relevance of cancer autocrine loops in the stellate-cancer cells relationship, we assigned different values of secretion rates to the different cytokines secreted by cancer cells, namely R_{EGF}, R_{bFGF}, and $R_{TGF□}$. Table 2 shows that when only the EGF autocrine loop is active (R_{EGF} > R_{bFGF} = $R_{TGF□}$ = 2.0) the population of stellate cells is negligibly correlated with cancer phenotypes. The correlation between stellate cell population and cancer proliferation increases to 0.3 when the bFGF autocrine loop is the only active autocrine loop. The highest (lowest) correlation between stellate cell correlation and cancer Proliferation (Apoptosis) occurs when the only autocrine loop involved is TGF□. These results suggest that cancer cell autocrine loops that involve EGFR are key regulators of the interaction between stellate and cancer behaviors. This is consistent with the known role of EGFR in increased PSC activation and migration and in modulating the stroma to support cancer growth [56].

	**PRCC**	
**Secretion Rates**	$\boldsymbol{r_{PSC}}$ **vs Proliferation**	$\boldsymbol{r_{PSC}}$ **vs Apoptosis**
R_{EGF} > R_{bFGF} = $R_{TGF□}$ = 2.0	-0.0474	0.0549
R_{bFGF} > R_{EGF} = $R_{TGF□}$ = 2.0	0.2974	-0.0293
$R_{TGF□}$ > R_{EGF} = R_{bFGF} = 2.0	0.5203	-0.4422

Table 2: Partial Rank Correlation Coefficient (PRCC) between fraction of stellate cells ($\boldsymbol{r_{PSC}}$) and cancer phenotypes. Simulations were performed with different values of secretion rates of EGF, bFGF, and TGF□ secreted by cancer cells.

Patient-specific models for TCGA samples

Owing to inter-patient heterogeneity in terms of somatic alterations or tissue-level properties such as cell fractions, it is important to construct patient-specific models. Toward that end, we have developed methods for the integration of high-throughput molecular data into our modeling framework. Specifically, we used PDAC TCGA data to instantiate simulation model parameters. Figure 1B shows a diagram of the computational framework, which includes methods for integrating TCGA data and existing knowledge into the process of initialization and parameter estimation. We built a network of interactions involving intracellular relationships and cytokine mediated intercellular relations that combine published models of different cell types relevant to PDAC, namely, (epithelial) cancer cells, stellate cells, $CD4^+$ T cells, $CD8^+$ T cells, and macrophages. The set of Boolean networks for each cell is provided in the Supporting Material (Tables S3-S7 in the supporting material). Further, we used cellular deconvolution techniques to estimate cell fractions for each TCGA sample to be used in our model instantiation (see Methods for details of the deconvolution methods). For each sample, DNA sequencing data was used to determine the presence or absence of mutations in KRAS, TP53, CDKN2A, or SMAD4. These mutations are present in more than 30% of the TCGA PDAC patients [40] . If a mutation in one of the four genes (g) is absent in a sample then $\alpha_g = 0$, otherwise α_g is calibrated by an optimization procedure. Although data from histology images can be used to get estimates of the density of cancer clusters [37], these data are not available in TCGA for PDAC samples.

Model parameters that cannot be directly estimated from TCGA data are listed in Table S2 of the supporting material. These include rates of cytokine secretion by cancer cells and other cell types, spatial distribution of cancer cells, and receptor activation thresholds. These parameters are calibrated by an optimization process that aims to find an optimum parameter set (θ^*) to maximize

the Spearman correlation between the deconvolved gene expression of cancer cells obtained from TCGA and simulations of the framework. As described in Methods, we used simulated annealing for this optimization. The optimum parameters (θ^*) together with parameters estimated directly from TCGA samples represent personalized models for each TCGA patient sample. Figure 4 shows the histograms of the correlation coefficient of the optimal parameter set compared to random parameters. On average, the correlation coefficient of optimum models over TCGA samples is 0.26, considerably higher than random parameter models, which had an average correlation coefficient of 0.04. Although on average, 0.26 can be improved, there are some samples with correlation coefficient closer to 0.5. By adding more data such as histology images and more detailed models of gene regulation and cell communication, we believe the accuracy can be further improved. For validating these personalized models, we used gene set scores that can be computed from TCGA gene expression, using ssGSEA, part of the GSVA R package [57]. The correlation between the fraction of cancer cells in the Proliferation state and the proliferation gene set scores from TCGA samples was 0.17, while the correlation between the fraction of cells in the Apoptosis state and the apoptosis gene set scores was 0.2.

Characterizing TCGA subtypes with model parameters

We investigated whether the model parameters, calibrated on TCGA samples, were associated with the previously described subtypes of PDAC. If so, this may reveal a particular aspect of the model that is more important in particular subtypes, possibly leading to mechanistic hypotheses. Specifically, we measured the difference in parameter values using ANOVA followed by Tukey's Honest Statistical Difference. The association of model parameters (Table S2 of the supporting material) was performed using the four subtypes discovered by Bailey et al. [58] (Squamous, Immunogenic, Progenitor, and ADEX) and two from Moffitt et al. [59](Basal and Classical subtypes).

Our results (Figure 5) showed that among the model parameters, both the probability of KRAS mutation (alpha_KRAS, ANOVA p-value=0.013) and the secretion rate of EGF from cancer cells (ANOVA p-value=0.038) were associated with Bailey subtypes (Figure 5A). Also, for Moffitt et al. [59] subtypes (Figure 5B) associations were found with probability of TP53 mutation (p-value=0.01), and EGF secretion rate (p-value=0.009). Probability of KRAS mutation was not significant (p-value=0.08). It is worth noting that these results and the results of the PCC and PSC interactions (Table 2) reinforce the notion that the EGF autocrine loop plays an important role in PDAC.

Exploration of therapeutic interventions

After the process of parameter calibration and validation, the personalized models can be used to explore the effect of molecular perturbations. A molecular perturbation of a gene is modeled by forcing the state of the gene (a node k in the Boolean network on cell type T) to 0 to model gene repression, or to 1, to model gene overexpression on a fraction (α_k^T) of the cells in the model. By increasing α_k^T, we model the strength of the potential therapeutic intervention.

To do this, we performed simulations with different values of α_k^T and computed Spearman correlation coefficients between the values of α_k^T and the Apoptosis state of cancer cells to determine if the perturbation would have an effect. Figure 6A shows the histogram of correlation coefficients between perturbation fractions and apoptosis scores across TCGA samples, focusing on bFGF and VEGF nodes in stellate cells. On average perturbing VEGF secretion of stellate cells had a small negative impact on cancer apoptosis (average correlation of 0.03). On the other hand, perturbing bFGF had on average a positive impact on cancer apoptosis, with an average of 0.15 across TCGA samples. Figure 6B shows the correlation plots between apoptosis scores for the different values of fractions of perturbed cells of a single TCGA sample, clearly showing

the positive trend in contrast to the perturbation of TGF□. These results show that TCGA PDAC samples have a heterogeneous response to a perturbation in bFGF cytokine secretion. Moreover, perturbing the secretion of bFGF by stellate cells can, potentially, increase cancer cell apoptosis rates.

Discussion

It is becoming increasingly evident that interactions between cancer cells and the tumor microenvironment (TME) are closely linked to patient outcomes. In this work, we developed a multicellular modeling framework designed to study the molecular interaction between cancer cells and the TME, including stromal and immune cells. This allows model-driven hypotheses to be generated regarding therapeutically relevant PDAC states with potential molecular and cellular drivers indicating specific intervention strategies.

Our modeling framework can incorporate intracellular interactions by implementing Boolean networks for each cell type of the TME as well as cell-cell communication by modeling the diffusion of cytokines secreted by the cells in the TME. Moreover, each cell is determined by its spatial position and the state of its corresponding Boolean networks.

The molecular interactions can be obtained from previous studies that use gene networks to study cell behaviors indicative of the TME. Public datasets of molecular interactions can further facilitate model creation and expansion [60,61]. Using ensemble simulations over random model parameters, one can investigate the degree of association between potential molecular interactions and important multicellular properties, such as tumor survival or degrees of apoptosis. We have used that strategy on a previously developed two cell model of pancreatic cancer. The model consists of interactions between pancreatic cancer cells and stellate cells, connected by inter-cellular interactions mediated by cytokines. Our results show that the EGF mediated autocrine loop in cancer cells is a key player in the interactions between stellate and cancer cells.

When the EGF autocrine is loop is partially repressed, then increases in the stellate cell population lead to increases in the proliferation of cancer cells. Moreover, the spatial clustering of cancer cells can affect the expression of important gene expression, such as the expression of the EGF receptor. The last result highlights one of the key components of this modeling framework, namely, the ability to study the influence of spatial cellular properties.

The molecular scale of the computational framework permits the integration of molecular data from high-throughput omics technologies, such as gene expression and sequencing data. We have developed methods for data integration that allow for the construction of personalized models of PDAC samples. Specifically, gene expression was used to estimate the relative fractions of the cell types included in the models while sequencing data was used to estimate the percentage of cells with mutations in relevant genes. Additionally, tissue histology images could potentially be integrated in the model framework. Images could be used to determine spatial properties of tissue samples and improve model instantiation. We have used knowledge of point processes to generate the positions of cancer cells with a user specified parameter of cancer cell clustering. Recently, it was demonstrated that this parameter can be estimated from histological images [37]. This could lead to complex point processes able to generate more realistic spatial arrangements of cancer or stromal and immune cells.

We built a network of interactions by combining published models of different cell types relevant to PDAC, namely, stellate cells, $CD4^+$ T cells, $CD8^+$ T cells, and macrophages. Additional Boolean network models can be added to the framework.

In addition to cellular BNs, the modeling framework requires parameters related to cell-cell communication and spatial organization of cells. Some of the parameters can be estimated from molecular data; but for the estimation and calibration of the rest of the parameters (Table S2 of the supporting material), we proposed an optimization procedure that minimizes the difference in gene expression obtained by simulations and those observed in samples, here from the TCGA. Using the expression of other cell types can also be used in the procedure, but it will require more

involved deconvolution techniques or perhaps single cell RNA-seq. Our optimization procedure is based on simulated annealing; but other optimization methods suitable for discrete stochastic dynamics can also be implemented.

The estimation and calibration of the model parameters by using data available in TCGA generates personalized models that are characterized by unique model parameter sets. The generated sample-level models have an average correlation coefficient of 0.26 between simulated and TCGA-based cancer gene expression, with some samples reaching values of 0.5. We also compute gene set scores of proliferation and apoptosis for each TCGA sample and use these values to assess the personalized models. Overall the correlation coefficient between gene set scores of apoptosis and proliferation and the fraction of cells in Apoptosis and Proliferation states obtained from the model simulations are 0.17 and 0.2, respectively. Although these correlation coefficients are relatively low, they are much better compared to random parameter sets, and are expected to improve progressively with the addition of more data, such as imaging data, and more detailed models of gene regulation and cell-cell communication.

The calibrated model parameters can provide additional knowledge about the PDAC samples that cannot readily be obtained by pure data analysis. We have shown the model parameters are associated with known disease subtypes. Our results showed that secretion of EGF by cancer cells is associated with subtypes from Moffitt et al. [59] and subtypes defined by Bailey et al. [58] This framework also allows researchers to model the effect of potential molecular perturbations, which can be tested in subsequent experimental setups.

Additional files

Additional file 1: Supporting material of the manuscript.

Additional file 2: Signature matrix including pancreatic cells for the estimation of cell fractions.

Abbreviations

ABM: Agent based modeling; BN: Boolean networks; LHS: Latin hypercube sampling; PDAC: Pancreatic ductal adenocarcinoma; PAAD: Pancreatic adenocarcinoma; PCC: Pancreatic cancer cells; PRCC: Partial ranked correlation coefficients; PSC: Pancreatic stellate cells; SA: Simulated annealing; TAM: Tumor associated macrophages; TCGA: The cancer genome Atlas; TME: Tumor microenvironment.

Competing of interest

B.A., D.L.G., and I.S. declare no competing interests.

D.L.R., M.M., S.A.D., A.D., D.B. and A.R.: Celgene Corporation: Employment, Equity Ownership.

M.T.: Celgene Research SL (Spain), part of Celgene Corporation: Employment, Equity Ownership.

A.D.: Twinstrand Biosciences: Equity Ownership; Celgene Corporation: Employment, Equity Ownership.

R.H.: Adaptive Biotechnologies: Membership on an entity's Board of Directors or advisory committees; Fraizer Healthcare Partners: Consultancy; NanoString Technologies: Membership on an entity's Board of Directors or advisory committees; Silverback Therapeutics: Membership on an entity's Board of Directors; Celgene Corporation: Employment, Equity Ownership.

Funding

This study was funded by Celgene Corporation through a Sponsored Research Agreement between Celgene Corporation and the Institute for Systems Biology.

Author’s contributions

I.S. and A.V.R. conceived the study; B.A., D.L.G., A.V.R., and I.S. designed the research; R.H., A.D., M.T., and D.B. provided feedback on the research design; B.A., D.L.G., D.L.R., A.D., R.H., A.V.R, and I.S.: conceptualization; B.A. and D.L.G.: investigation and formal analysis; B.A., D.L.G., D.L.R., M.M., S.A.D., A.V.R., and I.S.: methodology design; M.T., D.B., R.H., A.V.R., and I.S.: project administration and supervision; B.A. and D.L.G. wrote the manuscript; I.S. and A.V.R. revised the manuscript. All authors read and approved the final draft.

Acknowledgements

The authors thank Wenyi Wang for kindly providing gene expression of cancer cells in TCGA samples obtained by DeMix. The authors thank Alessandro Palma for kindly providing the Boolean network of macrophages. We also thank William Longabaugh for creating the BioTapestry network used in Figure 2.

List of Figures

Figure 1. **A.** Cartoon representation of the multiscale model including multiple cell types and cytokines of the TME. **B.** Diagram of the data-driven computational framework to instantiate, calibrate, validate and explore patient-specific multiscale models of the TME to generate actionable and therapeutically relevant hypotheses.

Figure 2. Network of molecular interactions in pancreatic cancer cells (Green area) and pancreatic stellate cells (Yellow area). Extracellular cytokines between these two cells are located in the orange area. Adapted from Wang et al. [39] and illustrated in Biotapestry [53].

Figure 3. Association of Model Parameters (Columns) with cancer cell phenotypes (Rows). Color scale shows Partial Rank Correlation Coefficient (PRCC) obtained from simulations of 1000 random parameters.

Figure 4. Histogram of correlation coefficient between gene expression obtained from simulations and those from DeMix expression deconvolution. Blue Bars are the best correlation coefficient obtained testing an ensemble of random parameters. The Grey bars are the correlation coefficient from a random set of parameters.

Figure 5. A. PCC secretion rate of EGF parameter values within each subtype defined by Bailey et al. [58] squamous (1), immunogenic (2), progenitor (3), and ADEX (4). **B.** PCC secretion rate of EGF parameter values within each subtype defined by Moffitt et al. [59], basal (1) and classical (2).

Figure 6. Effect of gene perturbation in stellate cells on Apoptosis states in cancer cells. **A.** Distribution of correlation coefficients between apoptosis scores and the percentage of perturbation in bFGF (blue) and VEGF (Red) in stellate cells, over TCGA samples. The vertical dashed lines represent averages over samples. **B.** Apoptosis scores for cancer cells within one sample as a function of the percentage of perturbations of bFGF (blue) and TGF□ (green) within one sample. The dashed lines represent linear regression of the data.

References:

1. Rahib L, Smith BD, Aizenberg R, Rosenzweig AB, Fleshman JM, Matrisian LM. Projecting

cancer incidence and deaths to 2030: the unexpected burden of thyroid, liver, and pancreas cancers in the United States. Cancer Res. 2014;74: 2913–2921.

2. Gore J, Korc M. Pancreatic Cancer Stroma: Friend or Foe? Cancer Cell. 2014. pp. 711–712. doi:10.1016/j.ccr.2014.05.026

3. Baker RE, Peña J-M, Jayamohan J, Jérusalem A. Mechanistic models versus machine learning, a fight worth fighting for the biological community? Biol Lett. 2018;14. doi:10.1098/rsbl.2017.0660

4. Huang S. The Tension Between Big Data and Theory in the “Omics” Era of Biomedical Research. Perspect Biol Med. 2018;61: 472–488.

5. Mast FD, Ratushny AV, Aitchison JD. Systems cell biology. The Journal of Cell Biology. 2014. pp. 695–706. doi:10.1083/jcb.201405027

6. Palma A, Jarrah AS, Tieri P, Cesareni G, Castiglione F. Gene Regulatory Network Modeling of Macrophage Differentiation Corroborates the Continuum Hypothesis of Polarization States. Front Physiol. 2018;9: 1659.

7. Rex J, Albrecht U, Ehlting C, Thomas M, Zanger UM, Sawodny O, et al. Model-Based Characterization of Inflammatory Gene Expression Patterns of Activated Macrophages. PLoS Comput Biol. 2016;12: e1005018.

8. Castiglione F, Tieri P, Palma A, Jarrah AS. Statistical ensemble of gene regulatory networks of macrophage differentiation. BMC Bioinformatics. 2016;17: 506.

9. Bolouri H, Young M, Beilke J, Johnson R, Fox B, Huang L, et al. Integrative network modeling reveals mechanisms underlying T cell exhaustion. doi:10.1101/582312

10. Mendoza L, Xenarios I. A method for the generation of standardized qualitative dynamical

systems of regulatory networks. Theor Biol Med Model. 2006;3: 13.

11. Tieri P, Prana V, Colombo T, Santoni D, Castiglione F. Multi-scale Simulation of T Helper Lymphocyte Differentiation. Advances in Bioinformatics and Computational Biology. 2014. pp. 123–134. doi:10.1007/978-3-319-12418-6_16

12. Li F, Long T, Lu Y, Ouyang Q, Tang C. The yeast cell-cycle network is robustly designed. Proc Natl Acad Sci U S A. 2004;101: 4781–4786.

13. Tyson JJ. Modeling the cell division cycle: cdc2 and cyclin interactions. Proc Natl Acad Sci U S A. 1991;88: 7328–7332.

14. Novák B, Tyson JJ. A model for restriction point control of the mammalian cell cycle. J Theor Biol. 2004;230: 563–579.

15. Choi M, Shi J, Jung SH, Chen X, Cho K-H. Attractor landscape analysis reveals feedback loops in the p53 network that control the cellular response to DNA damage. Sci Signal. 2012;5: ra83.

16. Kather JN, Poleszczuk J, Suarez-Carmona M, Krisam J, Charoentong P, Valous NA, et al. Modeling of Immunotherapy and Stroma-Targeting Therapies in Human Colorectal Cancer. Cancer Res. 2017;77: 6442–6452.

17. Ghaffarizadeh A, Heiland R, Friedman SH, Mumenthaler SM, Macklin P. PhysiCell: An open source physics-based cell simulator for 3-D multicellular systems. PLoS Comput Biol. 2018;14: e1005991.

18. Gong C, Milberg O, Wang B, Vicini P, Narwal R, Roskos L, et al. A computational multiscale agent-based model for simulating spatio-temporal tumour immune response to PD1 and PDL1 inhibition. J R Soc Interface. 2017;14. doi:10.1098/rsif.2017.0320

19. Wells DK, Chuang Y, Knapp LM, Brockmann D, Kath WL, Leonard JN. Spatial and Functional Heterogeneities Shape Collective Behavior of Tumor-Immune Networks [Internet]. PLOS Computational Biology. 2015. p. e1004181. doi:10.1371/journal.pcbi.1004181

20. Gatenby RA, Smallbone K, Maini PK, Rose F, Averill J, Nagle RB, et al. Cellular adaptations to hypoxia and acidosis during somatic evolution of breast cancer. British Journal of Cancer. 2007. pp. 646–653. doi:10.1038/sj.bjc.6603922

21. Smallbone K, Gatenby RA, Gillies RJ, Maini PK, Gavaghan DJ. Metabolic changes during carcinogenesis: Potential impact on invasiveness. Journal of Theoretical Biology. 2007. pp. 703–713. doi:10.1016/j.jtbi.2006.09.010

22. Spill F, Guerrero P, Alarcon T, Maini PK, Byrne HM. Mesoscopic and continuum modelling of angiogenesis. J Math Biol. 2015;70: 485–532.

23. McDougall SR, Anderson ARA, Chaplain MAJ. Mathematical modelling of dynamic adaptive tumour-induced angiogenesis: clinical implications and therapeutic targeting strategies. J Theor Biol. 2006;241: 564–589.

24. Reher D, Klink B, Deutsch A, Voss-Böhme A. Cell adhesion heterogeneity reinforces tumour cell dissemination: novel insights from a mathematical model. Biol Direct. 2017;12: 18.

25. Rejniak KA, Wang SE, Bryce NS, Chang H, Parvin B, Jourquin J, et al. Linking changes in epithelial morphogenesis to cancer mutations using computational modeling. PLoS Comput Biol. 2010;6. doi:10.1371/journal.pcbi.1000900

26. Metzcar J, Wang Y, Heiland R, Macklin P. A Review of Cell-Based Computational Modeling in Cancer Biology. JCO Clin Cancer Inform. 2019;3: 1–13.

27. Macklin P. Key challenges facing data-driven multicellular systems biology. arXiv:180604736 [q-bioQM]. 2018;

28. Yankeelov TE, Quaranta V, Evans KJ, Rericha EC. Toward a Science of Tumor Forecasting for Clinical Oncology [Internet]. Cancer Research. 2015. pp. 918–923. doi:10.1158/0008-5472.can-14-2233

29. Hutchinson L, Steiert B, Soubret A, Wagg J, Phipps A, Peck R, et al. Models and Machines: How Deep Learning Will Take Clinical Pharmacology to the Next Level. CPT Pharmacometrics Syst Pharmacol. 2019;8: 131–134.

30. Shmulevich I, Dougherty ER. Probabilistic Boolean Networks: The Modeling and Control of Gene Regulatory Networks. SIAM; 2010.

31. Vundavilli H, Datta A, Sima C, Hua J, Lopes R, Bittner ML. In Silico Design and Experimental Validation of Combination Therapy for Pancreatic Cancer. IEEE/ACM Trans Comput Biol Bioinform. 2018; doi:10.1109/TCBB.2018.2872573

32. Layek R, Datta A, Bittner M, Dougherty ER. Cancer therapy design based on pathway logic. Bioinformatics. 2011;27: 548–555.

33. Shmulevich I, Dougherty ER, Kim S, Zhang W. Probabilistic Boolean Networks: a rule-based uncertainty model for gene regulatory networks. Bioinformatics. 2002;18: 261–274.

34. Maire T, Youk H. Molecular-Level Tuning of Cellular Autonomy Controls the Collective Behaviors of Cell Populations. Cell Syst. 2015;1: 349–360.

35. Berg HC. Random Walks in Biology. 2018. doi:10.2307/j.ctv7r40w6

36. Thomas M. A Generalization of Poisson's Binomial Limit For use in Ecology. Biometrika. 1949. p. 18. doi:10.2307/2332526

37. Jones-Todd CM, Caie P, Illian JB, Stevenson BC, Savage A, Harrison DJ, et al. Identifying prognostic structural features in tissue sections of colon cancer patients using point pattern analysis. Statistics in Medicine. 2019. pp. 1421–1441. doi:10.1002/sim.8046

38. Kang S, Kahan S, McDermott J, Flann N, Shmulevich I. Biocellion: accelerating computer simulation of multicellular biological system models. Bioinformatics. 2014;30: 3101–3108.

39. Wang Q, Miskov-Zivanov N, Liu B, Faeder JR, Lotze M, Clarke EM. Formal Modeling and Analysis of Pancreatic Cancer Microenvironment. Computational Methods in Systems Biology. 2016. pp. 289–305. doi:10.1007/978-3-319-45177-0_18

40. Cancer Genome Atlas Research Network. Integrated Genomic Characterization of Pancreatic Ductal Adenocarcinoma. Cancer Cell. 2017;32: 185–203.e13.

41. Arango Duque G, Descoteaux A. Macrophage cytokines: involvement in immunity and infectious diseases. Front Immunol. 2014;5: 491.

42. Hao N-B, Lü M-H, Fan Y-H, Cao Y-L, Zhang Z-R, Yang S-M. Macrophages in Tumor Microenvironments and the Progression of Tumors. Clinical and Developmental Immunology. 2012. pp. 1–11. doi:10.1155/2012/948098

43. Wherry EJ, John Wherry E, Kurachi M. Molecular and cellular insights into T cell exhaustion [Internet]. Nature Reviews Immunology. 2015. pp. 486–499. doi:10.1038/nri3862

44. Wherry EJ, John Wherry E. T cell exhaustion. Nature Immunology. 2011. pp. 492–499. doi:10.1038/ni.2035

45. Ahn J, Yuan Y, Parmigiani G, Suraokar MB, Diao L, Wistuba II, et al. DeMix: deconvolution for mixed cancer transcriptomes using raw measured data. Bioinformatics. 2013. pp. 1865–1871. doi:10.1093/bioinformatics/btt301

46. van Laarhoven PJM, Aarts EHL. Performance of the simulated annealing algorithm. Simulated Annealing: Theory and Applications. 1987. pp. 77–98. doi:10.1007/978-94-015-7744-1_6

47. Kirkpatrick S, Gelatt CD Jr, Vecchi MP. Optimization by simulated annealing. Science. 1983;220: 671–680.

48. Baron M, Veres A, Wolock SL, Faust AL, Gaujoux R, Vetere A, et al. A Single-Cell Transcriptomic Map of the Human and Mouse Pancreas Reveals Inter- and Intra-cell Population Structure. Cell Syst. 2016;3: 346–360.e4.

49. Danziger SA, Gibbs DL, Shmulevich I, McConnell M, Trotter MWB, Schmitz F, et al. ADAPTS: Automated Deconvolution Augmentation of Profiles for Tissue Specific cells. doi:10.1101/633958

50. Ellrott K, Bailey MH, Saksena G, Covington KR, Kandoth C, Stewart C, et al. Scalable Open Science Approach for Mutation Calling of Tumor Exomes Using Multiple Genomic Pipelines. Cell Syst. 2018;6: 271–281.e7.

51. Vonlaufen A, Joshi S, Qu C, Phillips PA, Xu Z, Parker NR, et al. Pancreatic stellate cells: partners in crime with pancreatic cancer cells. Cancer Res. 2008;68: 2085–2093.

52. Marino S, Hogue IB, Ray CJ, Kirschner DE. A methodology for performing global uncertainty and sensitivity analysis in systems biology. J Theor Biol. 2008;254: 178–196.

53. Longabaugh WJR. BioTapestry: a tool to visualize the dynamic properties of gene regulatory networks. Methods Mol Biol. 2012;786: 359–394.

54. Erkan M, Michalski CW, Rieder S, Reiser-Erkan C, Abiatari I, Kolb A, et al. The activated stroma index is a novel and independent prognostic marker in pancreatic ductal

adenocarcinoma. Clin Gastroenterol Hepatol. 2008;6: 1155–1161.

55. Fujita H, Ohuchida K, Mizumoto K, Nakata K, Yu J, Kayashima T, et al. alpha-Smooth Muscle Actin Expressing Stroma Promotes an Aggressive Tumor Biology in Pancreatic Ductal Adenocarcinoma. Pancreas. 2010;39: 1254–1262.

56. Blaine SA, Ray KC, Branch KM, Robinson PS, Whitehead RH, Means AL. Epidermal growth factor receptor regulates pancreatic fibrosis. Am J Physiol Gastrointest Liver Physiol. 2009;297: G434–41.

57. Hänzelmann S, Castelo R, Guinney J. GSVA: gene set variation analysis for microarray and RNA-seq data. BMC Bioinformatics. 2013;14: 7.

58. Bailey P, Chang DK, Nones K, Johns AL, Patch A-M, Gingras M-C, et al. Genomic analyses identify molecular subtypes of pancreatic cancer. Nature. 2016;531: 47–52.

59. Moffitt RA, Marayati R, Flate EL, Volmar KE, Loeza SGH, Hoadley KA, et al. Virtual microdissection identifies distinct tumor- and stroma-specific subtypes of pancreatic ductal adenocarcinoma. Nat Genet. 2015;47: 1168–1178.

60. Traynard P, Tobalina L, Eduati F, Calzone L, Saez-Rodriguez J. Logic Modeling in Quantitative Systems Pharmacology. CPT Pharmacometrics Syst Pharmacol. 2017;6: 499–511.

61. Perfetto L, Briganti L, Calderone A, Perpetuini AC, Iannuccelli M, Langone F, et al. SIGNOR: a database of causal relationships between biological entities. Nucleic Acids Research. 2016. pp. D548–D554. doi:10.1093/nar/gkv1048

Figure1

Click here to download Figure Figure1.pdf

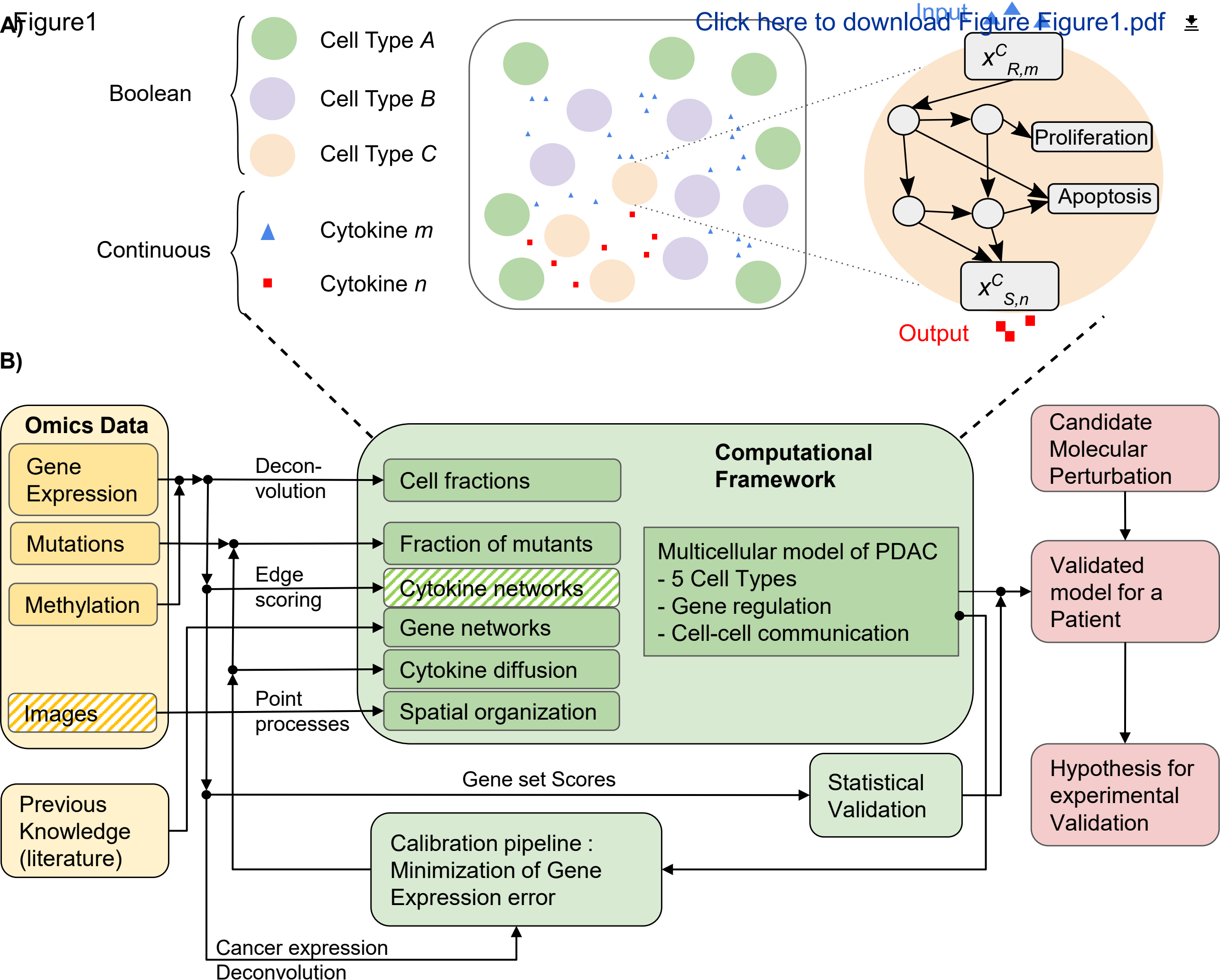

Figure2

Click here to download Figure Figure2.pdf

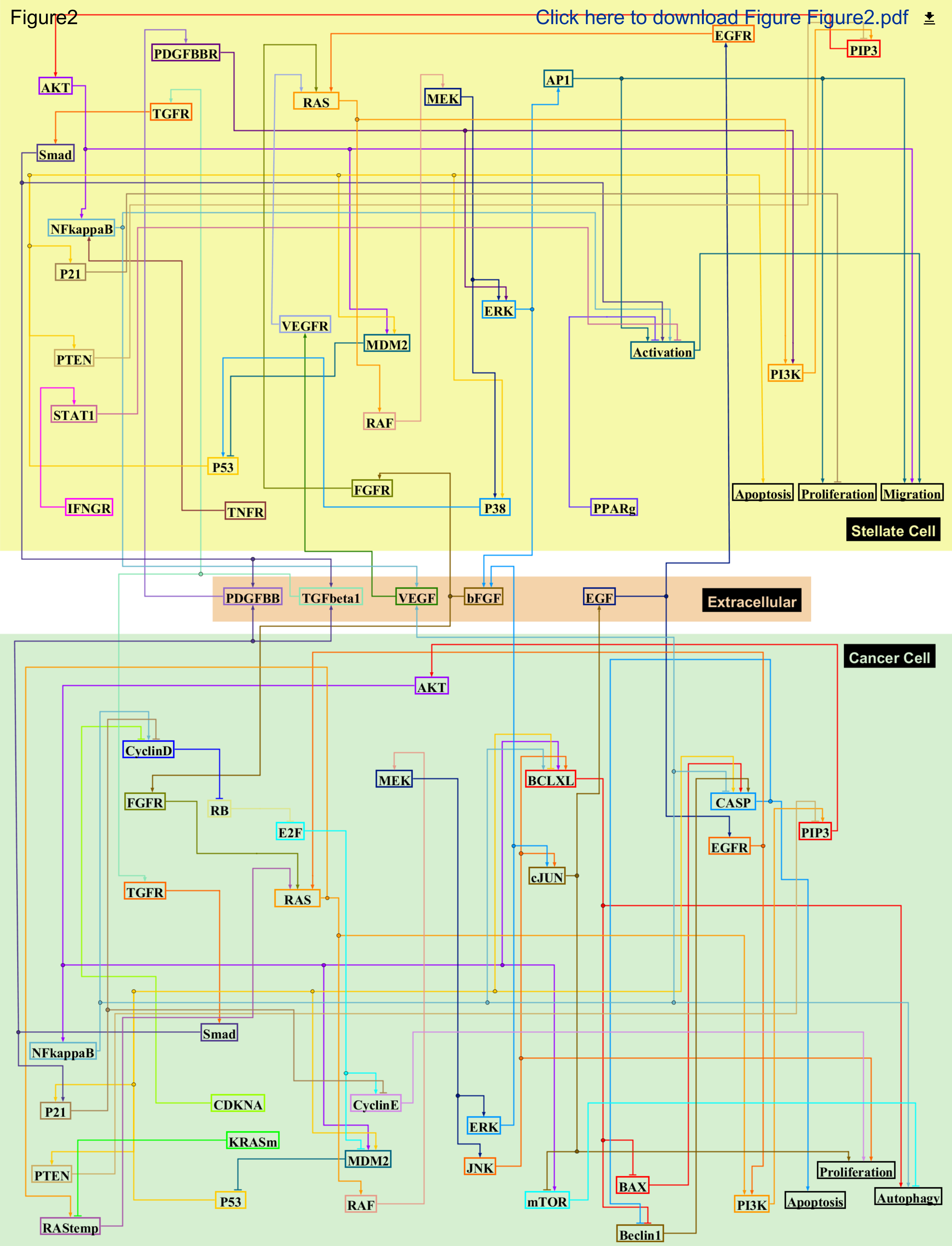

Figure3

Click here to download Figure Figure3.pdf

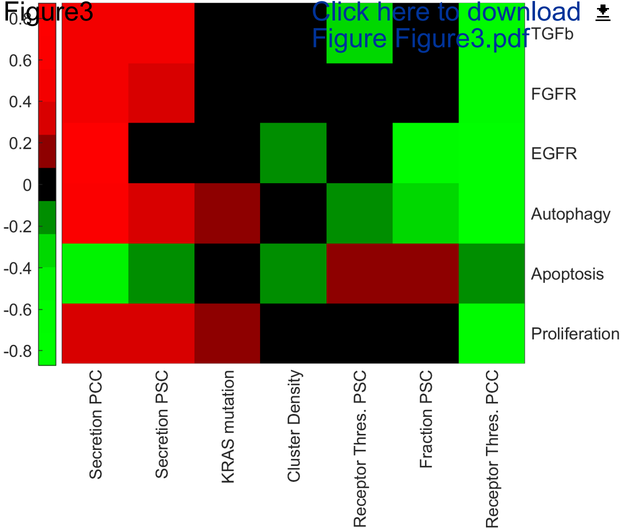

Figure4

Click here to download Figure Figure4.pdf

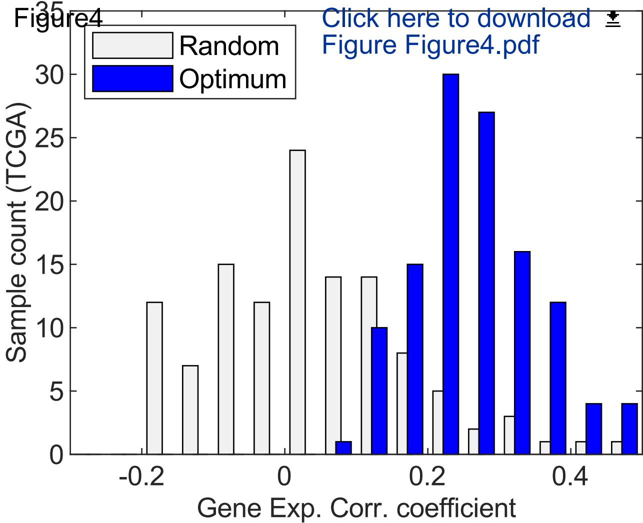

Figure5

Click here to download Figure Figure5.pdf

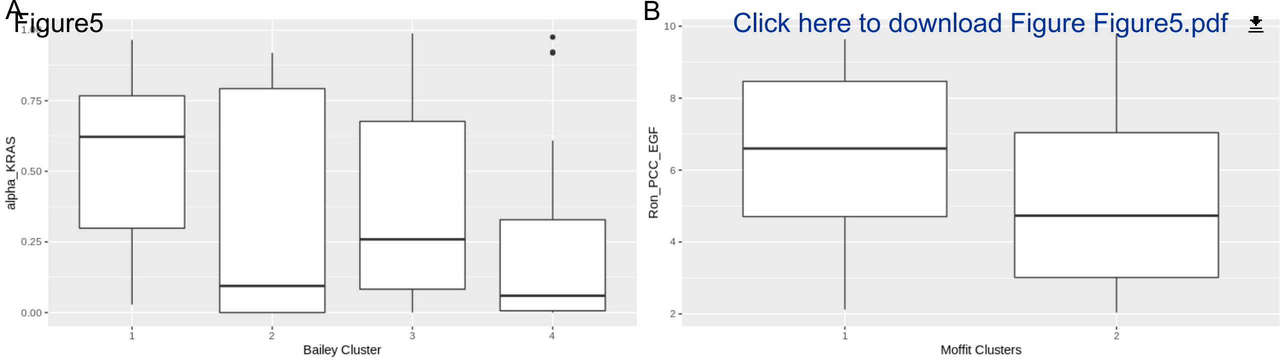

Figure6

Click here to download Figure Figure6.pdf

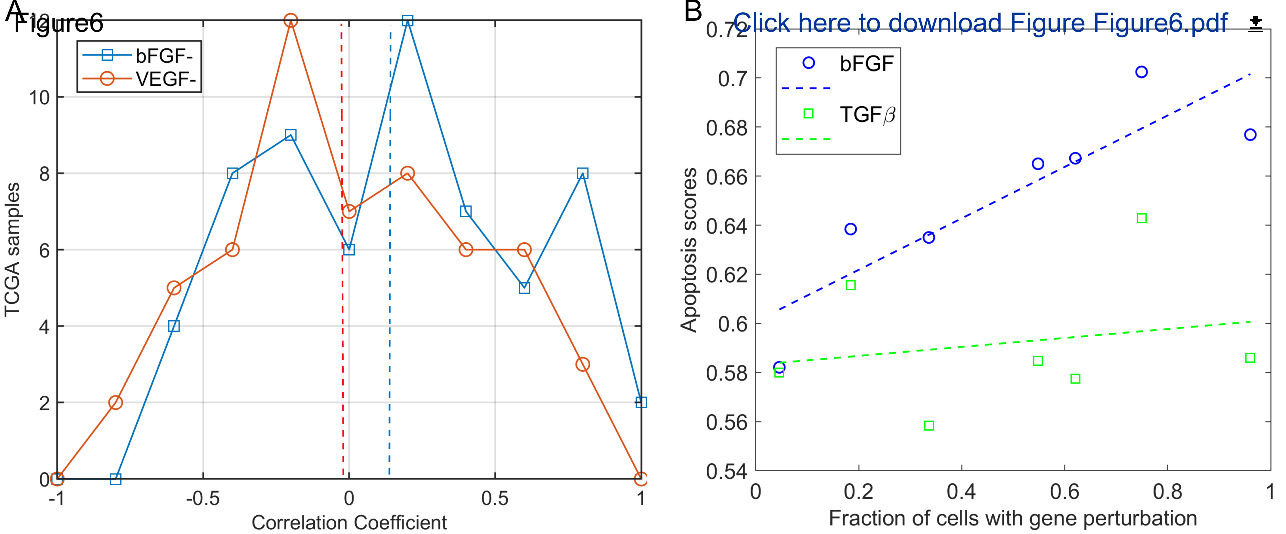

Additional File 1

Click here to access/download
Supplementary Material
Supporting Material PDAC Manuscript.pdf

Additional File 2

Click here to access/download
Supplementary Material
SupplementaryData_Pancreatic_Cell_Signatures.tsv
