# Supplementary material for: A generalizable data-driven multicellular model of pancreatic ductal adenocarcinoma
Source: Gigascience. 2020 Jul 22;9(7):giaa075. doi: 10.1093/gigascience/giaa075 (PMC7374045; doi:10.1093/gigascience/giaa075)
Supplement: giaa075_GIGA-D-19-00272_Revision_2 [file giaa075_giga-d-19-00272_revision_2.pdf]

GigaScience

A generalizable data-driven multicellular model of pancreatic ductal adenocarcinoma

--Manuscript Draft--

**Manuscript Number:**	GIGA-D-19-00272R2
**Full Title:**	A generalizable data-driven multicellular model of pancreatic ductal adenocarcinoma
**Article Type:**	Research
**Funding Information:**	
**Abstract:**	Background<br>Mechanistic models, when combined with pertinent data, can improve our knowledge regarding important molecular and cellular mechanisms found in cancer. These models make the prediction of tissue level response to drug treatment possible, which can lead to new therapies and improved patient outcomes. Here we present a data-driven multiscale modeling framework to study molecular interactions between cancer, stromal, and immune cells found in the tumor microenvironment. We also develop methods to use molecular data available in The Cancer Genome Atlas (TCGA) to generate sample-specific models of cancer.<br>Results<br>By combining published models of different cells relevant to pancreatic ductal adenocarcinoma (PDAC), we built an agent-based model of the multicellular pancreatic tumor microenvironment, formally describing cell type-specific molecular interactions and cytokine mediated cell-cell communications. We used an ensemble-based modeling approach to systematically explore how variations in the tumor microenvironment affect the viability of cancer cells. The results suggest that the autocrine loop involving EGF signaling is a key interaction modulator between pancreatic cancer and stellate cells. EGF is also found to be associated with previously described subtypes of PDAC. Moreover, the model allows a systematic exploration of the effect of possible therapeutic perturbations; our simulations suggest that reducing bFGF secretion by stellate cells will have, on average, a positive impact on cancer apoptosis.<br>Conclusions<br>The developed framework allows model-driven hypotheses to be generated regarding therapeutically relevant PDAC states with potential molecular and cellular drivers indicating specific intervention strategies.
**Corresponding Author:**	Boris Aguilar<br>Institute for Systems Biology<br>Seattle, WA UNITED STATES
**Corresponding Author Secondary Information:**	
**Corresponding Author's Institution:**	Institute for Systems Biology
**Corresponding Author's Secondary Institution:**	
**First Author:**	Boris Aguilar
**First Author Secondary Information:**	
**Order of Authors:**	Boris Aguilar
	David L Gibbs
	David L Reiss
	Mark McConnell
	Samuel A Danziger
	Andrew Dervan
	Matthew Trotter
	Douglas Bassett
	Rob Hershberg

Powered by Editorial Manager® and ProduXion Manager® from Aries Systems Corporation

	Alexander V Ratushny
	Ilya Shmulevich
**Order of Authors Secondary Information:**	
**Response to Reviewers:**	Dear Dr. Nogoy,<br><br>Thank you for giving us the opportunity to submit a revised draft of the manuscript entitled “A generalizable data-driven multicellular model of pancreatic ductal adenocarcinoma” to the “Data-Driven Multicellular Systems Biology” thematic series of GigaScience.<br><br>We appreciate the time and effort that you and the reviewers have dedicated to provide us with valuable feedback on our manuscript.<br><br>Regarding your suggestion concerning Biocellion, we added a statement (see Availability of source code and requirements) describing that the code is self-contained, that all the dependencies are in the repository, including Biocellion1.2 which is free for academic use, making it unnecessary for users to download or install Biocellion; and that the code is ready to be compiled and executed.<br><br>Furthermore, we ported the code to CodeOcean and included a reproducibility statement in the paper (https://doi.org/10.24433/CO.2337238.v1). The capsule includes a toy example of the model as well as the scripts to run the two models described in the paper.<br><br>We are also attaching with this letter a response to reviewer #2. We look forward to hearing from you regarding our submission and to respond to any further questions and comments you may have.<br><br>Sincerely,<br><br>Ilya Shmulevich<br>Alexander Ratushny<br><br><br>Reviewer #2: I'm generally happy with the revision and the deposition of the source code, however it is still not clear if this can be run completely independently of the Biocellion environment. I could not find a clear statement on this. This is important and pointed out by more reviewers. If this is fully clarified, and any dependency has been eliminated so that the code can be run irrespective of Biocellion, I'm happy that this complies with the premises of the journal. Alternatively access to Biocellion source code should be provided for the relevant parts.<br><br>The code that accompanies the paper is open, however it needs Biocellion to run and generate the simulations; this is similar to a code in Matlab which needs the MATLAB framework to run. except that the Biocellion framework is free for academic use.<br>To make the code self-contained and easier for users to run simulations, the biocellion framework and some dependencies are included in the github repository. This way users don’t need to install Biocellion.<br>Moreover, for reproducibility and to make it easier for users to try the code we created a capsule in OceanCode which includes all the executable code (https://doi.org/10.24433/CO.2337238.v1), and dependencies. Code Ocean makes sure that code is reproducible.<br>We added a statement about dependencies and the link of the Code Ocean capsule in the “Availability of source code and requirements” section.
**Additional Information:**	
**Question**	**Response**
Are you submitting this manuscript to a special series or article collection?	No
**Experimental design and statistics**	Yes

Full details of the experimental design and statistical methods used should be given in the Methods section, as detailed in our Minimum Standards Reporting Checklist. Information essential to interpreting the data presented should be made available in the figure legends.<br><br>Have you included all the information requested in your manuscript?	
**Resources**<br><br>A description of all resources used, including antibodies, cell lines, animals and software tools, with enough information to allow them to be uniquely identified, should be included in the Methods section. Authors are strongly encouraged to cite Research Resource Identifiers (RRIDs) for antibodies, model organisms and tools, where possible.<br><br>Have you included the information requested as detailed in our Minimum Standards Reporting Checklist?	Yes
**Availability of data and materials**<br><br>All datasets and code on which the conclusions of the paper rely must be either included in your submission or deposited in publicly available repositories (where available and ethically appropriate), referencing such data using a unique identifier in the references and in the "Availability of Data and Materials" section of your manuscript.<br><br>Have you have met the above requirement as detailed in our Minimum Standards Reporting Checklist?	Yes

Manuscript with DOI
Click here to access/download;Manuscript;PDAC Manuscript GigaScienceRevision DOI.docx

A generalizable data-driven multicellular model of pancreatic ductal adenocarcinoma

Authors:

Boris Aguilar[1] boris.aguilar@systemsbiology.org

David L Gibbs[1], david.gibbs@systemsbiology.org

David L Reiss[2], dreiss@celgene.com

Mark McConnell[2], mmcconnell@celgene.com

Samuel A Danziger[2], sdanziger@celgene.com

Andrew Dervan[2], adervan@celgene.com

Matthew Trotter[3], mtrotter@celgene.com

Douglas Bassett[2], dbassett@celgene.com

Rob Hershberg[2], rhershberg@celgene.com

Alexander V Ratushny[2*], aratushny@celgene.com

Ilya Shmulevich[1*], ilya.shmulevich@systemsbiology.org

* corresponding authors

Affiliations:

[1]Institute for Systems Biology, Seattle WA, 98109, USA

[2]Bristol-Myers Squibb, Summit, NJ, USA

[3]Celgene Institute for Translational Research Europe (CITRE), Seville, Spain, Celgene Corporation, a Bristol-Myers Squibb Company, Summit, NJ

Abstract

Background

Mechanistic models, when combined with pertinent data, can improve our knowledge regarding important molecular and cellular mechanisms found in cancer. These models make the prediction of tissue level response to drug treatment possible, which can lead to new therapies and improved patient outcomes. Here we present a data-driven multiscale modeling framework to study molecular interactions between cancer, stromal, and immune cells found in the tumor microenvironment. We also develop methods to use molecular data available in The Cancer Genome Atlas (TCGA) to generate sample-specific models of cancer.

Results

By combining published models of different cells relevant to pancreatic ductal adenocarcinoma (PDAC), we built an agent-based model of the multicellular pancreatic tumor microenvironment, formally describing cell type-specific molecular interactions and cytokine mediated cell-cell communications. We used an ensemble-based modeling approach to systematically explore how variations in the tumor microenvironment affect the viability of cancer cells. The results suggest that the autocrine loop involving EGF signaling is a key interaction modulator between pancreatic cancer and stellate cells. EGF is also found to be associated with previously described subtypes of PDAC. Moreover, the model allows a systematic exploration of the effect of possible therapeutic perturbations; our simulations suggest that reducing bFGF secretion by stellate cells will have, on average, a positive impact on cancer apoptosis.

Conclusions

The developed framework allows model-driven hypotheses to be generated regarding therapeutically relevant PDAC states with potential molecular and cellular drivers indicating specific intervention strategies.

Keywords

Cancer modeling, data-driven model, pancreatic ductal adenocarcinoma, multicellular model

Introduction

Pancreatic ductal adenocarcinoma (PDAC), the most common form of pancreatic cancer, is the fourth leading cause of cancer associated death in the United States and is predicted to be the second in 2030 [1]. With a 5-year survival rate of only 3%, it has a very poor prognosis. Across all types of cancer, it is becoming increasingly clear that interactions within the tumor microenvironment (TME) have a strong effect on tumor growth. This is particularly relevant for PDAC research where previous studies have revealed high heterogeneity and complexity in the tumor microenvironment, where a mixture of interacting immune cells, stromal tissue and cancer cells are resident. However, much remains to be known regarding how differences in the TME affect the behavior of cancer cells. For instance, there is a debate concerning whether stroma-cancer interactions are associated with progression of pancreatic cancer or, rather, provide protective measures [2]. Thus, to make progress in the treatment of PDAC, new strategies must be developed to improve our understanding of the effects of the tumor microenvironment on cancer states and progression.

In silico models are frequently used in systems biology for the discovery of general principles and novel hypotheses [3–5]. Moreover, it is eventually possible that when combined with relevant data, *in silico* models will be able to make predictions with sufficient accuracy for therapeutic treatment. Despite their potential, concrete examples of predictive models of cancer progression are scarce. One reason is that most models have focused on single cell type dynamics, ignoring the interactions between cancer cells and their local microenvironment. Indeed, there have been a number of models that were used to study gene regulation at the single cell scale, such as macrophage differentiation [6–8], T cell exhaustion [9], differentiation and plasticity of T helper cells [10,11], cell cycle [12–14], and regulation of key genes in different tumor types [15].

Although not as numerous as single cell type models, multicellular models have progressively been developed to study different aspects of cancer biology, including tumor immunosurveillance [16–20], hypoxia [21,22], angiogenesis [23,24], and epithelial-mesenchymal transition [25,26], among others; we refer the reader to Metzcar et al. [27] for a recent and comprehensive review. Typically, these models are based on phenomenological rules to model cell behavior and therefore use limited data to calibrate their parameters. Although multicellular models are being increasingly used in cancer biology, there remains a need for a modeling framework that is capable of integrating different multiscale properties of the TME, such as molecular and cellular heterogeneity and non-uniform spatial distributions of cells, with the capacity to leverage diverse -omics datasets for model building, calibration and validation, allowing researchers to explore novel molecular therapies *in silico [3,28–30]*.

In this work, we developed a modeling framework designed to study the interaction between cancer cells and their microenvironment. Figure 1 shows a schematic of the modeling framework. The framework is a combination of two well established approaches: Boolean Networks [31] (BNs) and Agent Based Modeling [27] (ABM), used at the molecular and cellular levels, respectively. The cancer signaling and regulatory networks are modeled with BNs, while ABM is used to simulate intercellular networks consisting of different cell types and intercellular signaling molecules. We used BNs because of their efficient and simple formulation that minimizes the number of parameters in the multicellular model. This vertical (“multiscale”) integration, using ABM and BNs, enables the exploration of therapeutic interventions on the molecular level for inducing transitions of the tumor into less proliferative states, while utilizing currently available high-throughput molecular data.

Voukantsis et al. [32] proposed a multicellular model for tumor growth in which cells are placed in a lattice. Each cell is endowed with a Boolean network that controls cellular actions, such as proliferation and apoptosis, that are key for tumor growth. Letort et al. [33] integrated stochastic Boolean signaling networks into agent-based models by combining MaBoSS [34,35],

an open source package for Boolean networks, with PhysiCell [17], an ABM based simulation platform. The main goal of the previous ABM/BN combinations was the simulation of tumor growth, which requires not only parameters that regulate cell-cell communication and intracellular gene regulation, but also parameters for cell division, cell death, oxygen uptake, mechanical interactions, ECM properties, etc., resulting in highly complex models that require data currently not available for validation and calibration [36]. In this article, our focus is modeling how the cancer cell state is affected by communication with other cells in the tumor microenvironment. Therefore, we included model components, such as gene regulation, cell proportions, and cellular spatial distributions, that can be directly compared with commonly used omics and imaging data, aiming at integration between the model and experimental data needed in cancer research [28].

We built a network of cell type-specific intracellular interactions and cytokine mediated intercellular communications, by combining published models of different cell types relevant to PDAC, namely, the ductal cancer cells, stellate cells, $CD4^+$ T cells, $CD8^+$ T cells, and macrophages. Through computational simulations, using an ensemble modeling approach whereby multiple simulations are aggregated into statistically summarized results, this framework was used to study how the tumor microenvironment, characterized by a set of cytokines, stromal cells, and somatically heterogeneous cancer cells, affects the viability of cancer cells.

Modeling framework

In this section, we describe our approach to model a block of cancerous tissue with a mixture of cancer, stromal, and immune cells randomly located inside a 3D rectangular simulation domain (Figure 1). Each cell contains a Boolean network that determines its cellular phenotype (functional state), such as proliferation or apoptosis, the possible secretion of cytokines, and the state of membrane receptors. The model is built on the following assumptions and considerations:

- Since our main goal was to study the interplay between cell-cell communication and gene regulation, other interactions and processes, such as cell motility and mechanical interactions, were not included in the model. Moreover, the model simulations focus on a time window relevant to cell signaling and gene regulation which is a few hours. Considering these time scales, we assumed that the number of cells and the initial positions of cells do not change during simulations.
- The model uses two-time scales, one for gene regulation and one for cell-cell communication. Although they are biologically related, we assume cell communication takes place on a faster time scale than gene regulation.
- The parameters that characterize cell behavior are the same for all cells of a given type. Thus, all cells of a single type are governed by the same BN and share the same parameters of cell communications.

The following subsections present a detailed description of each component of our modeling approach:

Cells as Boolean networks Signal transduction and gene regulation in a given cell is modeled with synchronous BNs, a well-known modeling approach used to study several cellular processes important in cancer [37,38]. Synchronous because all nodes in the BN (in all cells) are updated simultaneously at each time step. The BN of a cell i is defined on a set of n binary-valued variables $X_i = \{x_1^i, \ldots, x_n^i\}$, where a node $x_j^i \in \{0,1\}$ represents the expression of a gene, a cellular behavior, or secretion of a cytokine to the TME. The binary vector X_i represents the phenotypic state of cell i. Thus, for a cellular BN of n nodes, there are 2^n possible states. We divided the binary nodes x_j^i into two groups: signal receptors and regulatory nodes. Receptor nodes sense the presence of signaling molecules in the local TME, with their updating rules being specified in the next

subsection. Regulatory nodes are updated in discrete time steps by conventional logic rules. Specifically, the regulatory node j of a cell i at time step $t+1$(i.e. the next time step) is determined by the values of the nodes ("genes") , $x_{j_1}^i, x_{j_2}^i, \ldots, x_{j_{k_{j,i}}}^i$ at time t by means of the Boolean function, $F_j^i\colon \{0,1\}^{k_{j,i}} \rightarrow \{0,1\}$. There are $k_{j,i}$ nodes assigned as inputs to regulatory node x_j^i, thereby determining the wiring of the BN. Thus, the Boolean value of a regulatory node x_j^i is given by

$$x_j^i(t+1) = F_j^i(\, x_{j_1}^i(t), \ldots, x_{j_{k_{j,i}}}^i(t)\,) \qquad (1)$$

It is worth noting that regulatory genes of all cells are updated synchronously using the states of nodes of the same cell, whereas membrane receptors are updated by the TME, that is, by the presence of cytokines right before the update of regulatory genes. Moreover, cells of the same type are regulated by the same set of Boolean functions. Thus, all cells of type I are regulated by $\{\, F_1^I, F_2^I, \ldots\}$ which do not change during simulations. These regulatory functions represent existing knowledge of intracellular gene regulation in given cell type and are typically obtained from literature.

Additionally, to model stochastic dynamics, following the convention used in random Boolean networks [31,39,40], we introduce a perturbation probability q and a random perturbation vector, $\gamma = [\, \gamma_1, \gamma_2, \ldots, \gamma_n\,]$, where $\gamma_j \in \{0,1\}$ and $P\{\gamma_j = 1\} = q$, such that:

$$X_i(t+1) = X_i(t) \oplus \gamma, \text{ with probability } (1-(1-q)^n)$$

$$X_i(t+1) = [\, F_1^i, F_2^i, \ldots, F_n^i\,], \text{ otherwise,}$$

where $\oplus$ indicates the modulo-2 sum. The fact that any state transition has a nonzero probability under this perturbation model implies that the dynamics of the network are described by an ergodic Markov chain with a (unique) steady-state distribution [40,41]. It is worth noting that we use the same gamma value ($\gamma_j = q$) for all the genes regardless of the cell type.

Some of the regulatory nodes are associated with important cellular behaviors, such as proliferation, apoptosis, or migration. Moreover, some of the regulatory nodes are associated with

the secretion of cytokines in such a way that a state of 0 or 1 of these nodes corresponds to low or high rates of secretion, respectively.

Cell-cell communication via diffusion of cytokines We include communication between cells by modeling the secretion, sensing, and diffusion of cytokines. The formulation of cell-cell communication is similar to the model developed by Olimpio et al. [42]. For simplicity we made the following assumptions. First, the concentration of cytokines is not affected by cellular uptake of molecules. Second, the cytokine diffusion is much faster than gene regulation.

A cell i releases cytokine m with a secretion rate of $\eta_m^i(x_{S_m}^i)$ molecules per time step, which depends on the Boolean state of its designated signal node $x_{S_m}^i$(S_m is the label of one of the regulatory nodes of cell i). We assume that $\eta_m^i(0) = 1$ and $\eta_m^i(1) = R_m^i$, $R_m^i > 1$, to account for basal and active expression, respectively. We make this assumption with no loss of generality since it is equivalent to normalizing active expression by the lower basal expression [42]. The concentration, C, of cytokine m changes in space and time according to a diffusion degradation equation. For cells randomly scattered in a regular 3D lattice, the concentration of cytokine m in a voxel v is approximated by solving the following diffusion degradation equation with periodic boundary conditions:

$$\partial C_m^v / \partial t = D \vartriangle C_m^v - \gamma_D C_m^v + h^{-3} \sum_{i \in v} \eta_m^i(x_{S_m}^i) \quad (2)$$

for each voxel v of the lattice containing the set of cells. D is the diffusion coefficient, γ_D is the constant degradation rate, and h is grid spacing used to solve the diffusion degradation equation by the finite difference method. Assuming that diffusion is much faster than gene regulation, we use the steady state of the diffusion equation above,

$$0 = D \vartriangle C_m^v - \gamma_D C_m^v + h^{-3} \sum_{i \in v} \eta_m^i(x_{S_m}^i) \quad (3)$$

and use a numerical solver for calculating C_m^v in simulations. An important component of the steady state solution is the effective interaction distance, λ, where $\lambda = \sqrt{D/\gamma_D}$ [43,44].

Integration of gene regulation and cell-cell communication The coupling between signal diffusion and BNs was adapted from Olimpio et al. [42] where a cellular automata model was used to analyze the consequences of cell-cell communication. Figure 1 shows a representation of the integration between BNs and cell-to-cell signaling. The cellular BNs can influence the spatial distribution of cytokines. A cytokine p is secreted by cell i with secretion rate R_p^i(high) or 1 (low) according to the Boolean state of an output node of its BN, $x_{S,p}^i$ in Figure 1.

The concentration of cytokines can influence the behavior of cellular BNs. To sense cytokine m, cell i checks the local concentration of the signal, i.e., the concentration at its containing voxel. If the local concentration of m is above a threshold value, K_m^i , then the signal receptor is activated, otherwise it is deactivated. This is depicted by the blue triangles in Figure 1. Formally, the state of the receptor node $x_{R,m}^i$ of cell i, located in voxel v, follows the equations:

$$x_{R,m}^i(t+1) = 1, if\ C_m^v(t) > K_m^i, \quad (4)$$

$$x_{R,m}^i(t+1) = 0, \text{ otherwise,}$$

where C_v^m is the concentration of m in voxel v that contains cell i. The thresholds K_m^i are parameters of the model that characterize the sensitivity of cells to cytokine concentration. All cells of the same type share the same activation threshold associated with a given cytokine.

Note that while our model assumes diffusion-based cell-cell communication, the effective interaction distance can be shortened, such that the system behaves as if signaling were contact-mediated, the latter effectively being a special case of the former. This is possible by setting a spacing resolution (h) equal to cell diameter, such that changes in concentration between nearest neighbor cells can be captured by the model of signal diffusion.

Tissue Architecture We constructed a lattice free model tissue as a 3D point process of cells, each represented by a Boolean network and a spatial point in a rectangular block of size L. We

assume a fixed density of cells, ρ, and divide cell types into cancer and stromal. The density of cancer cells is $\rho_C = r_C\,\rho$ where r_C is the fraction of cancer cells in the tissue sample. The density of stromal cells is $\rho_S = (1 - r_C)\,\rho$. The positions of cancer cells were generated by a Thomas process [45] in which points are scattered around cluster centers according to a 3D Gaussian distribution with zero mean and covariance matrix $\sigma^2 I$, where I is the 3×3 identity matrix. The cluster centers are generated by a simple Poisson process with intensity ρ_{cc}. Stromal cells are generated by a Void process [46] in which points are removed if they are within a distance R_{ex} from a cluster center. The same cluster centers were used for cancer and stromal cells. The cluster centers are generated using a Poisson process with density $\rho_{cc} = s\rho_C$, where s is a parameter that determines the clustering of cancer cells.

In order to avoid unrealistic high densities of cancer cells, we used a fixed value of σ, such that the density of cells inside the sphere with radius σ is limited by a parameter ρ_{max}. We set up $\rho_{max} = 8\rho$, so that clusters of cancer cells are more concentrated than stromal cells. Figure 2A shows an example of the spatial distribution of a system with two cell types using a $s = 0.7$, and Figure S1 shows the distribution of cells for different values of s, showing that changing s changes the distribution of cancer cells from clustered to homogeneous.

Methods

Simulations and simulation framework

In a tissue model with N cells and n genes per cell, there are $2^{N\,n}$ possible states. Assuming the tissue model reaches a steady state distribution, owing to the ergodic dynamics induced by the perturbation probability q [31], the average expression of node g in cancer cells is:

$$E[f_g] = \sum_s p_s f_s(g) \quad (5)$$

where p_s is the probability of state $s \in \{1,2,\ldots,2^{N\,n}\}$ in the steady state distribution and $f_s(g)$ is the fraction of cancer cells with gene g in the ON state. Similar equations are used for the expression of other cell types of the system. The distribution of p_s depends on model parameter set θ and the Boolean network for each cell type. Since the number of possible states is very large, we need to approximate the expectation above by performing M independent simulations and considering the last K steps of each simulation. Thus, the approximation of the average expression of gene g is:

$$\hat{f}_g(\theta) = \frac{1}{MK} \sum_i^M \sum_j^K f_{s_{ij}}(g) \quad (6)$$

where $f_{s_{ij}}(g)$ is the fraction of cancer cells with active gene g in the state s_{ij} of the system in step j of simulation i. The gene expression profile of cancer cells from the simulations is:

$$\overline{G}(\theta) = \{\hat{f}_1(\theta), \hat{f}_2(\theta), \ldots, \hat{f}_n(\theta)\} \quad (7)$$

Simulations of our model were implemented in *Biocellion* [47], a high-performance computing platform designed for simulation of multicellular systems. At every time step t of the simulation, the concentration of signaling molecule m is updated by numerically solving equation (3), after which the Boolean states of the cells are updated using the computed concentrations. Figure 2A shows the spatial cellular distribution of a system with two cell types (Pancreatic cancer cells and Stellate cells). Figure 2B shows how the fraction of cancer cells with activated proliferation and apoptosis nodes changes during the simulation; the proportion of cells reaches a steady state after approximately 25 time steps. The standard deviations and averages of cell-fractions were computed from 10 independent simulations using the same parameter values. In remaining sections, if the values are not specified, then results were collected from $M = 20$ independent simulations of 400 time steps, using the last $K = 200$ time steps.

Within the proposed model, the phenotype of the tissue segment is characterized by the average proportion of cells with the corresponding phenotypic node in the ON state (activated);

for instance, the cancer proliferation phenotype of the two cell type system in Figure 3 is estimated by averaging the fraction of cancer cells with an activated proliferation node over the last 50 steps of the simulation, which is the time windows in which the system is stable, see Figure 2B.

Boolean networks

Cancer and stellate cells The Boolean networks of pancreatic cancer cells (PCC) and pancreatic stellate cells (PSC) were obtained from Wang et al. [48]. The network includes pathways that were found to be important in PDAC progression, such as the RAS-ERK and PI3K-AKT, TGFβ-SMAD4 and p53 signaling. The network also includes pathways that are important for activation of stellate cells. The cytokines that are used to communicate between these two Boolean networks are also available in Wang et al. [48]. Further, we have modified the model in order to include relevant mutations of PDAC cells including KRAS, TP53, CDKN2A, and SMAD4 mutations, which are present in more than 30% of the PDAC patient samples in The Cancer Genome Atlas (TCGA) [49]. The effect of mutations is modeled by permanently setting nodes to ON or OFF, depending on whether the mutation is functionally activating or inactivating. The mutations are applied to a randomly selected fraction of cancer cells, which in our model is characterized by a parameter (α). Moreover, we have removed the HER2-JAK1-STAT pathway as mutations in HER2 only appear in a small number of TCGA PDAC samples.

$CD4^+$ T cells The Boolean network for $CD4^+$ cells was obtained from Tieri et al. [11] which model the differentiation of naive $CD4^+$ T cells into four commonly characterized subtypes: three effector cells, Th1, Th2, and Th17, and regulatory cells, Tregs. Each subtype secretes specific sets of cytokines that can influence the behaviors of other cells. The model includes cytokines such as IFNγ secreted by Th1 subtypes, IL-10 and IL-4 secreted by Th2, and IL-17 and IL-6 secreted by Th17.

Macrophages We implemented the Boolean network model of macrophage cells developed by Palma et al. [6]. Their BN models macrophage differentiation into 4 commonly characterized subtypes: the immunogenic M1 and three immunosuppressive subtypes, M2a, M2b, and M2c. Each of these subtypes is determined by a particular set of expressed genes and cytokines including IL-12 and IL-10. We have extended the model by adding the secretion of TNF and IL-6 secreted by M1 and M2b subtypes, and TGFβ secreted by M2a and M2c [50,51].

$CD8^+$ T cells We obtained a BN model of $CD8^+$ T cells from a recently published paper by Bolouri et al. [9], in which the authors study TCR activation and the response of $CD8^+$ T cells to cytokines. They developed a BN that models the transition of T cells from naïve to acute and exhausted states in response to chronic antigen stimulation. The exhausted $CD8^+$ T cell state is characterized by high expression of immune checkpoint molecules, and lowered proliferation capacity, cytokine production, and cytotoxic activity compared to effector or memory $CD8^+$ T cells [52,53].

Parameter Calibration

Our tissue model is characterized by a set of parameters listed in the Supporting material, Table S1; some of these parameters are estimated from data available from the TCGA. Specifically, cell fractions were estimated from gene expression data using “cell deconvolution” [54]. The mutation states of patient samples were summarized from a TCGA Pan-Cancer data set [55] and deconvolved gene expression of cancer cells was generated using the DeMix algorithm [56]; see the next section below for details concerning cell fraction estimation. Most parameters were calibrated using deconvolved gene expression data. It is worth noticing that BNs are static and are not optimized.

The optimization protocol is represented in Figure S2. Our strategy is to optimize the unknown parameter set θ, including secretion rates, activation thresholds and mutation rates, by minimizing a cost function $C_p(\theta)$ defined as the deviation (ε in Figure S2) between the gene expression $G^{model}(\theta)$ of cancer cells in the model and the gene expression of cancer cells obtained from TCGA samples $G^{tcga}(p)$:

$$C_p(\theta) \; = \; \varepsilon(\, G^{model}(\theta), G^{tcga}(p) \,) \qquad (8)$$

where p is a TCGA sample. We used $\varepsilon(\, x, y) \; = \; 1 \; - \; R(x, y)$ as a cost function $C_p(\theta)$, where $R(x, y)$ is the Spearman correlation coefficient between x and y. Other alternatives of $\varepsilon(G^{model}(\theta), G^{tcga}(p) \,)$ can be tested in the future.

Thus, the optimization problem is to find the set of optimal parameters:

$$\theta_p^* = arg \; [\, min_{\theta \subset \Theta} \; C_p(\theta) \,] \qquad (9)$$

for each TCGA sample p. We used simulated annealing (SA) [57,58] to minimize $C_p(\theta)$. For our particular case, SA consists of the following steps:

1. Initialize θ_i randomly from Θ, the space of parameters listed in the Supporting material, Table S1.
2. Run W steps of the Metropolis algorithm [57] at temperature T_k. Select a new parameter θ_j from a distribution P_{ij} and compute $\Delta C_{ij} = C_p(\theta_j) \; - C_p(\theta_i)$. If $\Delta C_{ij} \leq 0$, accept the new parameter set, letting $\theta_i = \theta_j$, otherwise accept the new parameter set θ_j with probability $exp(-\Delta C_{ij}/T_k \,)$.
3. Update the temperature, $T_{k+1} = 0.8T_k$. If $T_{k+1} < \; T_{min}$then stop the algorithm, otherwise, go to step 2.

We used $P_{ij} = \; P(\theta_j | \theta_i \,) \; = \; Gaussian(\, \theta_i, \sigma(T) \,)$ where $\sigma(T) = \sigma_o T$and T is the temperature. We have used $T_o = 0.5$, $\sigma_0 = 1.0$ and $W = 60$ (number of steps in step 2) to generate the optimum parameters for each TCGA sample.

Estimation of cell fractions

Cellular deconvolution [59] was used to estimate cellular fractions from bulk RNA-seq data. In this work, we used the ADAPTs R package [54] and in particular, the SVMDECON method which makes estimations based on support vector regression. This method solves the linear model $Y = AX$, where Y is the gene expression of a given sample, and A is a matrix of gene expression signatures for each cell (in columns). This matrix (A) is typically derived from experiments where cells have been physically isolated and then measured in bulk for gene expression. However, the pancreas is composed of cell types not typically found in deconvolution resources. To create a signature matrix that includes pancreatic cells, similar to the methods found in the ADAPTS package [54], we used a pancreatic single cell RNA-seq (scRNA-seq) data set in conjunction with expression signatures for 22 immune cells (LM22) [60]. The cells found in the scRNA-seq data were previously labeled, providing a set of cells for each type. The median expression for each gene was computed by cell type, giving an expression value per gene per cell type. The goal is to produce a matrix of genes by cell types, where each signature is predictive of that particular cell type, and the matrix overall has a low condition number.

Iterating over cell types using a *t*-test, we selected genes to maximize the difference between one cell type and all others, building up the matrix. As the matrix grows in the number of genes, the condition number is also computed. The number of genes is selected to minimize the condition number. The final cell signature matrix was 566 genes for 33 cell types, with 11 cell types specific to the pancreas. The expression values were normalized first independently by data source, then merged and renormalized. The final cell signature matrix is available in the Additional file 2.

Non-metastatic pancreatic tumor data from the TCGA (PDAC) was used, providing 119 samples. The cancer cell quantities were estimated using ductal cells as a proxy, and were found to correlate with tumor purity, the proportion of cancer cells in each sample, which is calculated

from publicly available TCGA copy number variation data (Supporting material, Figure S4). A file with estimated cellular fractions for cancer cells, stellate cells, CD4+ T cells, macrophages, and CD8+ T cells is available in Additional file 3.

Mutation state of cancer cells from TCGA

For each TCGA sample, we used the MC3 Pan-Cancer somatic mutation table to generate a probability of a cancer cell having a mutated gene [55]. We compute probabilities for KRAS, TP53, CDKN2A, and SMAD4 mutations, which are present in 93%, 73%, 30% and 32% of the TCGA samples of PDAC, respectively [49]. This was done by taking the number of sequencing reads with a detected mutation and dividing that count by the number of total reads, assuming that the mutated reads come from cancer cells. Thus, for each sample and each gene, we have a probability of gene mutation. A sample level instantiation is produced by sampling from these Bernoulli distributions. A file with the presence (1) or absence (0) of mutation in TP53, CDKN2A, SMAD4, or KRAS for each TCGA sample of PDAC is available in Additional File 4.

Gene expression of cancer cells

Deconvolution of expression into portions of cancer cells and stromal (and immune) tissue compartments was performed using the DeMix software [56]. Expression values had previously been computed and were supplied by the authors of the software. A file with the expression values of cancer genes is available in Additional File 5.

Results

Analysis of the interplay of cancer and stellate cells

Previous experimental studies in mice and *in vitro* experiments [61] show that pancreatic stellate cells (PSC) promote the proliferation of pancreatic cancer cells (PCC) during the progression of disease. In this section, we use our framework to study the mechanisms that drive the interactions between these two cell types. The Boolean networks and the cytokines that regulate the phenotypic behavior of PSC and PCC were adapted from the model published by Wang et al. [48]. Figure 3 shows the network of interactions between nodes that regulate proliferation, apoptosis, and other important phenotypic behaviors of PSCs and PCCs. We used a standard sensitivity analysis [62] in which random parameter sets are generated using Latin Hypercube Sampling [62] (LHS), and used for performing simulations. Partial Ranked Correlation Coefficients [62] (PRCC) let us determine the strength of association between model parameters and important properties of tumor samples, such as cancer proliferation and apoptosis states. These properties are characterized in simulations by the average fraction of cells with the corresponding phenotypic node set to ON, see the Methods section for details.

The heatmap in Figure 4 shows the PRCC between model parameters and population level properties. The parameters considered in the sensitivity analysis, parameter ranges, and more details of model simulations are specified in the Supporting material, Table S2. We generated 1000 parameter sets using LHS and then performed 100 simulations for each parameter set using the networks in Figure 3. Each of the 100 simulations started from random initial conditions of the Boolean genes and random cellular positions. The tissue level properties were averaged over the 100 simulations.

The results suggest that the interaction between cancer and stellate cells can be harmful for cancer cells, inducing apoptosis, or helpful for cancer cells, inducing cancer proliferation. This is evident by the significant positive (red) and negative (green) correlations between model parameters and apoptosis and proliferation, as shown in Figure 4. Moreover, the results of Figure 4 show that the secretion rate of cytokines by PCCs and the sensitivity of cytokine receptors in PCCs are most associated with cancer cell behavioral states. Specifically, an increase in secreted cytokines by cancer cells trends with increases (positive correlation) in proliferation and reductions (negative correlation) in apoptosis. The secretion and the sensitivity of receptors of PSC cells also play a role in the phenotypes of cancer cells. In summary, parameters related to cell-cell communications, e.g. secretion rates and activation thresholds, have a significant impact on cancer cell behavior.

Although the correlation between cancer cluster density (a measure of spatial structure) and most phenotypic properties of cancer cells is almost zero, there are several properties that are influenced by spatial organization of cells, namely the population-level expression of EGFR and the apoptosis state of cancer cells. Thus, the spatial organization of cells, in this case the clustering of cancer cells, is another multicellular property that can potentially influence the interplay between cancer and stellate cells and should be explored in future studies.

A surprising result is the negligible correlation between the fraction of stellate cells and cancer cell proliferation. A positive correlation was expected as it has been previously reported that the stellate cells increase the survival of cancer cells [61,63,64]. This may point to the possibility that intercellular communication mechanisms between stellate and cancer cells may play a more dominant role than population numbers alone.

The role of paracrine and autocrine loops

To explore potential molecular interactions that are key in the relationship between PSC population and PCC proliferation, we have performed a sensitivity analysis after fixing the secretion rates of cancer or stellate sets. These parameters effectively change the strength of intercellular communication and autocrine loops present in both cell types (see Figure 3). First, simulations with constant and equal secretion rates of cancer and stellate cells were run ($R^{PSC} = R^{PCC} = 5$). In these simulations all paracrine and autocrine loops are allowed and were given similar weights. The results (Table 1) showed that in this case there are negligible correlations between the population of stellate cells and cancer phenotypes. When the secretion rate of R^{PSC} is greater or equal to R^{PCC}, e.g., when the signal from PSC to PCC is stronger, the correlation between the stellate fraction and cancer proliferation increases substantially. This correlation increases to 0.5 when R^{PSC} is greater or equal to R^{PCC}=2. In summary, these results suggest that asymmetric cytokine mediated communication between stellate and cancer cells plays a role in the observed positive effect on cancer survival.

	**PRCC**		
**Secretion rates**	**r_{PSC} vs proliferation**	**r_{PSC} vs apoptosis**	**r_{PSC} vs autophagy**
$R^{PSC} = R^{PCC} = 5$	0.0555	0.0718	-0.0841
$R^{PSC} > R^{PCC} = 5$	0.1173	0.1121	-0.1204
$R^{PSC} > R^{PCC} = 2$	0.4999	-0.2651	0.4406

Table 1: Partial Rank Correlation Coefficient (PRCC) between the fraction of stellate cells (r_{PSC}) and cancer phenotypes (proliferation, apoptosis, and autophagy). Simulations were performed with constant values of R^{PCC} and for different ranges of R^{PSC}. For the second and third row, 500 random values for R^{PSC} were selected in the range [R^{PCC} ,10.0].

According to the model (Figure 3), cancer cells secrete 4 cytokines, 3 of which (EGF, bFGF, TGFβ) are involved in autocrine loops. To determine the relevance of cancer autocrine loops in the stellate-cancer cells relationship, we assigned different values of secretion rates to the different cytokines secreted by cancer cells, namely R_{EGF}, R_{bFGF}, and $R_{TGF\beta}$. Table 2 shows that when only the EGF autocrine loop is active ($R_{EGF} > R_{bFGF} = R_{TGF\beta} = 2.0$) the population of stellate cells is negligibly correlated with cancer phenotypes. The correlation between stellate cell population and cancer proliferation increases to 0.3 when the bFGF autocrine loop is the only active autocrine loop. The highest (resp., lowest) correlation between stellate cell correlation and cancer proliferation (resp., apoptosis) occurs when the only autocrine loop involved is TGFβ. These results suggest that cancer cell autocrine loops that involve EGFR are key modulators of the interaction between stellate and cancer behaviors. This is consistent with the known role of EGFR in modulating the stroma to support cancer growth [65].

	**PRCC**	
**Secretion Rates**	r_{PSC} **vs proliferation**	r_{PSC} **vs apoptosis**
$R_{EGF} > R_{bFGF} = R_{TGF\beta} = 2.0$	-0.0474	0.0549
$R_{bFGF} > R_{EGF} = R_{TGF\beta} = 2.0$	0.2974	-0.0293
$R_{TGF\beta} > R_{EGF} = R_{bFGF} = 2.0$	0.5203	-0.4422

Table 2: Partial Rank Correlation Coefficient (PRCC) between fraction of stellate cells (r_{PSC}) and cancer phenotypes. Simulations were performed with different values of secretion rates of EGF, bFGF, and TGFβ secreted by cancer cells.

Patient-specific models for TCGA samples

Owing to inter-patient heterogeneity in terms of somatic alterations or tissue-level properties such as cell fractions, it is important to construct patient-specific models. Toward that end, we have developed methods for the integration of high-throughput molecular data into our modeling framework. Figure 5 shows a diagram of the analysis workflow, including the used data types from TCGA (yellow), and the methods (arrow labels in Figure 5) for integrating the data and existing knowledge into the process of initialization, parameter calibration, and model validation (green rectangles of Figure 5). Moreover, Table 3 provides additional details of the different data types, the software we used to analyze the data, the outputs, and how those are integrated into the analysis workflow.

We built a network of interactions involving intracellular relationships and cytokine mediated intercellular relations that combine published models of different cell types relevant to PDAC, namely, (epithelial) cancer cells, stellate cells, $CD4^+$ T cells, $CD8^+$ T cells, and macrophages. The set of Boolean networks for each cell is provided in the Supporting material, Tables S3-S7. Further, we used cellular deconvolution techniques to estimate cell fractions for each TCGA sample to be used in our model instantiation (see Methods for details of the deconvolution methods). For each sample, DNA sequencing data was used to determine the presence or absence of mutations in KRAS, TP53, CDKN2A, or SMAD4. If a mutation in one of the four genes (g) is absent in a sample then $\alpha_g = 0$, otherwise α_g is calibrated by simulated annealing, as described in the Methods section. Although data from histology images can be used to get estimates of the density of cancer clusters [46], these data are not available in TCGA for PDAC samples.

**Input**	**Method**	**Output**	**Usage**
Gene expression from RNA-seq data	ADAPTS[54]	Cellular composition for each sample	Instantiate the proportions of cell types for each model sample

Gene expression from RNA-seq data	DeMix[56]	Gene expression of cancer cells	Used in the parameter optimization process to find errors between simulations and data
Gene expression from RNA-seq data	ssGSEA[66]	Proliferation and apoptosis scores for each sample	Evaluate the optimized models. These scores are compared with phenotypic scores from simulations
Somatic mutation calls from DNA sequence data	CGC[67]	The presence/absence of important mutations found in samples	Used in the calibration process to determine the set of mutation parameters that will be optimized

Table 3: List of molecular data (Inputs), methods, and descriptions of how the data is integrated into the modeling framework and the analysis pipeline.

Model parameters that cannot be directly estimated from TCGA data are listed in the Supporting material, Table S1. These include rates of cytokine secretion by cancer cells and other cell types, spatial distribution of cancer cells, and receptor activation thresholds. These parameters are calibrated by an optimization process that aims to find an optimum parameter set (θ^*) to maximize the Spearman correlation between the deconvolved gene expression of cancer cells obtained from TCGA and simulations of the framework; Figure S2 of the Supporting material shows a diagram of the optimization protocol; more details of the optimization process can be found in the Parameter Calibration section above. The optimum parameters (θ^*) together with parameters estimated directly from TCGA samples represent personalized models for each TCGA patient sample. Figure 4 shows the histograms of the correlation coefficient of the optimal parameter set compared to random parameters. On average, the correlation coefficient of optimum models over TCGA samples is 0.26, considerably higher than random parameter models, which had an average correlation coefficient of 0.04. Although on average, 0.26 can be improved, there are some samples with correlation coefficient closer to 0.5. By adding more data such as histology images and more detailed models of gene regulation and cell communication, we expect that the accuracy can be further improved. For validating these personalized models, we used gene set scores that can be computed from TCGA gene expression data, using ssGSEA,

which is part of the GSVA R package [66]. The Spearman correlation between the fraction of cancer cells in the proliferation state and the proliferation gene set scores from TCGA samples was 0.17, while the correlation between the fraction of cells in the apoptosis state and the apoptosis gene set scores was 0.2.

Characterizing TCGA subtypes with model parameters

We investigated whether the model parameters, calibrated on TCGA samples, were associated with the previously described subtypes of PDAC. If so, this may reveal an aspect of the model that is more important in particular subtypes, possibly leading to mechanistic hypotheses. Specifically, we measured the difference in parameter values using ANOVA followed by Tukey's Honest Statistical Difference. The association of model parameters (Supporting material, Table S1) was performed using the four subtypes discovered by Bailey et al. [68] (Squamous, Immunogenic, Progenitor, and ADEX) and two from Moffitt et al. [69] (Basal and Classical subtypes).

Our results (Figure 7) showed that among the model parameters, both the probability of KRAS mutation (□$_{KRAS}$, ANOVA p-value=0.013) and the secretion rate of EGF from cancer cells (R_{EGF}^{PCC}, ANOVA p-value=0.038) were associated with Bailey subtypes (Figure 7A). Also, for Moffitt et al. [69] subtypes (Figure 7B) associations were found with probability of TP53 mutation (□$_{TP53}$, p-value=0.01), and EGF secretion rate (R_{EGF}^{PCC}, p-value=0.009). Probability of KRAS mutation was not significantly associated with the Moffitt subtypes (p-value=0.08). It is worth noting that these results and the results of the PCC and PSC interactions (Table 2) reinforce the notion that the EGF autocrine loop plays an important role in PDAC.

Exploration of therapeutic interventions

After the process of parameter calibration and validation, the personalized models can be used to explore the effect of molecular perturbations. A molecular perturbation of a gene is modeled by forcing the state of the gene (a node k in the Boolean network on cell type T) to 0 to model gene repression, or to 1, to model gene overexpression on a fraction (α_k^T) of the cells in the model. By increasing α_k^T, we model the strength of the potential therapeutic intervention.

To do this, we performed simulations with different values of α_k^T and computed Spearman correlation coefficients between the values of α_k^T and the apoptosis state of cancer cells to determine if the perturbation would have an effect. Figure 8A shows the histogram of correlation coefficients between perturbation fractions and apoptosis scores across TCGA samples, focusing on perturbations of bFGF and VEGF nodes in stellate cells. On average perturbing VEGF secretion of stellate cells had a small but negligible impact on cancer apoptosis (average correlation of 0.01). On the other hand, perturbing bFGF had on average a slightly positive impact on cancer apoptosis, with an average of 0.05 across all TCGA samples. It is worth noting that although the estimated effect of bFGF perturbation on apoptosis is small, there are samples with significant positive correlation between perturbation in bFGF in stellate cells and apoptosis of cancer cells. With the null hypothesis that the slope between perturbation fractions and cancer apoptosis is zero, we computed p-values, and found several samples with p-values smaller than 0.05, and some examples with p-values considerably smaller than 0.05 (Figure 8B).

Figures 8C and 8D, comparing two TCGA samples, show correlation plots between apoptosis scores for different fractions of perturbed cells, clearly showing the positive trend of apoptosis induced by perturbation in bGFG, in contrast to the perturbation of VEGF. These results show that TCGA PDAC samples have a heterogeneous response to a perturbation in bFGF cytokine secretion, accounting for the rather weak overall correlation across all samples. Using the model, we can speculate that perturbing the secretion of bFGF by stellate cells could increase cancer cell apoptosis rates for some patients.

Discussion

It is becoming increasingly evident that interactions between cancer cells and the tumor microenvironment (TME) are closely linked to patient outcomes. In this work, we developed a multicellular modeling framework designed to study the molecular interactions between cancer cells and the TME, including stromal and immune cells. This allows model-driven hypotheses to be generated regarding therapeutically relevant PDAC states with potential molecular and cellular drivers, indicating specific potential intervention strategies for further analysis.

The focus of this work is to study how cancer cell states are affected by cell-cell communication within the tumor microenvironment. Only the components of the tumor microenvironment necessary to determine cellular states and intercellular signaling are considered, including gene regulation, spatial distribution of cells, cytokine diffusion, and cell type proportions; other interactions that play a role in tumor growth such as oxygen uptake, mechanical interactions, cell migration, etc. are not included. Our motivation was to generate multicellular models of cancer with a tractable number of parameters that permits the validation and instantiation of the model with omics data, and efficient parameter exploration. Importantly, many of the model parameters can be directly estimated from omics and imaging data.

Our modeling framework can incorporate intracellular interactions by implementing Boolean networks for each cell type of the TME as well as cell-cell communication by modeling the diffusion of cytokines secreted by the cells in the TME. Moreover, each cell is determined by its spatial position and the state of its corresponding Boolean network. The molecular interactions can be obtained from previous studies that use gene networks to study cell behaviors relevant to the TME. Public datasets of molecular interactions can further facilitate model creation and expansion [70,71]. Thus, the BNs represent current knowledge about gene regulation of cell behavior. The BNs are not further optimized with experimental data, although BN optimization is a future venue worth exploring.

Given the specific features of the modeling approach, it is worth discussing the implication of the model assumptions. The main assumption is that, with the time scales considered by the model (hours), population changes induced by proliferation, migration, etc., will not substantially affect the interplay between gene regulation and cell signaling. This implies that the phenotypic estimates generated by model simulations represent instantaneous properties of a sample; extensions need to be added to the model for longer time scales. Another important assumption is that the gene expression data used for parameter calibration is assumed to represent a steady state regime of cellular behavior. This assumption is imposed by the nature of the data used for calibration and validation, which is static, it represents a single time point in the cancer dynamics. The consequences of this assumption can be evaluated using high-throughput data at multiple time points which are currently not available.

Using ensemble simulations over random model parameters, one can investigate the degree of association between potential molecular interactions and important multicellular properties, such as tumor survival or degrees of apoptosis. We have used that strategy on a previously developed two cell model of pancreatic cancer. The model consists of interactions between pancreatic cancer cells and stellate cells, connected by inter-cellular interactions mediated by cytokines. Our results show that the EGF mediated autocrine loop in cancer cells is a potential player in the interactions between stellate and cancer cells. When the EGF autocrine loop is partially repressed, increases in the stellate cell population lead to increases in the proliferation of cancer cells. Moreover, the spatial clustering of cancer cells can affect the expression of important gene expression, such as the expression of the EGF receptor. The last result highlights one of the key components of this modeling framework, namely, the ability to study the influence of spatial cellular properties on the tumor phenotype. A more detailed analysis of the role of the spatial distribution of cells on cancer behavior will require further extension of the model since, for simplicity, we assumed that the stromal cells are uniformly distributed in space and that signal degradation is independent of spatial organization of cell.

The molecular scale of the computational framework permits the integration of molecular data from high-throughput omics technologies, such as gene expression and sequencing data. We have developed methods for data integration that allow for the construction of personalized models of PDAC samples. Specifically, gene expression was used to estimate the relative fractions of the cell types included in the models while sequencing data was used to estimate the percentage of cells with mutations in relevant genes. Additionally, tissue histology images could potentially be integrated in the model framework using methods, such as those described in [72]. Images could be used to estimate parameters of spatial properties of tissue samples and improve model instantiation. We have used knowledge of point processes to generate the positions of cancer cells with a user specified parameter of cancer cell clustering. Recently, it was demonstrated that this parameter can be estimated from histological images [46]. This could lead to complex point processes able to generate more realistic spatial arrangements of cancer or stromal and immune cells.

We built a network of interactions by combining published models of different cell types relevant to PDAC, namely, stellate cells, $CD4^{+}$ T cells, $CD8^{+}$ T cells, and macrophages. Additional Boolean network models can be added to the framework in a straightforward manner. Using this five-cell-type model, we found that KRAS mutations and the secretion rate of EGF from cancer cells were associated with Bailey subtypes while TP53 mutations and EGF secretion rate were associated with the Moffitt subtypes, indicating their potential clinical significance.

In addition to cellular BNs, the modeling framework requires parameters related to cell-cell communication and spatial organization of cells. Some of the parameters can be estimated from molecular data; but for the estimation and calibration of the rest of the parameters (Supporting material, Table S1), we proposed an optimization procedure that minimizes the difference in gene expression obtained by simulations and those observed in deconvolved samples from TCGA. Using the expression of other cell types can also be used in the procedure, but that would require more involved deconvolution techniques or perhaps single cell RNA-seq.

Our optimization procedure is based on simulated annealing; but other optimization methods suitable for discrete stochastic dynamics can also be implemented [73]. In particular, recent parameter exploration methods based on machine learning techniques applied to agent based modeling have the potential to generate new and more robust conclusions regarding the influence of cell-cell communication on cancer behavior [74,75].

The estimation and calibration of the model parameters by using data available in TCGA generates personalized models that are characterized by unique model parameter sets. The generated sample-level models have an average correlation coefficient of 0.26 between simulated and TCGA-based cancer gene expression, with some samples reaching values of 0.5. We also compute gene set scores of proliferation and apoptosis for each TCGA sample and use these values to assess the personalized models. Overall, the correlation coefficient between gene set scores of apoptosis and proliferation and the fraction of cells in apoptosis and proliferation states obtained from the model simulations are 0.17 and 0.2, respectively. Although these correlation coefficients are relatively low, they are much better than random parameter sets, and are expected to improve progressively with the addition of more data, such as imaging data, as well as with more detailed models of gene regulation and cell-cell communication. However, it is worth considering that more detailed models typically require more unknown parameters which, in the absence of pertinent data, can compromise the model validation process and parameter exploration. Since the proposed model already includes spatial distributions of cells, we anticipate that the integration of images into the proposed model will not substantially increase the model complexity (number of parameters).

The calibrated model parameters can provide additional knowledge about the PDAC samples that cannot readily be obtained by pure data analysis. We have shown that the model parameters are associated with known disease subtypes defined by two different studies [68,69]. This framework also allows researchers to model the effect of potential molecular perturbations, generating hypotheses to be tested using more comprehensive models and analysis, and

subsequent experimental setups.

Availability of source code and requirements

Project name: Multicellular Boolean Networks

Project home page: https://github.com/boaguilar/multicell_boolean_networks

Code Ocean reproducible capsule: https://doi.org/10.24433/CO.2337238.v1

Operating system(s): Linux

Programming language: C++ and Python

Other requirements: The code requires Biocellion1.2 and Threading Building Blocks library, both free for academic use. We included both dependencies in the repository, so the code is self-contained and ready to be compiled and executed.

License: The MIT License

Availability of supporting data

Snapshots of our code and other supporting data are openly available in the *GigaScience* repository, GigaDB [77].

Additional files

Additional file 1: Supporting material of the manuscript.

Additional file 2: Signature matrix including pancreatic cells for the estimation of cell fractions.

Additional file 3: Barcodes and cellular fractions for each TCGA sample of PDAC.

Additional file 4: Presence (1) or absence (0) of mutation in TP53, CDKN2A, SMAD4, or KRAS for each TCGA sample of PDAC.

Additional file 5: Gene expression of cancer cells obtained by DeMix [56].

Abbreviations

ABM: Agent based modeling; BN: Boolean networks; LHS: Latin hypercube sampling; PDAC: Pancreatic ductal adenocarcinoma; PCC: Pancreatic cancer cells; PRCC: Partial ranked correlation coefficients; PSC: Pancreatic stellate cells; SA: Simulated annealing; TAM: Tumor associated macrophages; TCGA: The cancer genome Atlas; TME: Tumor microenvironment.

Competing of interest

B.A., D.L.G., and I.S. declare no competing interests.

D.L.R., M.M., S.A.D., A.D., M.T., D.B. and A.R.: Bristol-Myers Squibb: Employment, Equity Ownership.

A.D.: Twinstrand Biosciences: Equity Ownership; Bristol-Myers Squibb: Employment, Equity Ownership.

R.H.: Adaptive Biotechnologies: Membership on an entity's Board of Directors or advisory committees; Fraizer Healthcare Partners: Consultancy; NanoString Technologies: Membership on an entity's Board of Directors or advisory committees; Silverback Therapeutics: Membership on an entity's Board of Directors; Bristol-Myers Squibb: Employment, Equity Ownership.

Funding

This study was funded by Celgene Corporation through a Sponsored Research Agreement between Celgene Corporation and the Institute for Systems Biology.

Author's contributions

I.S. and A.V.R. conceived the study; B.A., D.L.G., A.V.R., and I.S. designed the research; R.H., A.D., M.T., and D.B. provided feedback on the research design; B.A., D.L.G., D.L.R., A.D., R.H., A.V.R, and I.S.: conceptualization; B.A. and D.L.G.: investigation and formal analysis; B.A., D.L.G., D.L.R., M.M., S.A.D., A.V.R., and I.S.: methodology design; M.T., D.B., R.H., A.V.R., and I.S.: project administration and supervision; B.A. and D.L.G. wrote the manuscript; I.S. and A.V.R. revised the manuscript. All authors read and approved the final draft.

Acknowledgements

The authors thank Wenyi Wang for kindly providing gene expression of cancer cells in TCGA samples obtained by DeMix. The authors thank Alessandro Palma for kindly providing the Boolean network of macrophages. We also thank William Longabaugh for creating the initial BioTapestry network used in Figure 3.

List of Figures

Figure 1. Schematic representation of the multiscale model including multiple cell types and cytokines of the TME.

Figure 2. A. Top views of 3D spatial configuration of a two cell model; stellate cells are in grey while cancer cells are in red and blue; red for cancer cells with proliferation nodes in ON state and blue for cancer cells with proliferation nodes in OFF state, for cancer cells we used $s = 0.07$. The top panel shows the spatial configuration at the beginning of a simulation and the low panel shows the configuration after 100 time steps. **B.** The average proportion of cancer cells with active

proliferation (red solid line) and apoptosis (black solid line) as a function of time steps. Averages and standard deviations were computed from 10 simulations. More details about simulation parameters can be found in the Supporting material, Table S2.

Figure 3. Network of molecular interactions in pancreatic cancer cells (A, Green area) and pancreatic stellate cells (B, Yellow area). Extracellular cytokines between these two cells are in the orange area. Adapted from Wang et al. [48] and illustrated in Biotapestry [76]. The Boolean functions for each gene of the two cells are available in the Supporting material, Tables S3 and S4.

Figure 4. Association of Model Parameters (Columns) with cancer cell phenotypes (Rows). Color scale shows Partial Rank Correlation Coefficient (PRCC) obtained from simulations of 1000 random parameters.

Figure 5. Diagram of the data-driven computational framework to instantiate, calibrate, validate and explore patient-specific multiscale models of the TME to generate actionable and therapeutically relevant hypotheses.

Figure 6. Histogram of correlation coefficient between gene expression obtained from simulations and those from DeMix expression deconvolution. Blue Bars are the best correlation coefficient obtained testing an ensemble of random parameters. The Grey bars are the correlation coefficient from a random set of parameters.

Figure 7. A. PCC secretion rate of EGF parameter values within each subtype defined by Bailey et al. [68] squamous (1), immunogenic (2), progenitor (3), and ADEX (4). **B.** PCC secretion rate of EGF parameter values within each subtype defined by Moffitt et al. [69], basal (1) and classical

(2). We used 119 samples of PDAC available in TCGA; the barcode identifiers of these samples are available in the Supporting material, Additional file 3.

Figure 8. Effects of gene perturbation in stellate cells on apoptosis states in cancer cells. **A.** Distribution of correlation coefficients between apoptosis scores and the percentage of perturbation in bFGF (blue) and VEGF (Red) in stellate cells, over 119 TCGA samples of PDAC (Additional file 3). **B.** The slope of the linear fit between apoptosis scores and the percentage of perturbed stellate cells, versus the p-value of the hypothesis that the slope is zero. The red (blue) circles represent samples with a perturbation in bFGF (VEGF) and the dashed vertical line represents a p-value = 0.05. **C.** Average apoptosis scores for cancer cells within one sample as a function of the percentage of perturbations of bFGF (blue) and VEGF (red) for the two samples with the largest correlation coefficient; error bars represent standard deviations. Averages and standard deviations were computed from 15 simulations performed with a constant percentage of perturbed cells.

References:

1. Rahib L, Smith BD, Aizenberg R, Rosenzweig AB, Fleshman JM, Matrisian LM. Projecting cancer incidence and deaths to 2030: the unexpected burden of thyroid, liver, and pancreas cancers in the United States. Cancer Res. 2014;74: 2913–2921.

2. Gore J, Korc M. Pancreatic Cancer Stroma: Friend or Foe? Cancer Cell. 2014. pp. 711–712. doi:10.1016/j.ccr.2014.05.026

3. Baker RE, Peña J-M, Jayamohan J, Jérusalem A. Mechanistic models versus machine learning, a fight worth fighting for the biological community? Biol Lett. 2018;14. doi:10.1098/rsbl.2017.0660

4. Huang S. The Tension Between Big Data and Theory in the “Omics” Era of Biomedical Research. Perspect Biol Med. 2018;61: 472–488.

5. Mast FD, Ratushny AV, Aitchison JD. Systems cell biology. The Journal of Cell Biology.

2014. pp. 695–706. doi:10.1083/jcb.201405027

6. Palma A, Jarrah AS, Tieri P, Cesareni G, Castiglione F. Gene Regulatory Network Modeling of Macrophage Differentiation Corroborates the Continuum Hypothesis of Polarization States. Front Physiol. 2018;9: 1659.

7. Rex J, Albrecht U, Ehlting C, Thomas M, Zanger UM, Sawodny O, et al. Model-Based Characterization of Inflammatory Gene Expression Patterns of Activated Macrophages. PLoS Comput Biol. 2016;12: e1005018.

8. Castiglione F, Tieri P, Palma A, Jarrah AS. Statistical ensemble of gene regulatory networks of macrophage differentiation. BMC Bioinformatics. 2016;17: 506.

9. Bolouri H, Young M, Beilke J, Johnson R, Fox B, Huang L, et al. Integrative network modeling reveals mechanisms underlying T cell exhaustion. Scientific Reports. 2020;10: 1915.

10. Mendoza L, Xenarios I. A method for the generation of standardized qualitative dynamical systems of regulatory networks. Theor Biol Med Model. 2006;3: 13.

11. Tieri P, Prana V, Colombo T, Santoni D, Castiglione F. Multi-scale Simulation of T Helper Lymphocyte Differentiation. Advances in Bioinformatics and Computational Biology. 2014. pp. 123–134. doi:10.1007/978-3-319-12418-6_16

12. Li F, Long T, Lu Y, Ouyang Q, Tang C. The yeast cell-cycle network is robustly designed. Proc Natl Acad Sci U S A. 2004;101: 4781–4786.

13. Tyson JJ. Modeling the cell division cycle: cdc2 and cyclin interactions. Proc Natl Acad Sci U S A. 1991;88: 7328–7332.

14. Novák B, Tyson JJ. A model for restriction point control of the mammalian cell cycle. J Theor Biol. 2004;230: 563–579.

15. Choi M, Shi J, Jung SH, Chen X, Cho K-H. Attractor landscape analysis reveals feedback loops in the p53 network that control the cellular response to DNA damage. Sci Signal. 2012;5: ra83.

16. Kather JN, Poleszczuk J, Suarez-Carmona M, Krisam J, Charoentong P, Valous NA, et al. Modeling of Immunotherapy and Stroma-Targeting Therapies in Human Colorectal Cancer. Cancer Res. 2017;77: 6442–6452.

17. Ghaffarizadeh A, Heiland R, Friedman SH, Mumenthaler SM, Macklin P. PhysiCell: An open source physics-based cell simulator for 3-D multicellular systems. PLoS Comput Biol. 2018;14: e1005991.

18. Gong C, Milberg O, Wang B, Vicini P, Narwal R, Roskos L, et al. A computational multiscale agent-based model for simulating spatio-temporal tumour immune response to PD1 and PDL1 inhibition. J R Soc Interface. 2017;14. doi:10.1098/rsif.2017.0320

19. Wells DK, Chuang Y, Knapp LM, Brockmann D, Kath WL, Leonard JN. Spatial and Functional Heterogeneities Shape Collective Behavior of Tumor-Immune Networks. PLOS Computational Biology. 2015. p. e1004181. doi:10.1371/journal.pcbi.1004181

20. Norton K-A, Gong C, Jamalian S, Popel AS. Multiscale Agent-Based and Hybrid Modeling of the Tumor Immune Microenvironment. Processes (Basel). 2019;7. doi:10.3390/pr7010037

21. Gatenby RA, Smallbone K, Maini PK, Rose F, Averill J, Nagle RB, et al. Cellular adaptations to hypoxia and acidosis during somatic evolution of breast cancer. British Journal of Cancer. 2007. pp. 646–653. doi:10.1038/sj.bjc.6603922

22. Smallbone K, Gatenby RA, Gillies RJ, Maini PK, Gavaghan DJ. Metabolic changes during carcinogenesis: Potential impact on invasiveness. Journal of Theoretical Biology. 2007. pp. 703–713. doi:10.1016/j.jtbi.2006.09.010

23. Spill F, Guerrero P, Alarcon T, Maini PK, Byrne HM. Mesoscopic and continuum modelling of angiogenesis. J Math Biol. 2015;70: 485–532.

24. McDougall SR, Anderson ARA, Chaplain MAJ. Mathematical modelling of dynamic adaptive tumour-induced angiogenesis: clinical implications and therapeutic targeting strategies. J Theor Biol. 2006;241: 564–589.

25. Reher D, Klink B, Deutsch A, Voss-Böhme A. Cell adhesion heterogeneity reinforces tumour cell dissemination: novel insights from a mathematical model. Biol Direct. 2017;12: 18.

26. Rejniak KA, Wang SE, Bryce NS, Chang H, Parvin B, Jourquin J, et al. Linking changes in epithelial morphogenesis to cancer mutations using computational modeling. PLoS Comput Biol. 2010;6. doi:10.1371/journal.pcbi.1000900

27. Metzcar J, Wang Y, Heiland R, Macklin P. A Review of Cell-Based Computational Modeling in Cancer Biology. JCO Clin Cancer Inform. 2019;3: 1–13.

28. Macklin P. Key challenges facing data-driven multicellular systems biology. GigaScience. 2019; 8: 1–8.

29. Yankeelov TE, Quaranta V, Evans KJ, Rericha EC. Toward a Science of Tumor Forecasting for Clinical Oncology. Cancer Research. 2015. pp. 918–923. doi:10.1158/0008-5472.can-14-2233

30. Hutchinson L, Steiert B, Soubret A, Wagg J, Phipps A, Peck R, et al. Models and Machines: How Deep Learning Will Take Clinical Pharmacology to the Next Level. CPT Pharmacometrics Syst Pharmacol. 2019;8: 131–134.

31. Shmulevich I, Dougherty ER. Probabilistic Boolean Networks: The Modeling and Control of Gene Regulatory Networks. SIAM; 2010.

32. Voukantsis D, Kahn K, Hadley M, Wilson R, Buffa FM. Modeling genotypes in their microenvironment to predict single- and multi-cellular behavior. Gigascience. 2019;8. doi:10.1093/gigascience/giz010

33. Letort G, Montagud A, Stoll G, Heiland R, Barillot E, Macklin P, et al. PhysiBoSS: a multi-scale agent-based modelling framework integrating physical dimension and cell signalling. Bioinformatics. 2019;35: 1188–1196.

34. Stoll G, Caron B, Viara E, Dugourd A, Zinovyev A, Naldi A, et al. MaBoSS 2.0: an

environment for stochastic Boolean modeling. Bioinformatics. 2017;33: 2226–2228.

35. Stoll G, Viara E, Barillot E, Calzone L. Continuous time boolean modeling for biological signaling: application of Gillespie algorithm. BMC Systems Biology. 2012. p. 116. doi:10.1186/1752-0509-6-116

36. Dougherty ER. The Evolution of Scientific Knowledge: From Certainty to Uncertainty Full Book. The Evolution of Scientific Knowledge: From Certainty to Uncertainty. doi:10.1117/3.2263362.sup

37. Vundavilli H, Datta A, Sima C, Hua J, Lopes R, Bittner ML. In Silico Design and Experimental Validation of Combination Therapy for Pancreatic Cancer. IEEE/ACM Trans Comput Biol Bioinform. 2018. doi:10.1109/TCBB.2018.2872573

38. Layek R, Datta A, Bittner M, Dougherty ER. Cancer therapy design based on pathway logic. Bioinformatics. 2011;27: 548–555.

39. Shmulevich I, Dougherty ER, Kim S, Zhang W. Probabilistic Boolean Networks: a rule-based uncertainty model for gene regulatory networks. Bioinformatics. 2002;18: 261–274.

40. Kang C, Aguilar B, Shmulevich I. Emergence of diversity in homogeneous coupled Boolean networks. Physical Review E. 2018. doi:10.1103/physreve.97.052415

41. Shmulevich I, Dougherty ER, Zhang W. Gene perturbation and intervention in probabilistic Boolean networks. Bioinformatics. 2002. pp. 1319–1331. doi:10.1093/bioinformatics/18.10.1319

42. Olimpio EP, Dang Y, Youk H. Statistical Dynamics of Spatial-Order Formation by Communicating Cells. iScience. 2018;2: 27–40.

43. Maire T, Youk H. Molecular-Level Tuning of Cellular Autonomy Controls the Collective Behaviors of Cell Populations. Cell Syst. 2015;1: 349–360.

44. Berg HC. Random Walks in Biology. 2018. doi:10.2307/j.ctv7r40w6

45. Thomas M. A Generalization of Poisson’s Binomial Limit For use in Ecology. Biometrika. 1949. p. 18. doi:10.2307/2332526

46. Jones-Todd CM, Caie P, Illian JB, Stevenson BC, Savage A, Harrison DJ, et al. Identifying prognostic structural features in tissue sections of colon cancer patients using point pattern analysis. Statistics in Medicine. 2019. pp. 1421–1441. doi:10.1002/sim.8046

47. Kang S, Kahan S, McDermott J, Flann N, Shmulevich I. Biocellion: accelerating computer simulation of multicellular biological system models. Bioinformatics. 2014;30: 3101–3108.

48. Wang Q, Miskov-Zivanov N, Liu B, Faeder JR, Lotze M, Clarke EM. Formal Modeling and Analysis of Pancreatic Cancer Microenvironment. Computational Methods in Systems Biology. 2016. pp. 289–305. doi:10.1007/978-3-319-45177-0_18

49. Cancer Genome Atlas Research Network. Electronic address: andrew_aguirre@dfci.harvard.edu, Cancer Genome Atlas Research Network. Integrated Genomic Characterization of Pancreatic Ductal Adenocarcinoma. Cancer Cell. 2017;32: 185–203.e13.

50. Arango Duque G, Descoteaux A. Macrophage cytokines: involvement in immunity and infectious diseases. Front Immunol. 2014;5: 491.

51. Hao N-B, Lü M-H, Fan Y-H, Cao Y-L, Zhang Z-R, Yang S-M. Macrophages in Tumor Microenvironments and the Progression of Tumors. Clinical and Developmental Immunology. 2012. pp. 1–11. doi:10.1155/2012/948098

52. Wherry EJ, John Wherry E, Kurachi M. Molecular and cellular insights into T cell exhaustion. Nature Reviews Immunology. 2015. pp. 486–499. doi:10.1038/nri3862

53. Wherry EJ, John Wherry E. T cell exhaustion. Nature Immunology. 2011. pp. 492–499. doi:10.1038/ni.2035

54. Danziger SA, Gibbs DL, Shmulevich I, McConnell M, Trotter MWB, Schmitz F, et al. ADAPTS: Automated Deconvolution Augmentation of Profiles for Tissue Specific cells. PLoS ONE. 2019;14: e0224693.

55. Ellrott K, Bailey MH, Saksena G, Covington KR, Kandoth C, Stewart C, et al. Scalable Open Science Approach for Mutation Calling of Tumor Exomes Using Multiple Genomic Pipelines. Cell Syst. 2018;6: 271–281.e7.

56. Ahn J, Yuan Y, Parmigiani G, Suraokar MB, Diao L, Wistuba II, et al. DeMix: deconvolution for mixed cancer transcriptomes using raw measured data. Bioinformatics. 2013. pp. 1865–1871. doi:10.1093/bioinformatics/btt301

57. van Laarhoven PJM, Aarts EHL. Performance of the simulated annealing algorithm. Simulated Annealing: Theory and Applications. 1987. pp. 77–98. doi:10.1007/978-94-015-7744-1_6

58. Kirkpatrick S, Gelatt CD Jr, Vecchi MP. Optimization by simulated annealing. Science. 1983;220: 671–680.

59. Baron M, Veres A, Wolock SL, Faust AL, Gaujoux R, Vetere A, et al. A Single-Cell Transcriptomic Map of the Human and Mouse Pancreas Reveals Inter- and Intra-cell Population Structure. Cell Syst. 2016;3: 346–360.e4.

60. Chen B, Khodadoust MS, Liu CL, Newman AM, Alizadeh AA. Profiling Tumor Infiltrating Immune Cells with CIBERSORT. Methods Mol Biol. 2018;1711: 243–259.

61. Vonlaufen A, Joshi S, Qu C, Phillips PA, Xu Z, Parker NR, et al. Pancreatic stellate cells: partners in crime with pancreatic cancer cells. Cancer Res. 2008;68: 2085–2093.

62. Marino S, Hogue IB, Ray CJ, Kirschner DE. A methodology for performing global uncertainty and sensitivity analysis in systems biology. J Theor Biol. 2008;254: 178–196.

63. Erkan M, Michalski CW, Rieder S, Reiser-Erkan C, Abiatari I, Kolb A, et al. The activated stroma index is a novel and independent prognostic marker in pancreatic ductal adenocarcinoma. Clin Gastroenterol Hepatol. 2008;6: 1155–1161.

64. Fujita H, Ohuchida K, Mizumoto K, Nakata K, Yu J, Kayashima T, et al. alpha-Smooth Muscle Actin Expressing Stroma Promotes an Aggressive Tumor Biology in Pancreatic Ductal Adenocarcinoma. Pancreas. 2010;39: 1254–1262.

65. Blaine SA, Ray KC, Branch KM, Robinson PS, Whitehead RH, Means AL. Epidermal growth factor receptor regulates pancreatic fibrosis. Am J Physiol Gastrointest Liver Physiol. 2009;297: G434–41.

66. Hänzelmann S, Castelo R, Guinney J. GSVA: gene set variation analysis for microarray and RNA-seq data. BMC Bioinformatics. 2013;14: 7.

67. Reynolds SM, Miller M, Lee P, Leinonen K, Paquette SM, Rodebaugh Z, et al. The ISB Cancer Genomics Cloud: A Flexible Cloud-Based Platform for Cancer Genomics Research. Cancer Research. 2017. pp. e7–e10. doi:10.1158/0008-5472.can-17-0617

68. Bailey P, Chang DK, Nones K, Johns AL, Patch A-M, Gingras M-C, et al. Genomic analyses identify molecular subtypes of pancreatic cancer. Nature. 2016;531: 47–52.

69. Moffitt RA, Marayati R, Flate EL, Volmar KE, Loeza SGH, Hoadley KA, et al. Virtual microdissection identifies distinct tumor- and stroma-specific subtypes of pancreatic ductal adenocarcinoma. Nat Genet. 2015;47: 1168–1178.

70. Traynard P, Tobalina L, Eduati F, Calzone L, Saez-Rodriguez J. Logic Modeling in Quantitative Systems Pharmacology. CPT Pharmacometrics Syst Pharmacol. 2017;6: 499–511.

71. Perfetto L, Briganti L, Calderone A, Perpetuini AC, Iannuccelli M, Langone F, et al. SIGNOR: a database of causal relationships between biological entities. Nucleic Acids Research. 2016. pp. D548–D554. doi:10.1093/nar/gkv1048

72. Saltz J, Gupta R, Hou L, Kurc T, Singh P, Nguyen V, et al. Spatial Organization and Molecular Correlation of Tumor-Infiltrating Lymphocytes Using Deep Learning on Pathology Images. Cell Rep. 2018;23: 181–193.e7.

73. Rios LM, Sahinidis NV. Derivative-free optimization: a review of algorithms and comparison of software implementations. Journal of Global Optimization. 2013. pp. 1247–1293. doi:10.1007/s10898-012-9951-y

74. Ozik J, Collier N, Wozniak JM, Macal C, Cockrell C, Friedman SH, et al. High-throughput cancer hypothesis testing with an integrated PhysiCell-EMEWS workflow. BMC Bioinformatics. 2018;19: 483.

75. Ozik J, Collier N, Heiland R, An G, Macklin P. Learning-accelerated discovery of immune-tumour interactions. Mol Syst Des Eng. 2019;4: 747–760.

76. Longabaugh WJR. BioTapestry: a tool to visualize the dynamic properties of gene regulatory networks. Methods Mol Biol. 2012;786: 359–394.

77. Aguilar B; Gibbs DL; Reiss DL; McConnell M; Danziger SA; Dervan A; Trotter M; Bassett D; Hershberg R; Ratushny AV; Shmulevich I: Supporting data for "A generalizable data-driven multicellular model of pancreatic ductal adenocarcinoma" GigaScience Database. 2020. http://dx.doi.org/10.5524/100763

Figure1

Click here to access/download;Figure;Figure1.pdf

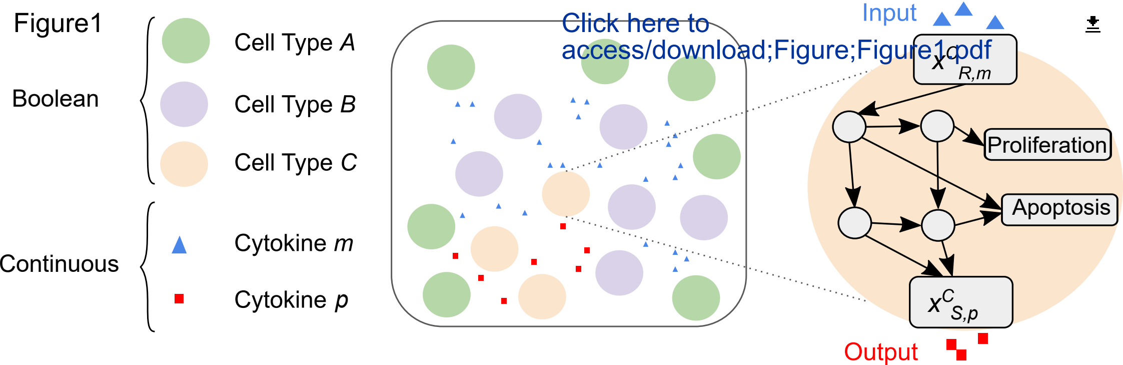

Figure2

Click here to access/download;Figure;Figure2.pdf

A

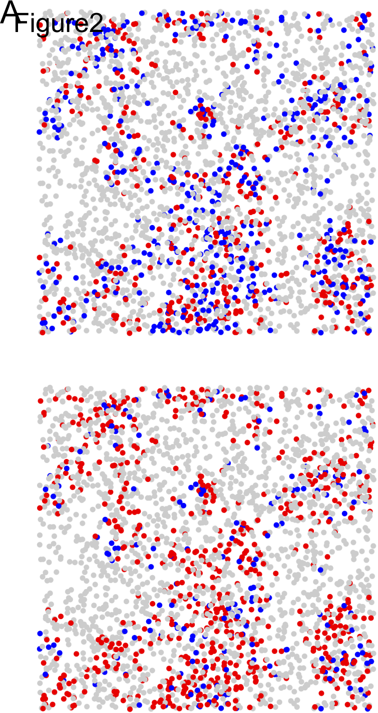

B

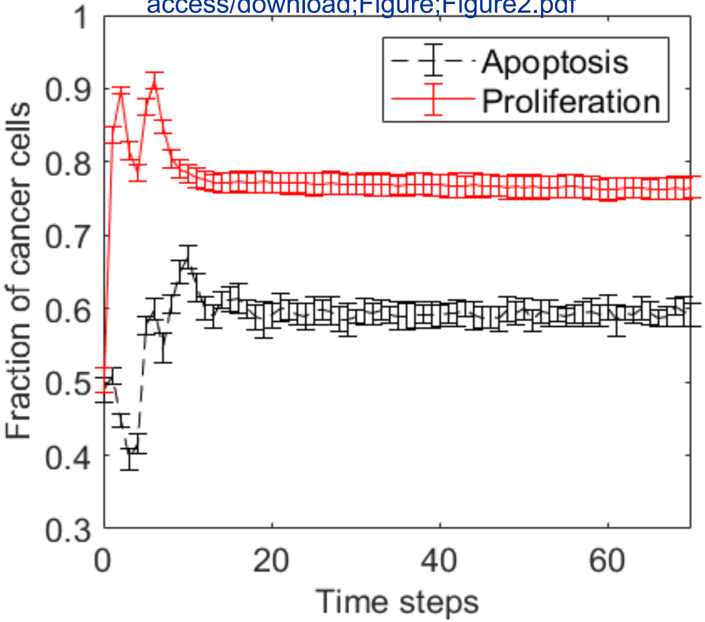

Figure3

Click here to access/download;Figure;Figure3.pdf

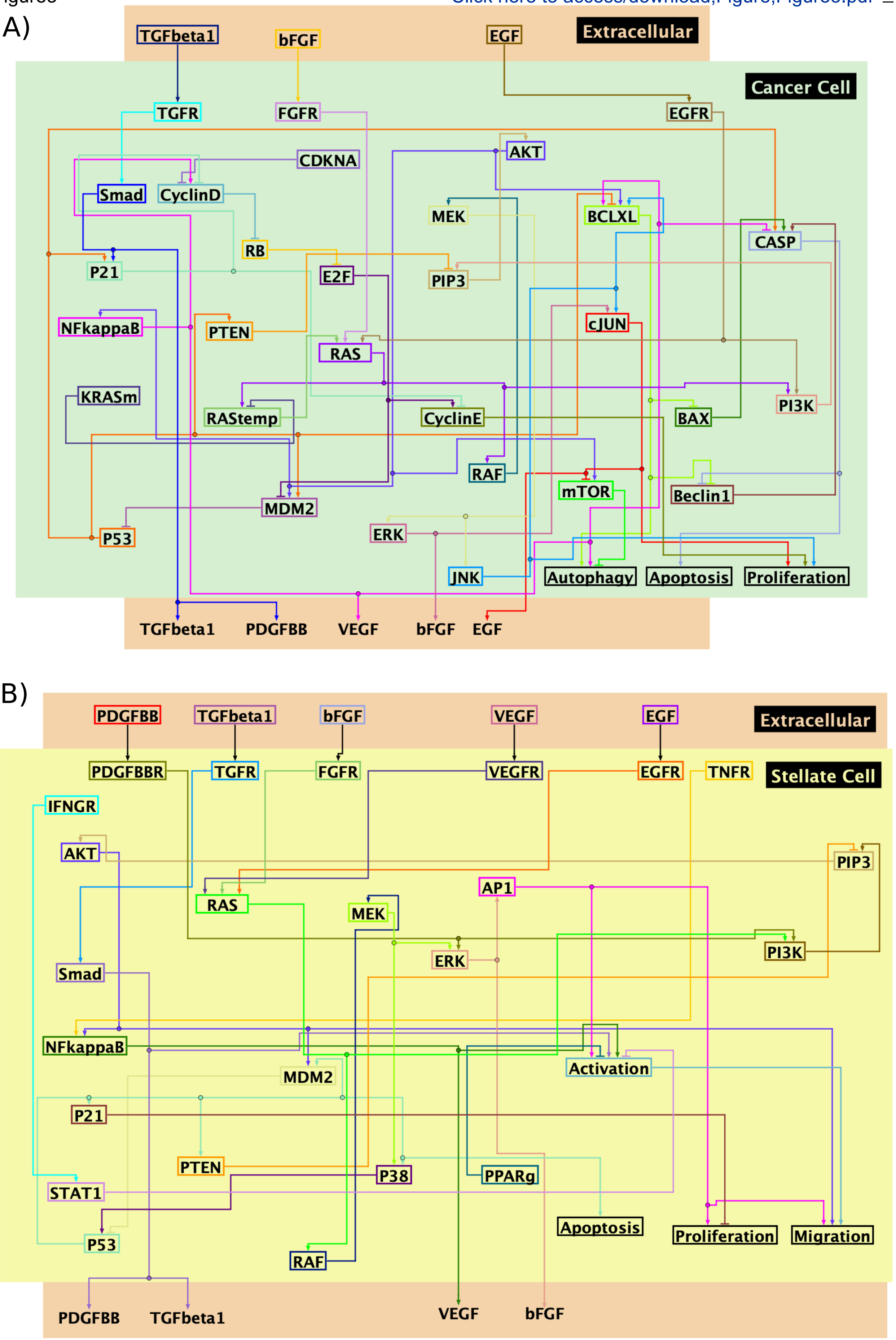

Figure4

Click here to access/download;Figu

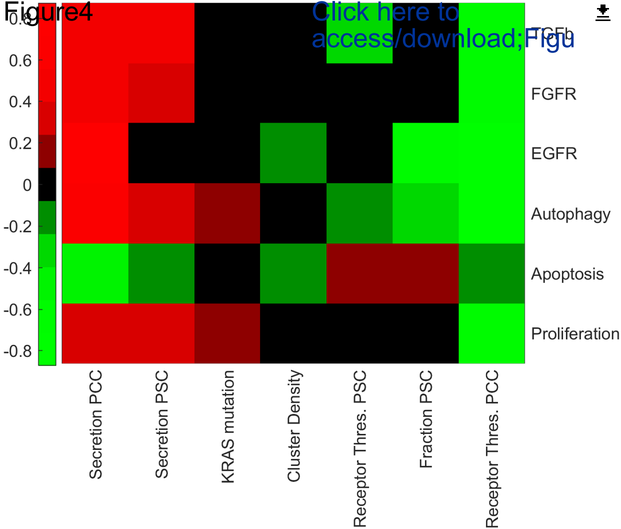

Figure5

Click here to access/download;Figure;Figure5.pdf

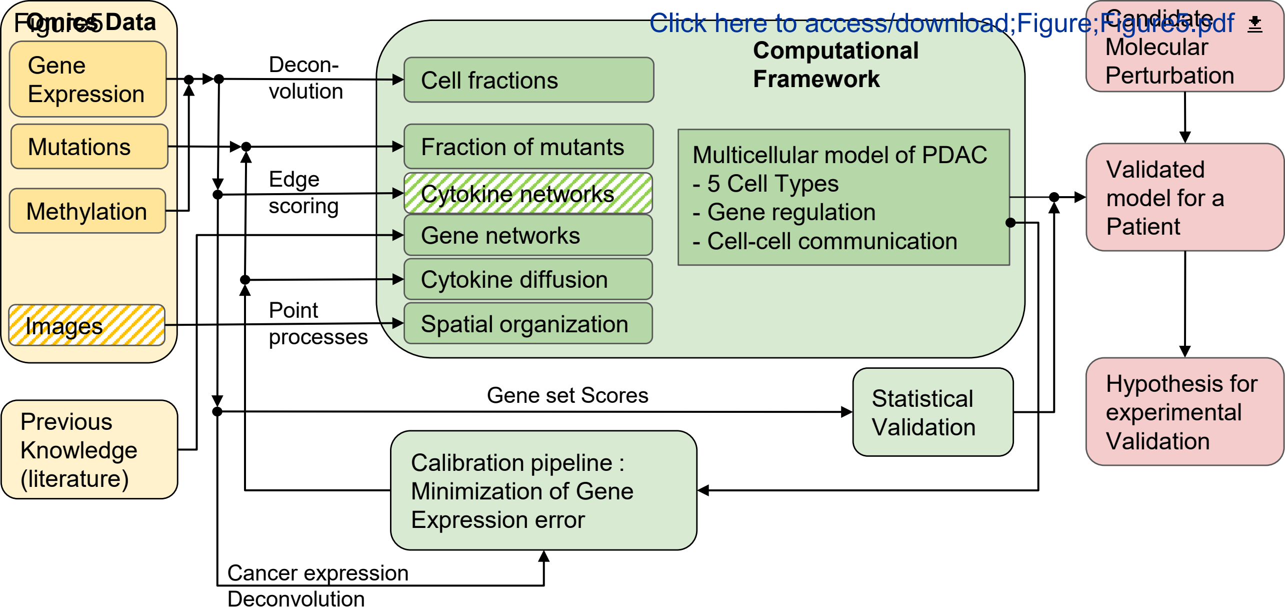

Figure6

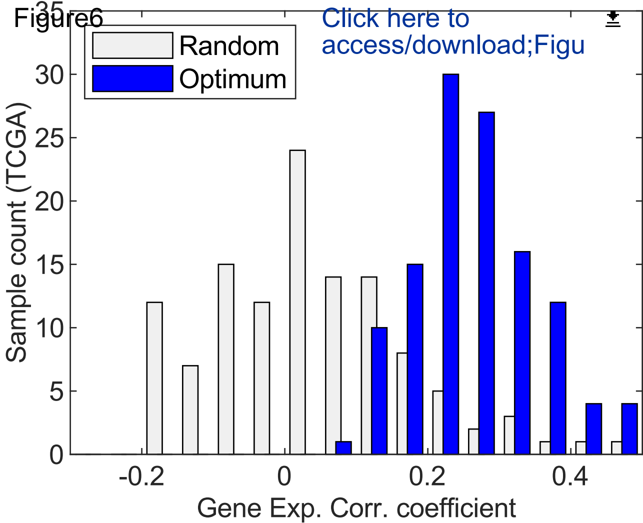

Click here to access/download;Figu

Figure7

Click here to access/download;Figure;Figure7.pdf

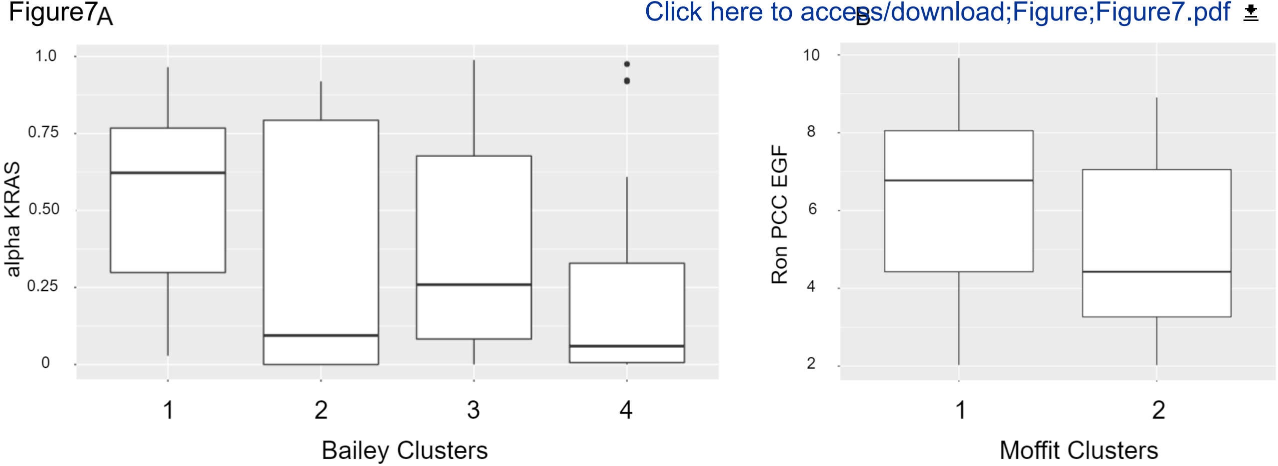

Figure8

Click here to access/download;Figure;Figure8.pdf

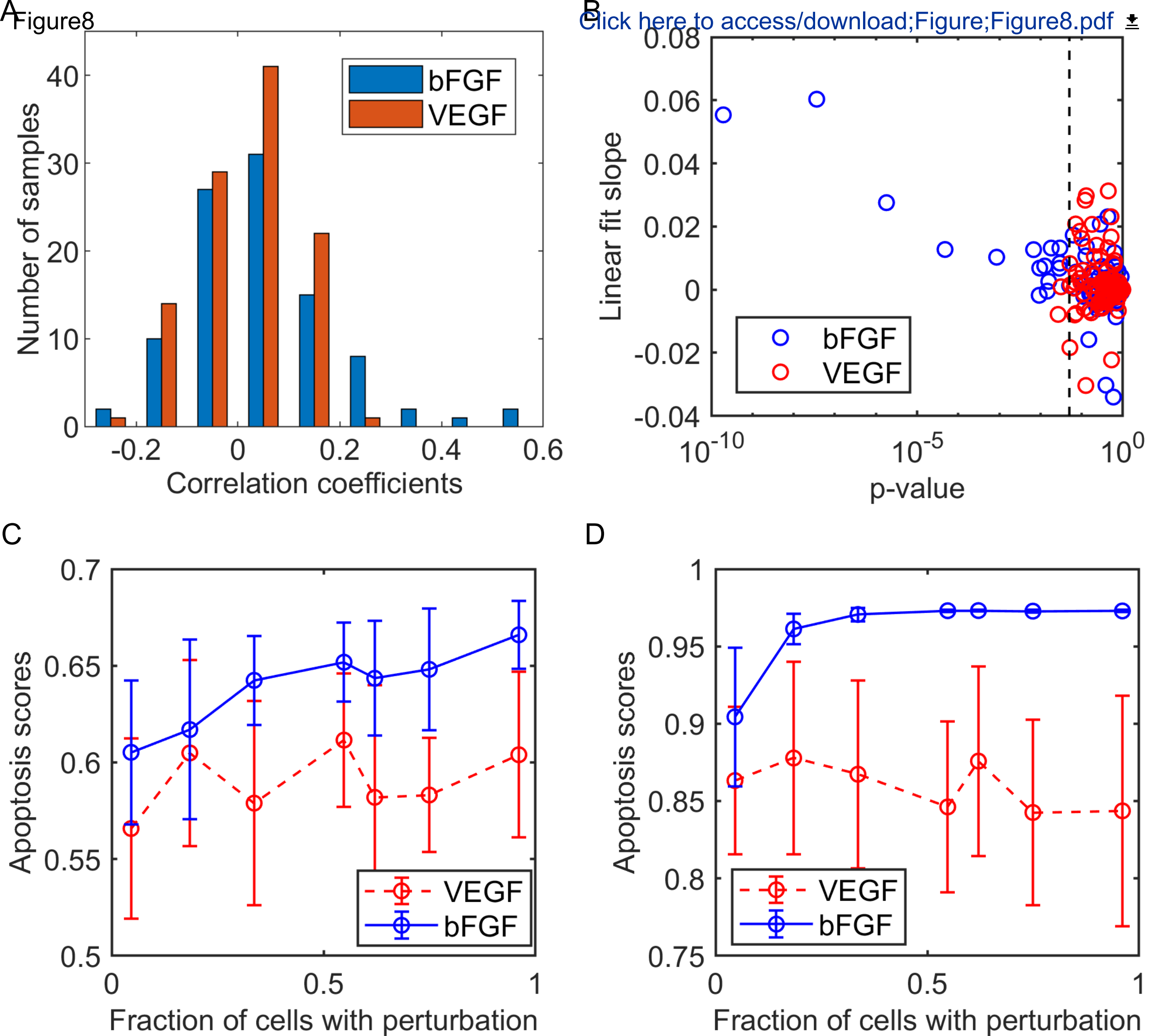

Additional file 1

Click here to access/download

Supplementary Material

Supporting Material PDAC Manuscript.pdf

Additional file 2

Click here to access/download
Supplementary Material
Pancreatic_Cell_Signatures.tsv

Additional file 3

Click here to access/download

Supplementary Material

barcodes_cell_fractions_5cellmodel_tcga.txt

Additional file 4

Click here to access/download
Supplementary Material
MutationStates_TCGA_CancerCells.txt

Additional file 5

Click here to access/download
Supplementary Material
paad_demix_tumor_geneexpression.tsv
