# Supplementary material for: A generalizable data-driven multicellular model of pancreatic ductal adenocarcinoma
Source: Gigascience. 2020 Jul 22;9(7):giaa075. doi: 10.1093/gigascience/giaa075 (PMC7374045; doi:10.1093/gigascience/giaa075)
Supplement: giaa075_Supplemental_Files [file giaa075_supplemental_files.zip › Supporting Material PDAC Manuscript[1].pdf]

Supporting Material: A generalizable data-driven multicellular model of pancreatic ductal adenocarcinoma

Table S1 List of parameters and their corresponding ranges used in the parameter calibration pipeline. The density of cells was set to 0.14 points per volume; the simulation domain was set to 320x320x320 μm^3 ; the effective interaction distance, λ, was set up to 30.

**Parameter name**	**Symbol**	**Range**
Secretion rate of EGF by cancer cells	R_{EGF}^{PCC}	2.0 - 10.0
Secretion rate of VEGF by cancer cells	R_{VEGF}^{PCC}	2.0 - 10.0
Secretion rate of TGFβ by of cancer cells	$R_{TGF\beta}^{PCC}$	2.0 - 10.0
Secretion rate of bFGF by cancer cells	R_{bFGF}^{PCC}	2.0 - 10.0
Secretion rate of PDGFBB by cancer cells	R_{PDGFBB}^{PCC}	2.0 - 10.0
Secretion rate of all cytokines by stellate cells	R^{PSC}	2.0 - 10.0
Secretion rate of all cytokines by CD4 T cells	R^{CD4}	2.0 - 10.0
Secretion rate of all cytokines by macrophages cells	R^{TAM}	2.0 - 10.0
Secretion rate of all cytokines by CD8 T cells	R^{CD8}	2.0 - 10.0
Activation threshold for all the receptors	K	4.0 - 40.0
Density of cancer clusters	s	0.004 - 1.0
Fraction of cancer cells with KRAS mutation	α_{KRAS}	0.01 - 1.0
Fraction of cancer cells with CDKN2A mutation	α_{CDKN2A}	0.01 - 1.0
Fraction of cancer cells with TP53 mutation	α_{TP53}	0.01 - 1.0
Fraction of cancer cells with SMAD4 mutation	α_{SMAD4}	0.01 - 1.0

Table S2 List of parameters used for the Sensitivity Analysis (Figure 3 of the main text). The density of cells was set to 0.14 points per volume; the simulation domain was set to 320x320x320 μm^3 ; the probability of mutations in P53, CDKNA, and SMAD4 are set to zero; and the effective interaction distance, λ, was set to 30.

**Parameter name**	**Symbol**	**Range**
Fraction of stellate cells	r_s	0.1 - 0.9
Secretion rate of cancer cells	R^{PCC}	2.0 - 10.0
Secretion rate of cancer cells	R^{PSC}	2.0 - 10.0
Activation threshold of cancer cells	K^{PSC}	4.0 - 40.0
Activation threshold of stellate cells	K^{PSC}	4.0 - 40.0
Density of cancer clusters	s	0.004 - 1.0
Fraction of cells with KRAS mutation	α_{KRAS}	0.01 - 1.0

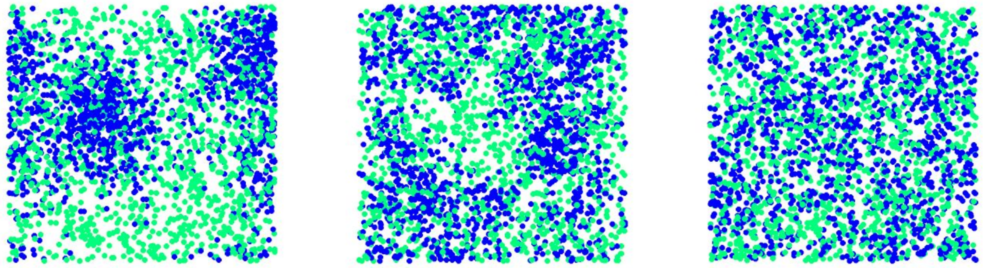

Figure S1. Top view of cellular distributions generated using three values of s (0.004, 0.064, and 1.0 corresponding to the left, middle, and right panel respectively). Cancer cells are in blue while stromal cells in green.

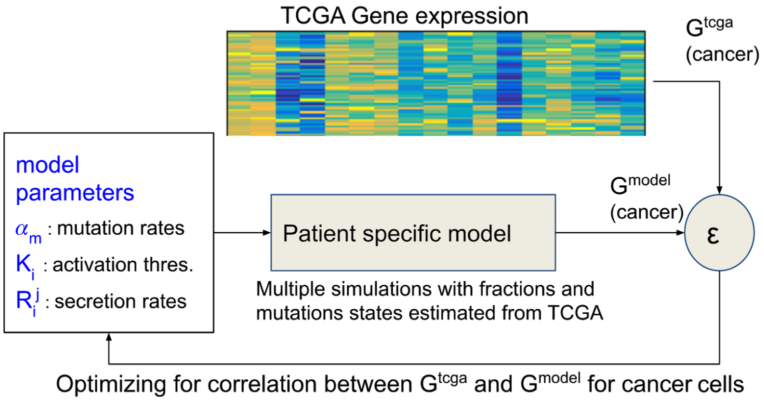

Figure S2. Optimization protocol used to calibrate the model parameters. The optimization process targets gene expression data of cancer cells separately for each patient sample. Gene expression profiles of cancer cells from simulations (G^{model}) are compared with cancer cell expression profiles (G^{tcga}) from TCGA by computing Spearman correlations (ε). G^{tcga} is estimated for each TCGA sample by using the DeMix method (Bioinformatics. 2013 Aug 1;29(15):1865-71) and RNA-seq data available in TCGA.

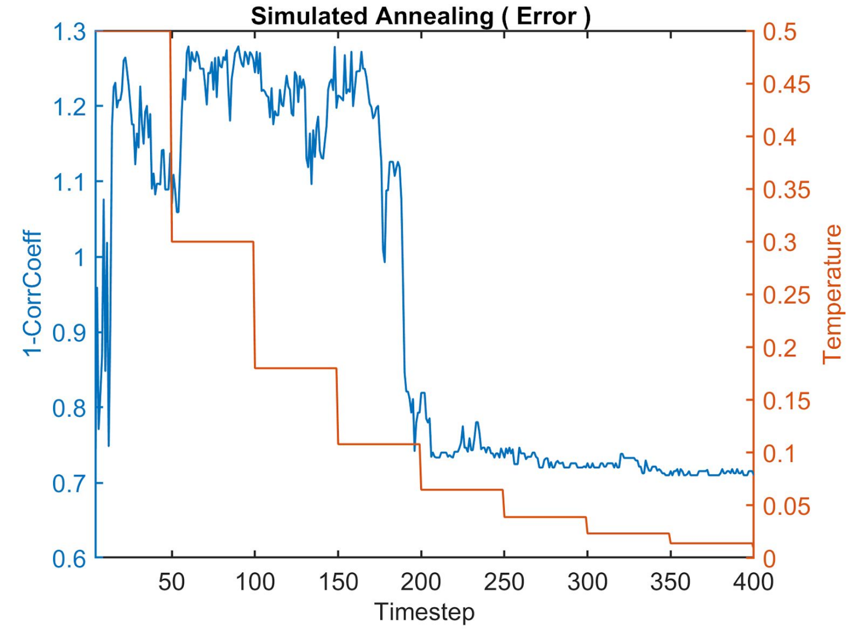

Figure S3 Error (Blue) of the Simulated Annealing process as function of timesteps and temperature (Red). CorrCoeff is the Spearman correlation coefficient between computed and TCGA based gene expression of cancer cells.

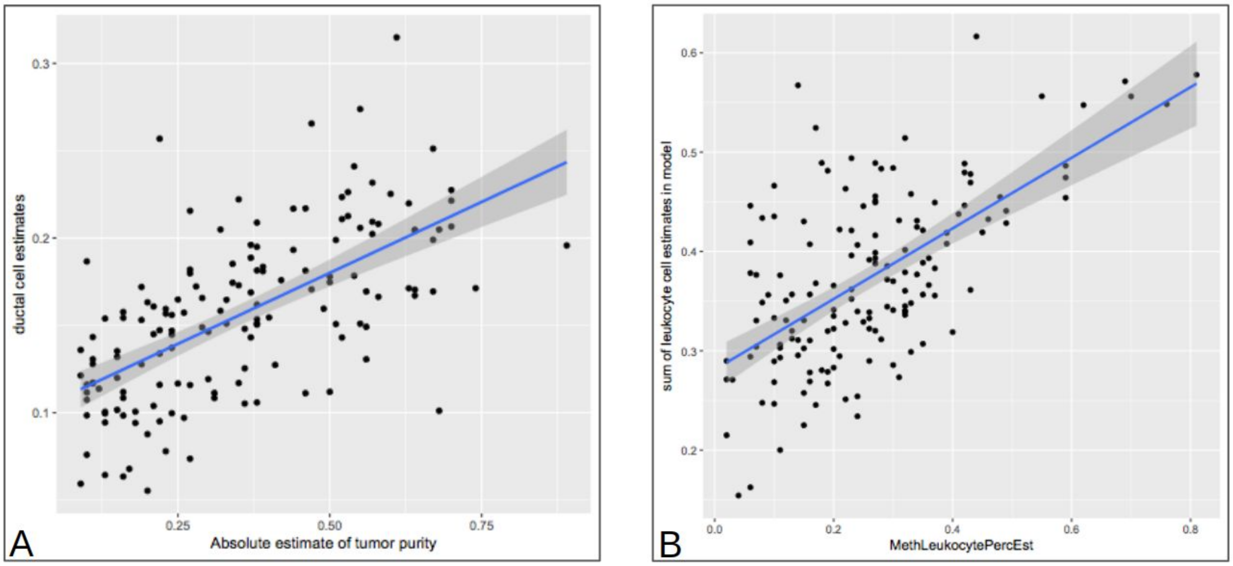

Figure S4. (A) Comparing ductal cell quantities (y-axis) to tumor purity estimates from Absolute (software) which uses copy number variation to estimate tumor purity (x-axis). (B) Comparing a sum of leukocyte estimates (y-axis) to estimates of total leukocyte content which were generated using methylation data and released as part of the TCGA PanCancer Atlas (x-axis).

Table S3. Boolean network of cancer cells

EGFR = EGF_external
FGFR = bFGF _external
TGFR = TGFb_external
TOXICITY = TOXICITY_external
PDL1 = 0
CDKNA = 1
KRASm = 1
PI3K = (EGFR) | (RAS)
RAS = ((RAStemp) | (EGFR) | (FGFR))
Smad = TGFR
PIP3 = (~ PTEN) & (PI3K)
RAF = RAS
P21 = ((Smad) | (P53))
MEK = RAF
NFkappaB = AKT
AKT = PIP3
PTEN = P53
ERK = MEK
E2F = (~ RB)
cJUN = (ERK) | (JNK)
CyclinD = ((~ CDKNA) & (~ P21)) & (NFkappaB)
RB = (~ CyclinD)
BCLXL = (~ P53) & ((NFkappaB) | (AKT) | (JNK))
JNK = MEK
mTOR = (~ cJUN) & (AKT)
BAX = (~ BCLXL)
Beclin1 = (~ BCLXL) & (~ CASP)
MDM2 = (~ E2F) & ((AKT) | (P53))
P53 = (~ MDM2)
CyclinE = (~ P21) & (E2F)
CASP = (~ NFkappaB) & ((P53) | (Beclin1) | (BAX))
Autophagy = (~ mTOR) & ((NFkappaB) | (BCLXL))
Apoptosis = (CASP) | (TOXICITY)
Proliferation = (JNK) | (CyclinE) | (cJUN)
RAStemp = RAS & (~ KRASm)
secAG = 1
secVEGF = (NFkappaB)
secEGF = cJUN
secbFGF = ERK
secPDGFBB = Smad
secTGFbeta1 = Smad

Table S4. Boolean network of stellate cells

TNFR = TNF_external
IFNGR = IFGg_external
VEGFR = VEGF_external
EGFR = EGF_external
FGFR = bFGF_external
PDGFBBR = PDGFBB_external
TGFR = TGFb_external
PPARg = 0
RAS = ((VEGFR) | (EGFR) | (FGFR))
PI3K = ((PDGFBBR) | (RAS))
Smad = TGFR
STAT1 = IFNGR
RAF = RAS
NFkappaB = ((TNFR) | (AKT))
P38 = ((MEK) | (P53))
MEK = RAF
PIP3 = (~ PTEN) & (PI3K)
P53 = (~ MDM2) & (P38)
PTEN = P53
AKT = PIP3
ERK = (PDGFBBR) | (MEK)
AP1 = ERK
P21 = P53
MDM2 = ((AKT) | (P53))
Apoptosis = P53
Proliferation = (~ P21) & (AP1)
Migration = (AKT) | (AP1) | (Act)
Activation = ((~ STAT1) | (~ PPARg)) & (NFkappaB | Smad | AP1)
secVEGF = NFkappaB
secbFGF = ERK
secPDGFBB = Smad
secTGFbeta1 = Smad

Table S5. Boolean network of CD4 T cells

IL23R = IL23_external
IL2R = IL2_external
IL12R = IL12_external
TCR = AG_external
IL6R = IL6_external
IL10R = IL10_external
IL4R = IL4_external
IFNGR = IFNg_external
TGFbR = TGFb_external
IL18R = 0
IFNbR = 0
IL12R2 = (~ STAT6) & (IL12R)
IL18R2 = (~ STAT6) & (IL18R)
IL4R2 = (~ SOCS1) & (IL4R)
JAK3 = IL6R
STAT5 = IL2R
STAT6 = IL4R2
STAT3 = ((JAK3) | (IL23R) | (IL10R))
IRAK = IL18R2
NFAT = TCR
FOXP3 = ((~ STAT3) | (~RORYT) | (~GATA3) | (~TBET)) & ((FOXP3) | (TGFbR) | (STAT5))
TBET = ((~ FOXP3) | (~ RORYT) | (~ GATA3)) & (TBET | STAT1)
RORYT = ((~ FOXP3) | (~ TBET) | (~ GATA3)) & (RORYT | STAT3 | TGFbR)
SOCS1 = (TBET | STAT1)
GATA3 = ((~ RORYT) | (~ FOXP3) | (~ TBET)) & (GATA3 | STAT6)
JAK1 = (~ SOCS1) & (IFNGR)
STAT4 = (~ GATA3) & (IL12R2)
STAT1 = (JAK1 | IFNbR)
secIL17 = RORYT
secIL6 = RORYT
secIL4 = (~ (STAT1)) & (GATA3 | NFAT)
secIL10 = GATA3
secIFNg = (~ STAT3) & (TBET | STAT4 | IRAK | NFAT)

Table S6. Boolean network of macrophages

sigGMCSF = 0
sigIC = 0
sigIFNg = IFNg_external
sigIL1 = 0
sigIL4 = IL4_external
sigIL10 = IL10_external
sigLPS = AG_external
IFNGR = sigIFNg | IFNB
CSF2RA = sigGMCSF
IL1R = sigIL1 | IL1B
TLR4 = sigLPS | (~ FCGR)
FCGR = (sigIC & sigLPS) | (sigIC | sigIL1)
IL4RA = sigIL4
IL10R = sigIL10
STAT1 = IFNGR & (~ (SOCS1 | STAT3))
STAT5 = CSF2RA & (~ (STAT3 | IRF4))
NFKB = (IL1R | TLR4) & (~ (STAT3 | FCGR | PPARG | KLF4))
PPARG = IL4RA
STAT6 = IL4RA
JMJD3 = IL4RA
STAT3 = IL10R & (~ (FCGR | PPARG))
IRF3 = TLR4
ERK = FCGR
KLF4 = STAT6
SOCS1 = STAT6
IRF4 = JMJD3
IL1B = NFKB
IFNB = IRF3
secIL12 = STAT1 | STAT5 | NFKB
secIL10 = PPARG | STAT5 | JMJD3 | STAT3 | ERK
secTGFb = ERK | STAT3
secTNF = PPARG | STAT6 | JMJD3 | NFKB
secIL6 = PPARG | STAT6 | JMJD3 | NFKB

Table S7. Boolean network of CD8 T cell

sigTCR = AG_external
sigIL2 = IL2_external
sigIL12 = IL12_external
sigIFNg = IFNg_external
TCR = (~ PD1) & (TCR | sigTCR)
IL2 = TCR & (~ FOXO1) & sigIL2
IL21 = TCR
IL12 = TCR & sigIL12
TCRs1 = TCR
TCRs2 = TCRs1
STAT3s1 = STAT3
STAT3s2 = STAT3s1
FOXO1 = ((~ IL2) & (~ AKT)) & (TCRs2 | STAT3s2)
AKT = TCR & (~ PD1)
NR4A1 = NFATC1 | PD1
AP1s1 = AP1
AP1s2 = AP1s1
PD1 = (~ TBET) & (((AP1s2 & AP1s1 & AP1) | FOXO1) | NFATC1)
AP1DNA = (AP1s2 | AP1s1 | AP1) & (~ BCL6)
BLIMP1 = (BATFIRF4 | IL2 | AP1DNA)
IRF4 = NFKB & (~ NR4A1)
BCL6 = (FOXO1 | BATF) & (~ IRF4)
TCF1 = BCL6
BATFIRF4 = BATF & TCR
BATF = (IL12) | (IL21)
NFATC1s1 = NFATC1
NFATC1s2 = NFATC1s1
NFATC1meds1 = NFATC1med
NFATC1meds2 = NFATC1meds1
NFATC1lows1 = NFATC1low
NFATC1lows2 = NFATC1lows1
NFATC1 = NFATC1meds2 | NFATC1s2
NFATC1med = NFATC1lows2
NFATC1low = NFATC2
IFNg = (NFATC2) & (AP1) & (~ TCF1) & (sigIFNg)
NFKB = TCR
NFATC2 = TCR
AP1 = TCR | AP1
IL21s1 = IL21
STAT3 = IL21s1
TBET = IL12
secIL10 = STAT3
secIFNg = TBET
secGZMA = TBET
