# Supplementary material for: A generalizable data-driven multicellular model of pancreatic ductal adenocarcinoma
Source: Gigascience. 2020 Jul 22;9(7):giaa075. doi: 10.1093/gigascience/giaa075 (PMC7374045; doi:10.1093/gigascience/giaa075)
Supplement: giaa075_Response_to_Reviewer_Comments_Original_Submission [file giaa075_response_to_reviewer_comments_original_submission.pdf]

Author's Response To Reviewer Comments

Close

Dear Editor,

Thank you for giving us the opportunity to submit a revised draft of the manuscript with titled “A generalizable data-driven multicellular model of pancreatic ductal adenocarcinoma” to the “Data-Driven Multicellular Systems Biology” thematic series of GigaScience.

We appreciate the time and effort that you and the reviewers have dedicated to providing your valuable feedback on my manuscript. We are grateful to the reviewers for their constructive and insightful comments on our manuscript. We incorporated most of the changes suggested by the reviewers. For clarity, we used red font color for the changes in the manuscript.

A point-by-point response to the reviewers' comments and concerns is attached to this letter.

We look forward to hearing from you in due time regarding our submission and to respond to any further questions and comments you may have.

Sincerely,
Ilya Shmulevich
Alexander Ratushny

Reviewer 1

We thank the reviewer for the thorough examination of the manuscript. We have incorporated all the reviewer's suggestions involving minor edits. The changes are in red font.

6. Line 158: It seems that you assume diffusion is unaffected by cells in the environment (e.g. sequeses at receptering of molecultors, or differing diffusion rates due to non-homogeneous densities). This should be mentioned as it is a simplification of the real biological system.

We thank the reviewer for highlighting this issue. Indeed our model assumes that the concentration of cytokines is not affected by cellular uptake. This is now mentioned in the “Cell-cell communication via diffusion of cytokines” subsection, line 178. Moreover, a list of the main model assumptions are stated explicitly in the first paragraph of the “Modeling framework” section.

11. Line 200: You state the "last K steps", but nowhere could I find out how long your simulations were run for or whether you checked that they had reached steady state. Including specific details about the length and conditions for ending each is needed, either here or in the Methods

We have added a new Figure (Figure 2 in the revised manuscript) that shows that a simulation reaches steady state in approximately 25 time steps. In all our results, we have used K=200 from simulations of 400 time steps. These details are now included in the manuscript starting approximately in line 266.

Line 248: "some of these parameters, such as cellular fractions, are estimated from data available in TCGA" - how precisely was this done. Details need to be added to Methods and referenced here

We have added (in line 311) a brief description and a reference to the method section in which cellular fractions are computed. Moreover, we have added additional details to the subsection “Estimation of cell fractions” in which we describe the methods used to estimate cell fractions.

22. Line 259: Is it true that cancer survival *requires* an asymmetric cytokine mediated communication? This is a strong statement and to me it is merely a possible explanation, not necessarily

the only one.

We agree that asymmetric cytokine interaction is just a possible explanation suggested by our model. Accordingly, we rewrote the statement: "In summary, these results show that an asymmetric cytokine mediated communication between stellate and cancer cells plays a role in the observed positive effect on cancer survival exerted by stellate cells", line 444 in the "The role of paracrine and autocrine loops" subsection.

23. Line 389: It is strange to reference Figure 1 so far through the paper. It may be worth considering restructuring the figures a little or updating the later ones to include the point you are trying to show. This would help with reading.

We thank the reviewer for this suggestion, we have restructured the figures and added more figures for clarity. We think these improve the manuscript substantially. Figure 1 is now referenced in the "ModelingFramework" section.

26. Line 417: I agree that refining the models is potentially a valuable direction, but what about the issues it brings in regard to increasing numbers of parameters and the risks of overfitting due to these? It would be worth discussing also the role that the model has and where the trade off in complexity needs to be made.
33. Line 518: This is a reiteration of the question above about over fitting due to more complex models having so many parameters. It would be good to mention this potential issue here.

Although additional data does not necessarily imply a substantial increase in model complexity (number of parameters); we agree that a discussion on how more complex and refined models can generate problems such as overfitting, especially when no additional data is integrated. Accordingly we extended the "Discussion" section to discuss these problems. Specifically, we added an additional paragraph (second paragraph of the "Discussion" section) that discusses the complexity of the proposed model; and in line 651 (paragraph 9 of "Discussion") we added the potential problems of adding more refined models.

27. Line 457: While I agree that there appears to be a correlation between perturbations of bFGF and cancer cell apoptosis, the graphs do not show the variability across the population/simulations. Why are these not included on the plots? If this is high, then the 0.1 increase observed may be negligible. Some further description on this topic is needed.

We have extended the perturbation analysis and updated the figures including the reviewer's suggestions. Specifically, we added the variability (standard deviations from multiple simulations as error bars) into the plots that show how cancer cell apoptosis change as a function of perturbation fractions, see Figures below and Figure 8 C and D.
Moreover, we extended the section "Exploration of therapeutic interventions" adding more analysis and a new figure (Figure 8). Specifically, we computed "p-values of the null hypothesis that the slope between perturbation fractions and cancer apoptosis is zero, Figure 8B" we found several samples with significantly small p-values, suggesting that a bGFG perturbation may induce apoptosis for these samples.

30. Line 503: What about dynamic expression profiles? Is an assumption that the internal regulatory networks remain in a single steady state realistic?

Yes we assumed the average (bulk) gene expression is on steady state. This limitation is mainly due to the nature of the data used for model calibration/validation, which represent only a single time point of cancer progression; we added a discussion of this assumption in line 589, fourth paragraph of the "Discussion" section.

37. Further details regarding all of the supporting software used (especially for parameter fitting) should be provided (e.g. version number and citation where relevant).

We have made the code and documentation of the modeling work publicly available in github. Moreover, we included a simple two-cell model example with a detailed documentation that describes how the Boolean networks and cytokine networks are specified, so users can create their own multicellular models.
Information about the code can be found in the new section "Availability of source code and

requirements” of the main document.

Reviewer 2

“The scope of the paper is not clearly defined. If this is a paper about a general modelling framework, as stated in some parts, the framework should be presented in its generality. This is not done. If this is a validation of a PDAC model, a more rigorous analysis with robust indicators of performance should be provided than those presented.”

Our scope includes both, to study the immune-cancer interactions in Pancreatic cancer and to develop a multicellular model that is generalizable to study other cancer types. We have extended our results section to include more details of our analysis on Pancreatic Cancer, see new Figure 8 and Figure 2. Moreover, we made the code available on Github (https://github.com/boaguilar/multicell_boolean_networks); the code includes an abstract example of a system with two cell types with documentation so users can simulate other systems, see the new section “Availability of source code and requirements” for more information about the code.
We have also substantially revised our methodology, incorporating additional information of the model and suggestions from the reviewers.

“The novelty of the study is not clear. Whilst the application to PDAC TME has some novelty, frameworks merging Agent Based modelling and Boolean Networks have already been presented before, and an open access, open source implementation of this framework has been published by Gigascience earlier this year (Voukantsis et al, GigaScience, Volume 8, Issue 3, March 2019, Gigascience Multicellular Systems Biology Series issue 1). The reference to this and other publications on this methodology are missing. They should be added, and explanation of the difference in the methodology between this study and the previous implementations should be provided.”

We thank the reviewer for letting us know about recent works that are similar to the present modeling approach. We added a new paragraph in the introduction (line 97, fifth paragraph of Introduction) referencing a couple of papers that combine ABMs and BNs. Moreover, we highlighted the main difference between the modeling work presented in this work and the two previously modeling works (Voukantsis et al, GigaScience, Volume 8, Issue 3, March 2019 and Letort et al. Bioinformatics, Volume 35, Issue 7, 01 April 2019, Pages 1188–1196). In summary the main difference is that the present model focuses exclusively on modeling cellular states of cancer and how these are affected by communication with other cells of the tumor microenvironment, whereas the previous models aim at modeling tumor growth.

“The authors statement "we developed a modeling framework designed to" is not matched by the publication of the actual framework for others to reuse and benefit from. No mention to open implementation is contained in the manuscript, nor sufficient details are provided to reproduce the implementation.”

We agree that the availability of the code will improve reproducibility. Accordingly, the code to perform simulations, documentation, and license are now publicly available in Github. Details of the code are now available in the new section “Availability of source code and requirements”. Moreover, we revised the section “Modeling framework” and “Methods” (changes in the main manuscript are in red) incorporating reviewers comments and adding more details of the modeling approach.

“The authors answered yes to the question on data and code availability, but no code nor access link to the model (e.g. github repository) were provided, so there is no evidence to support the open data and open code statement.”

The code to perform simulations, documentation, and license are now publicly available in Github (https://github.com/boaguilar/multicell_boolean_networks) .

“The generality of the framework is stated but not demonstrated as only a specific model of PDAC is presented and no general open access environment is provided for other to test, modify and apply to other scenarios.”

We have given the mathematical statements that underlie the framework, and consider them, after a

substantial revision, to be quite general. The code that is now publicly available includes a basic two cell model example with documentation about model specification, so that users can use and change the model at their convenience.

“The description of the Boolean network (BN) implementation lacks important details. For example, it is unclear whether the stochastic nature of the Boolean networks relies only on the rate of mutations introduced, and whether the same types of mutations are introduced at the same time. More technically, does the \gamma vector change in each cell?”

The gamma vector is the same for all the cells of the same type. Moreover, the perturbation probability (q) does not change during simulations and is the same for all genes. This is now stated in the model description, line 169.
Moreover, a list of the main model assumptions are stated explicitly in the first paragraph of the “Modeling framework” section (line 124). One of the highlighted assumptions is that the parameters that characterize cell behaviour are the same for all the cells of the same type. Thus, all cells of the same type are governed by the same BN and share the same parameters of cell communications.

“It is not clear how the state of each regulatory gene in the BN is defined as a function. The authors statement "determined by the state of its Boolean nodes" (page 6, line 137) is not followed by a detailed description.”

The state of a cell is defined mathematically as a binary array (X_i in the main text), which is determined by the Binary values of the genes associated to a cell type. We agree that the particular statement regarding States may be confusing in that part of the manuscript, so deleted for better clarity. The cellular state definition is now in line 145 (“Cells as Boolean networks subsection”), defined as an array (X_i) of 0s and 1s.

“F_j (page 6, line 125) is not well defined. Is it a function (boolean expression) that varies across regulatory nodes and was obtained by optimising the BN to existing data? Is it a PNN ready graph with the boolean functions already calculated? Has there been any fitness of the GRN with PDAC?”

Fj^t is a Boolean function that determines the value of gene j cell i at the next time step t+1. Every gene (j) has its own updating function. However we assume all the cells of the same type have the same Boolean Network, i.e, the same set of function { F_j }. Moreover, these Functions are not optimized in this work, which is an interesting possibility; instead, they are obtained from literature and represent existing knowledge of intracellular regulation. This description is now added in the “Cell as Boolean Network”, line 157 approximately.
Moreover, the possibility of including BNs in the optimization pipeline is included in the “Discussion ” section (line 581).

“It is unclear whether the update of the networks is synchronous or not.”

The Boolean networks are updated synchronously. We made this explicit in the subsection “Cells as Boolean networks”, line 141 and 142.

“Only some aspects of the TME have been accounted for. For example, hypoxia is known to be an important feature of the PDAC microenvironment and is not present in the model, or if it is present, there is not description on this. Furthermore, other details are missing for TME features, for example, no boundary conditions for the diffusion are provided.”

Since the main objective of this work is to develop a model that focuses exclusively on the interplay between cell-cell communication and gene regulation, we included only the interactions and processes that are related to our objective. Many processes and interactions such as oxygen uptake, mechanical aspects of TME, cell motility are not included. This is mentioned in a new paragraph in the Introduction, line 102; and also discussed in the second paragraph of the Discussion section.

“There is no justification on the attempt to fit the expression of the genes to optimize the model parameters. It is well known that expression doesn't necessarily implies changes in the gene network, so meticulous examination is needed to conclude to a specific mechanism as a causal effect.”

Our rationale was to use gene expression data to estimate parameters of cell-cell communication and mutation fractions only, the parameters are listed in Table S2 of the Supporting material. We used the

gene expression as a proxy for the readouts in the model, if other types of data are available, we could use those as well.
Moreover, we assume that the gene regulatory networks used of all cell types are static, so we did not optimize the regulatory network. A possibility of optimizing in the actual Gene regulatory networks was added to the Discussion (line 581).

“It is not clear how the model would transfer to other gene networks or cancers as there is no discussion on functionality that would enable this.”

We have made the code and documentation of the modeling work publicly available in github (https://github.com/boaguilar/multicell_boolean_networks). Moreover, we included a simple two-cell model example with a detailed documentation with a description of how the Boolean networks and cytokine networks are specified, so users can create their own multicellular models.
We have also revised the methodology and included additional details for a clearer description of the model.

“How the networks plug in with the Agent Based Modelling (ABM) is unclear. A description on this is not provided, so the validity of this aspect cannot be evaluated. If this has been done similarly to previous implementations this should be stated, and an explanation should be provided of how this implementation differs with respect to previous ones. Otherwise, if this is done in a novel way, the methodology for this crucial aspect should be provided.”

Our approach is similar to other approaches that combine multicellular agent based models with Boolean networks in the sense that 1) extracellular variables are used to update the state of Receptor nodes, and 2) the output nodes of the Boolean networks can affect the extracellular variables. Typically the extracellular variables include signalling molecules which are modeled by the Diffusion equation. Our implementation is similar to the one developed by Olimpo et al. (Reference 42 of the revised main text) in which the diffusion of molecules are modulated by the possibility of two secretion rates, low or high, corresponding to the Boolean states of some nodes of the Boolean networks. Moreover, the spatial concentration of signaling molecules can affect the state of the cellular Boolean networks, the receptor of cells are activated if the local concentration of cytokines are greater than a user defined threshold.
We have reorganized and changed the ‘Modeling framework’ section to clarify the coupling. Specifically, we added a new subsection “Integration of gene regulation and cell-cell communication” (line 199).

“The Biocellion software is used as implementation environment. This is not an open source code so this needs to be clarified and addressed as at the moment the work presented in this paper could not be reproduced by the community. Furthermore, the details on how the implementation was carried out within Biocellion are missing, so even users of Biocellion would not be able to implement this simulation and reproduce the results.”

We thank the reviewer for pointing out this issue. It is true that Biocellion is not open source. However, the work presented in this paper can be reproduced. The code of the model that runs in Biocellion is open and now freely available in github. Moreover we provide examples of how to set up a model and run simulations of the model in Biocellion. This is possible because the Biocellion binary is available for academic use, and the code of the model is separated from the Biocellion code.

“The last paragraph on therapeutic intervention contains very limited results, and no validation, but presents strong conclusions. This should be extended to include validation or omitted.”

Our intention with the therapeutic intervention analysis was to show the potential of the modeling approach of this work. Accordingly, we have revised the conclusions derived from this analysis.
Moreover, we have extended the analysis of therapeutic interventions, see Figure 8 of the revised manuscript. Basically we computed p-values of the positive correlation of apoptosis degree and fraction of cells affected by intervention.
Although a rigorous validation is missing in this section, we consider that this section shows how this type of modeling approach can generate hypotheses that can be further explored.

“The conclusions of the study seem overoptimistic given the overall lack of correlation between the simulation and the PDAC data. A more critical discussion should be presented reflecting this.”

We have revised the “Discussion” section substantially noticing that the correlation between simulations and PDAC data can be improved using additional data and more refined models.

Reviewer 3

1) The literature review is generally comprehensive and inclusive. The authors should be aware of other efforts to integrate larger-scale molecular-scale networks in to agent-based modeling frameworks, including Letort et al's recent work to integrate Boolean signaling networks into agent-based models, such as PhysiBoSS (DOI: 10.1093/bioinformatics/bty766), which combines the MaBoSS open source package for Boolean networks with PhysiCell. Voukantsis et al. wrote a nice article in GS about integrating large gene networks into an agent-based model (DOI: 10.1093/gigascience/giz010).

We thank the reviewer for pointing us to similar modeling efforts. We included them in our literature review and highlighted the main differences between our modeling approach and those modeling efforts, please see the new paragraph (line 97, red font) in the "Introduction" section.

2) It may be worth mentioning briefly to the readership why Boolean networks are considered instead of ODE networks. (Presumably for efficiency and to reduce the number of parameters.)
Include a section on that.

Indeed, we used BNs because efficiency and to minimize the number of parameters of the multicellular model. This is now noted in the Introduction section, line 92 in red.

3) The authors should more explicitly state (between lines 105 and 111, probably) whether this is a lattice-based model or off-lattice. Moreover, the method section does not state the movement rules for cells. Are they motile? What are the rules for placement of daughter cells after division? What about cell adhesion and repulsion mechanics? If the cells are static in this model, that's fine, but it needs to be stated clearly.

The cells are static in the sense that the initial positions of cells remain constant during simulations. This and other important assumptions of the model are now listed in the initial paragraph of the section "Modeling framework" (red font).
Moreover, our model is lattice free as the positions of cells are obtained from random point processes. This is now explicitly stated in the section "Tissue Architecture", line 221 of the revised manuscript.

4) Around line 127, given that signaling factors are diffusion, non-Boolean fields, how do you decide presence / absence of a cytokine for receptors? Are there thresholds?

Yes, the receptor nodes are activated if the concentration of the corresponding cytokine is greater than a threshold. This is noted explicitly in the new subsection "Integration of gene regulation and cell-cell communication" (starting at line 212).

5) Around line 163, I don't see any cellular uptake of cytokines, even when binding to receptors. Please comment on their model assumptions and possible impact (e.g., on spatiotemporal behavior of cytokine gradients and distributions).
.
For simplicity, we did not include cell uptake in the model. This assumption is now stated in line 178. It is also discussed in the "Discussion" section line 606.

6) The authors state on line 169 that contact-based chemical interaction can be modeled with short diffusion distances lambda = sqrt(D / gamma). This is the diffusion length scale, but numerically, this spatial scale is not going to be resolved at dx ~ lenght scale (instead of dx ~ 0.1 * length scale). Can the authors discuss why this is fine? (I think it is, since this is just approximating D = 0 and setting an cytokine indicator > 0 nearby for simple contact interactions.)

We thank the reviewer for pointing this issue. Reducing the Diffusion coefficient increases the spatial decay of signal concentrations. We did not find any numerical problem in the simulations for lambda values smaller than the spatial resolution. However, the grid spacing length used for solving the PDE is the minimum possible effective pairwise interaction between cells.
We used a grid spacing length comparable to the cell sizes so the minimum effective interaction distance is enough to approximate interaction between nearest neighboring cells.
This is summarized in the subsection 'Integration of gene regulation and cell-cell communication', line

217.

7) It would be good to include a sample image of an initial spatial distribution. I think it ought to be in the main manuscript, rather that SI. Likewise, I think it would be illustrative to readers to show the time series of a single simulation (and to provide a video as supplementary material) to better illustrate the model. (And then the authors will run it in high throughput to generate scalar metrics.)

We thank the reviewer for the suggestion. We added a new figure (Figure 2) in the main manuscript in which we included the spatial configuration of the two cell model (cancer and stellate cell model) and the trajectory of one simulation showing how the fraction of cancer cells in proliferation changes during the simulations.

8) I really like the describe initial state generation. It would be nice of the authors to provide source code for that routine.

The code is now available to the public Github repository (https://github.com/boaguilar/multicell_boolean_networks). The code includes examples of how to setup the system (including the initialization of the spatial distribution of cells) and how to perform simulations.

9) The model was implemented in Biocellion. Will the source code be shared?

Unfortunately, Biocellion is not open source. However, the code of the model that runs in Biocellion is open and now freely available in github, see the new section “Availability of source code and requirements” for details of the code. In addition to the code we also provide examples of how to set up a model and run simulations. This is possible because the Biocellion binary is available for academic use, and the code of the model is separated from the Biocellion code.

10) It wasn't completely clear to me if cells are proliferating, apoptosing, moving, etc. (Missing details in method). If they are static (e.g., flagged as cycling or apoptotic, but not simulated to the point of dividing or being removed from simulation), this needs to be more clearly stated.

We thank the reviewer for highlighting this issue. Since our main goal is to study the interplay between cell-cell communication and gene regulation, other interactions and processes, such as cell motility and mechanical interactions, are not included in the model.

For clarity, we have included all the main assumptions of our model, first paragraph of the “Modeling framework” section. The implications of the assumptions are discussed in line 584 (Discussion section).

11) It's still not fully clear how you map from genotype to phenotype. Can you please clarify this and perhaps add some brief text? Figure 2 is very hard to follow (the lines cross over lines). Perhaps break out "subfigures" that show which graph nodes map to apoptosis, proliferation, and migration, and how the states of those nodes turn them on? (Linear sum + activation function?)

We updated Figure 2, which now is Figure 3 in the manuscript.

We required that the Boolean networks possess phenotypic nodes that characterize the phenotypic state of a cell. Figure 2 shows an example of the two cellular system in which the Boolean network of a Stellate cells have three phenotypic nodes (Apoptosis, Proliferation, and Migration) and the cancer cells have three phenotypic nodes (Apoptosis, Autophagy, and Proliferation).

The phenotype of the tissue segment is characterized by the fraction of cells with the corresponding phenotypic node in ON. We added a text with this description in line 268.

12) The actual time scale of the simulations was not made clear. How many time steps? How much physical time? DId you simulate a very short time, and hence neglect proliferation, apoptosis, cell growth, cell movement, etc? Please make the time scale

In this work we are considering physical time scales of hours, determined for gene regulation. We added a list of modeling assumptions in the first paragraph of the “Modeling framework” section, in which we stated the time scale.

13) The calibration protocol is nicely done. This represents some good work to connect TCGA with behavioral parameters in the ABM.
14) Likewise, I liked your data analysis of the agent models. Many modelers are struggling for the right metrics to analyze their spatial models, and fall back to destroying most of the information to just plot growth curves, etc.
17) Looking for correlations between calibrated model parameters and PDAC subtypes is novel. It allows us to integrate multiple forms of knowledge (reflected in the model structure) with the data.

We are very thankful for the reviewer comments and encouragement.

15) I think the authors could improve their impact by (a) providing a nice, clean diagram describing the analysis workflow [showing (i) how one analysis module flows into the next, and (ii) a table of inputs, technique name, outputs, and what's learned for each block in your flow], (b) potentially open sourcing the analysis workflow. It would be very beneficial to the field to see the analysis of multicellular models become more standard, much like has happened for molecular models.

A diagram of the analysis workflow is included in Figure 1B of the original manuscript. For clarity, we decided to cut Figure 1B and place it in the "Patient-specific models for TCGA samples", Figure 5 of the revised manuscript. Moreover, we added a new table (Table 3 which is located approximately in line 490) which includes inputs, software name, outputs, usage description of the analysis workflow, as suggested by the reviewer.

The workflow consists of different methods and software. It is challenging to integrate all the methods into a single open source. However, we are providing the model source code and all the processed files that are directly used by the model (cell proportions, the mutational states, the gene expression of cancer cells, etc.) to perform simulations of TCGA samples, see the new section "Availability of source code and requirements" section and also Additional Files 3-5.

16) Similarly, the authors could have some nice impact in visualizing the calibration protocol, perhaps similarly to (15).

We have included a diagram of the calibration protocol as a new Figure (Figure S2).

18) This paper was a great case for high-throughput computing with agent models. May want to look at literature of Gary An who has written quite a lot on this topic.
Moreover, the authors might find our most recent work with Argonne National Lab useful for comparison and future use: we built a decision tree binary classifier to divide parameter space into "meets a design objective" (e.g., cancer cell population controlled) vs. "does not meet objective", and adaptively choose parameter sets to refine the decision boundary. This reduced the needed simulations (for our study) from 10^7 to 10^4 simulations per design goal. Due to the increased cost, we were able to next the design goals to assess the topology of the design parameter space. (DOI: 10.1039/c9me00036d; building on earlier HPC investigations in DOI: 10.1186/s12859-018-2510-x) I think the methods in your work and ours could be combined for more adaptive simulation runs and improved data analysis.

We strongly agree with the reviewer; the parameter exploration of the model presented in this work can be improved substantially by using the Active Learning method in the reviewer's suggested method; thus it is possible to obtain new and more robust conclusion regarding the influence of cell-cell communication on cancer behaviour. Since the exploration method is based on HPC platform, we think it can be combined with our model which is implemented in Biocellion which is also an HPC platform.
We think it can be used for determining the parametric regions in which the interaction between Stellate cells and Cancer cells increases cancer proliferation. This will be very useful in future applications of the model. We have mentioned this and other potential improvements of model exploration in the Discussion section, line 636.

19) This is provocative: "For instance, there is a debate concerning whether stroma-cancer interactions are associated with progression of pancreatic cancer or, rather, provide protective measures." To the extent that you can, it would be good to show (and clearly state) that your model identifies ways the same cell types can be both helpful and harmful to the tumor, depending upon context. It's an important point that your model can make.

20) Optionally, you might want to show that the same model, without spatial aspects (well-mixed) gives different results.

Indeed the stroma-cancer interplay was one of the motivations to include the two cell model (cancer and stellate cells) in the analysis. Figure 4 of the revised manuscript shows that the intercellular interaction between cancer and stellate cells can be harmful for cancer cells increasing apoptosis and helpful for cancer cells increasing proliferation. This is evident by the significant positive (red) and negative(green) correlations of the model parameters with the apoptosis and proliferation (rows in the heatmap). We have revised the section “Analysis of the interplay between cancer and stellate cells” and included the suggested points; new and modified text are highlighted in red.

Close
