# Supplementary material for: A generalizable data-driven multicellular model of pancreatic ductal adenocarcinoma
Source: Gigascience. 2020 Jul 22;9(7):giaa075. doi: 10.1093/gigascience/giaa075 (PMC7374045; doi:10.1093/gigascience/giaa075)
Supplement: giaa075_Response_to_Reviewer_Comments_Revision_1 [file giaa075_response_to_reviewer_comments_revision_1.pdf]

Author's Response To Reviewer Comments

Close

Dear Dr. Nogoy,

Thank you for giving us the opportunity to submit a revised draft of the manuscript entitled "A generalizable data-driven multicellular model of pancreatic ductal adenocarcinoma" to the "Data-Driven Multicellular Systems Biology" thematic series of GigaScience.

We appreciate the time and effort that you and the reviewers have dedicated to provide us with valuable feedback on our manuscript.

Regarding your suggestion concerning Biocellion, we added a statement (see Availability of source code and requirements) describing that the code is self-contained, that all the dependencies are in the repository, including Biocellion1.2 which is free for academic use, making it unnecessary for users to download or install Biocellion; and that the code is ready to be compiled and executed.

Furthermore, we ported the code to CodeOcean and included a reproducibility statement in the paper (https://doi.org/10.24433/CO.2337238.v1). The capsule includes a toy example of the model as well as the scripts to run the two models described in the paper.

We are also attaching with this letter a response to reviewer #2. We look forward to hearing from you regarding our submission and to respond to any further questions and comments you may have.

Sincerely,

Ilya Shmulevich
Alexander Ratushny

Reviewer #2: I'm generally happy with the revision and the deposition of the source code, however it is still not clear if this can be run completely independently of the Biocellion environment. I could not find a clear statement on this. This is important and pointed out by more reviewers. If this is fully clarified, and any dependency has been eliminated so that the code can be run irrespective of Biocellion, I'm happy that this complies with the premises of the journal. Alternatively access to Biocellion source code should be provided for the relevant parts.

The code that accompanies the paper is open, however it needs Biocellion to run and generate the simulations; this is similar to a code in Matlab which needs the MATLAB framework to run. except that the Biocellion framework is free for academic use.
To make the code self-contained and easier for users to run simulations, the biocellion framework and some dependencies are included in the github repository. This way users don't need to install Biocellion.
Moreover, for reproducibility and to make it easier for users to try the code we created a capsule in OceanCode which includes all the executable code (https://doi.org/10.24433/CO.2337238.v1), and dependencies. Code Ocean makes sure that code is reproducible.
We added a statement about dependencies and the link of the Code Ocean capsule in the "Availability of source code and requirements" section.

Close
