# Supplementary material for: A generalizable data-driven multicellular model of pancreatic ductal adenocarcinoma
Source: Gigascience. 2020 Jul 22;9(7):giaa075. doi: 10.1093/gigascience/giaa075 (PMC7374045; doi:10.1093/gigascience/giaa075)
Supplement: giaa075_Reviewer_1_Report_Original_Submission — Thomas Gorochowski -- 8/30/2019 Reviewed [file giaa075_reviewer_1_report_original_submission.pdf]

Reviewer Report

Title: A generalizable data-driven multicellular model of pancreatic ductal adenocarcinoma

Version: Original Submission **Date:** 8/30/2019

Reviewer name: Thomas Gorochowski

Reviewer Comments to Author:

In this work the authors present a new multi-scale model of cancerous tissue that combines a Boolean network representation of cellular regulatory networks for multiple cell types, within a spatial agent-based framework to capture the diffusion of signaling molecules between cells. This model is then used in a number of interesting ways. Of particular note, some interesting parameterization techniques and data sets are used to allow for better representation of non-homogenous tissues across patients, and possible gene perturbations analyzed using this diverse set of models. This allowed a number of existing hypothesizes to be tested and new interventions assessed across models capturing known heterogeneity across a population.

While I am not well placed to comment on the significance of the findings in regard to cancer biology, I do believe that the computational approaches employed are novel and will be of broad interest to multi-scale modelers across many other domains of biology. The statistical methods employed seem reasonable and would have sufficient power to support the findings presented. Overall, I found the paper exceptionally clear and well-written and commend the authors on the effort they have put in to producing an accessible piece of interesting research. I have only a number of minor comments and corrections that I would like the authors to address and ask that availability of their models is directly stated in the main text:

1. Line 100: "ensemble modelling approach" - It may be worth expanding a little on what this is for less computationally focused readers.
2. Line 121: "at t + 1 (next time step)" would read better "at time step t + 1 (i.e. the next time step)".
3. Line 125, 156, 163, 194, 202, 205, 254, 260: Equations have a strange "…" after them. This should be removed and the equation number righthand justified.
4. Line 136: It would be helpful to add a citation that explains why the system can be described by an ergodic Markov chain with unique steady-state distribution.
5. Line 151: It may be clearer to use the term "regular 3D lattice", although I leave this up to the author.
6. Line 158: It seems that you assume diffusion is unaffected by cells in the environment (e.g. sequestering of molecules at receptors, or differing diffusion rates due to non-homogeneous densities). This should be mentioned as it is a simplification of the real biological system.
7. Line 171: "We have constructed" would read better "We constructed".
8. Line 183: "we have used" would read better "we used".
9. Line 185: "We have set up" would read better "We set".
10. Line 199: "astronomical" would read better "large".
11. Line 200: You state the "last K steps", but nowhere could I find out how long your simulations were run for or whether you checked that they had reached steady state. Including specific details about the

length and conditions for ending each is needed, either here or in the Methods.
12. Lines 214, 229, 237, 367, 369, 370, 382, 455, 591: TGF seems to have a strange square character which I suspect was generated during conversion. Please make sure this is corrected throughout. I've included the locations above that I found but recommend checking carefully throughout after the conversion process.
13. Line 248: "some of these parameters, such as cellular fractions, are estimated from data available in TCGA" - how precisely was this done. Details need to be added to Methods and referenced here.
14. Line 258: The new paragraph formatting needs updating (indenting and no new line).
15. Line 261: "We have used" would read better "We used".
16. Line 263: "Initialize the theta_i" would read better "Initialize theta_i". Also, there should be a space between "theta_i" and "randomly".
17. Line 264: "Temperature" should be "temperature".
18. Line 268: "Change the temperature" would read better "Update the temperature".
19. Line 290: Be specific about where in the Supplementary Material the matrix can be found (precise file).
20. Line 296: "(Figure S3 in Supporting Material)" could be changed to "(Supporting Material, Figure S3)". This stylistic change could be made throughout but is up to the authors. It would be good to be consistent with how the Supplementary Material is referenced though.
21. Line 310: Please provide the expression values as supplementary data. It is essential that the results can be reproduced if necessary.
22. Line 259: Is it true that cancer survival *requires* an asymmetric cytokine mediated communication? This is a strong statement and to me it is merely a possible explanation, not necessarily the only one.
23. Line 389: It is strange to reference Figure 1 so far through the paper. It may be worth considering restructuring the figures a little or updating the later ones to include the point you are trying to show. This would help with reading.
24. Line 403: New paragraph not indented.
25. Line 408: "described in Methods" would read better "described in the Methods".
26. Line 417: I agree that refining the models is potentially a valuable direction, but what about the issues it brings in regard to increasing numbers of parameters and the risks of overfitting due to these? It would be worth discussing also the role that the model has and where the trade off in complexity needs to be made.
27. Line 457: While I agree that there appears to be a correlation between perturbations of bFGF and cancer cell apoptosis, the graphs do not show the variability across the population/simulations. Why are these not included on the plots? If this is high, then the 0.1 increase observed may be negligible. Some further description on this topic is needed.
28. Line 478: "a key player" should read "a potential player".
29. Line 479: "autocrine is loop is" would read better "autocrine loop is".
30. Line 503: What about dynamic expression profiles? Is an assumption that the internal regulatory networks remain in a single steady state realistic?
31. Line 507: I'd recommend including a citation to a good optimization review.
32. Line 516: "much better compared to random" would read better "much better than random".
33. Line 518: This is a reiteration of the question above about over fitting due to more complex models

having so many parameters. It would be good to mention this potential issue here.
34. Line 564: "Cartoon representation" would read better "Schematic".
35. Figure 5 & 6: Please include the number of samples and simulations used in all cases.
36. Figure 5: The text is not very readable. I'd recommend increasing the font size in all figures, so it is a minimum of 8 pt at printed size.
37. Further details regarding all of the supporting software used (especially for parameter fitting) should be provided (e.g. version number and citation where relevant).

Methods

Are the methods appropriate to the aims of the study, are they well described, and are necessary controls included? Choose an item.

Conclusions

Are the conclusions adequately supported by the data shown? Choose an item.

Reporting Standards

Does the manuscript adhere to the journal's guidelines on minimum standards of reporting? Choose an item.

Choose an item.

Statistics

Are you able to assess all statistics in the manuscript, including the appropriateness of statistical tests used? Choose an item.

Quality of Written English

Please indicate the quality of language in the manuscript: Choose an item.

Declaration of Competing Interests

Please complete a declaration of competing interests, considering the following questions:

- Have you in the past five years received reimbursements, fees, funding, or salary from an organisation that may in any way gain or lose financially from the publication of this manuscript, either now or in the future?
- Do you hold any stocks or shares in an organisation that may in any way gain or lose financially from the publication of this manuscript, either now or in the future?
- Do you hold or are you currently applying for any patents relating to the content of the manuscript?

- Have you received reimbursements, fees, funding, or salary from an organization that holds or has applied for patents relating to the content of the manuscript?
- Do you have any other financial competing interests?
- Do you have any non-financial competing interests in relation to this paper?

If you can answer no to all of the above, write 'I declare that I have no competing interests' below. If your reply is yes to any, please give details below.

I declare that I have no competing interests

I agree to the open peer review policy of the journal. I understand that my name will be included on my report to the authors and, if the manuscript is accepted for publication, my named report including any attachments I upload will be posted on the website along with the authors' responses. I agree for my report to be made available under an Open Access Creative Commons CC-BY license (http://creativecommons.org/licenses/by/4.0/). I understand that any comments which I do not wish to be included in my named report can be included as confidential comments to the editors, which will not be published.

Choose an item.

To further support our reviewers, we have joined with Publons, where you can gain additional credit to further highlight your hard work (see: https://publons.com/journal/530/gigascience). On publication of this paper, your review will be automatically added to Publons, you can then choose whether or not to claim your Publons credit. I understand this statement.

Yes Choose an item.
