# Supplementary material for: A generalizable data-driven multicellular model of pancreatic ductal adenocarcinoma
Source: Gigascience. 2020 Jul 22;9(7):giaa075. doi: 10.1093/gigascience/giaa075 (PMC7374045; doi:10.1093/gigascience/giaa075)
Supplement: giaa075_Reviewer_2_Report_Original_Submission — Francesca Buffa -- 10/15/2019 Reviewed [file giaa075_reviewer_2_report_original_submission.pdf]

Reviewer Report

Title: A generalizable data-driven multicellular model of pancreatic ductal adenocarcinoma

Version: Original Submission **Date:** 10/15/2019

Reviewer name: Francesca Buffa

Reviewer Comments to Author:

General comments

This paper presents a modelling study in PDAC based on a 3D model of PDAC growth. The novel aspects of the study are the application of this methodology to PDAC and the comparison of the results with the observations from cancer cohorts. The results show some agreement which is encouraging as the methodology used is only recently been proposed and has not been validated. Weak aspects of the manuscript are the often unclear presentation of the methodology, the lack of background discussing similar methodology, the lack of critical assessment of the results, and the lack of discussion of the limitations of the study. Other more technical but crucial issues were that no shared link or code for the model was provided so it was impossible to check or reproduce the results, and that Gigascience requirements for open data and open code were not met.

Specific Comments

- The scope of the paper is not clearly defined. If this is a paper about a general modelling framework, as stated in some parts, the framework should be presented in its generality. This not done. If this is a validation of a PDAC model, a more rigorous analysis with robust indicators of performance should be provided than those presented.
- The novelty of the study is not clear. Whilst the application to PDAC TME has some novelty, frameworks merging Agent Based modelling and Boolean Networks have already been presented before, and an open access, open source implementation of this framework has been published by Gigascience earlier this year (Voukantsis et al, GigaScience, Volume 8, Issue 3, March 2019, Gigascience Multicellular Systems Biology Series issue 1). The reference to this and other publications on this methodology are missing. They should be added, and explanation of the difference in the methodology between this study and the previous implementations should be provided.
- The authors statement "we developed a modeling framework designed to" is not matched by the publication of the actual framework for others to reuse and benefit from. No mention to open implementation is contained in the manuscript, nor sufficient details are provided to reproduce the implementation.
- The authors answered yes to the question on data and code availability, but no code nor access link to the model (e.g. github repository) were provided, so there is no evidence to support the open data and open code statement.
- The generality of the framework is stated but not demonstrated as only a specific model of PDAC is presented and no general open access environment is provided for other to test, modify and apply to other scenarios.
- The description of the Boolean network (BN) implementation lacks important details. For example, it

is unclear whether the stochastic nature of the Boolean networks relies only on the rate of mutations introduced, and whether the same types of mutations are introduced at the same time. More technically, does the \gamma vector change in each cell?

- It is not clear how the state of each regulatory gene in the BN is defined as a function. The authors statement "determined by the state of its Boolean nodes" (page 6, line 137) is not followed by a detailed description.
- F_j (page 6, line 125) is not well defined. Is it a function (boolean expression) that varies across regulatory nodes and was obtained by optimising the BN to existing data? Is it a PNN ready graph with the boolean functions already calculated? Has there been any fitness of the GRN with PDAC?
- It is unclear whether the update of the networks is synchronous or not.
- Only some aspects of the TME have been accounted for. For example, hypoxia is known to be an important feature of the PDAC microenvironment and is not present in the model, or if it is present, there is not description on this. Furthermore, other details are missing for TME features, for example, no boundary conditions for the diffusion are provided.
- There is no justification on the attempt to fit the expression of the genes to optimize the model parameters. It is well known that expression doesn't necessarily implies changes in the gene network, so meticulous examination is needed to conclude to a specific mechanism as a causal effect.
- It is not clear how the model would transfer to other gene networks or cancers as there is no discussion on functionality that would enable this.
- How the networks plug in with the Agent Based Modelling (ABM) is unclear. A description on this is not provided, so the validity of this aspect cannot be evaluated. If this has been done similarly to previous implementations this should be stated, and an explanation should be provided of how this implementation differs with respect to previous ones. Otherwise, if this is done in a novel way, the methodology for this crucial aspect should be provided.
- The Biocellion software is used as implementation environment. This is not an open source code so this needs to be clarified and addressed as at the moment the work presented in this paper could not be reproduced by the community. Furthermore, the details on how the implementation was carried out within Biocellion are missing, so even users of Biocellion would not be able to implement this simulation and reproduce the results.
- The last paragraph on therapeutic intervention contains very limited results, and no validation, but presents strong conclusions. This should be extended to include validation or omitted.
- The conclusions of the study seem overoptimistic given the overall lack of correlation between the simulation and the PDAC data. A more critical discussion should be presented reflecting this.

Methods

Are the methods appropriate to the aims of the study, are they well described, and are necessary controls included? Choose an item.

Conclusions

Are the conclusions adequately supported by the data shown? Choose an item.

Reporting Standards

Does the manuscript adhere to the journal's guidelines on [minimum standards of reporting?] Choose an item.

Choose an item.

Statistics

Are you able to assess all statistics in the manuscript, including the appropriateness of statistical tests used? Choose an item.

Quality of Written English

Please indicate the quality of language in the manuscript: Choose an item.

Declaration of Competing Interests

Please complete a declaration of competing interests, considering the following questions:

- Have you in the past five years received reimbursements, fees, funding, or salary from an organisation that may in any way gain or lose financially from the publication of this manuscript, either now or in the future?
- Do you hold any stocks or shares in an organisation that may in any way gain or lose financially from the publication of this manuscript, either now or in the future?
- Do you hold or are you currently applying for any patents relating to the content of the manuscript?
- Have you received reimbursements, fees, funding, or salary from an organization that holds or has applied for patents relating to the content of the manuscript?
- Do you have any other financial competing interests?
- Do you have any non-financial competing interests in relation to this paper?

If you can answer no to all of the above, write 'I declare that I have no competing interests' below. If your reply is yes to any, please give details below.

I declare that I have no competing interests

I agree to the open peer review policy of the journal. I understand that my name will be included on my report to the authors and, if the manuscript is accepted for publication, my named report including any attachments I upload will be posted on the website along with the authors' responses. I agree for my report to be made available under an Open Access Creative Commons CC-BY license (http://creativecommons.org/licenses/by/4.0/). I understand that any comments which I do not wish to be included in my named report can be included as confidential comments to the editors, which will not be published.

Choose an item.

To further support our reviewers, we have joined with Publons, where you can gain additional credit to further highlight your hard work (see: https://publons.com/journal/530/gigascience). On publication of this paper, your review will be automatically added to Publons, you can then choose whether or not to claim your Publons credit. I understand this statement.

Yes Choose an item.
