# Supplementary material for: A generalizable data-driven multicellular model of pancreatic ductal adenocarcinoma
Source: Gigascience. 2020 Jul 22;9(7):giaa075. doi: 10.1093/gigascience/giaa075 (PMC7374045; doi:10.1093/gigascience/giaa075)
Supplement: giaa075_Reviewer_3_Report_Original_Submission — Paul Macklin, Ph.D. -- 10/15/2019 Reviewed [file giaa075_reviewer_3_report_original_submission.pdf]

Reviewer Report

Title: A generalizable data-driven multicellular model of pancreatic ductal adenocarcinoma

Version: Original Submission **Date:** 10/15/2019

Reviewer name: Paul Macklin, Ph.D.

Reviewer Comments to Author:

This paper develops new data-driven methodologies to integrate TCGA molecular data int mechanistic multicellular models, with a focus on tumor-stromal-immune interactions in pancreatic cancer. This is a great area of need: there are plenty of great molecular-scale systems models (generally many coupled ODEs), and there are great multicellular models (e.g., systems of ODEs or agent-based models), but it has so far proven difficult to connect the molecular data in TCGA and other resources to the *behavioral* parameters in agent-based and other multicellular population models.
By creating a multiscale agent-based models, the authors were able to systematically investigate the impact on environmental (chemical) factors on the overall tumor growth, as well as identify potential therapeutic perturbations that could improve treatment outcomes. They found that reducing bFGF secretion by stellate cells could increase cancer cell apoptosis to help control or eliminate the cancer cell population. This is a notable, testable model outcome, and it coudl have good implications for combination therapy. Moreover, given that pancreatic cancer is often not detected until it reaches an untreatable state, it poitns to a potential preventative measures (e.g., control stellate bFGF production) that could reduce cancer occurrence in at-risk populations. I also wonder if stellate cells could potentially be a biomarker.
This paper is overall nicely written, and I recommend publication after revision. It makes some very needed advances in connecting molecular-scale data sources (particularly TCGA and multomics data) with multicelular behaviors, including some very nice ways to analyze the models.
I think if the authors more cleanly outlined (and shared source for) their analysis workflow, it ocudl help drive some better standardization of how agent-based models are analyzed, and better tie them to pathology and molecular data. It has a great potential for impact.
Thank you for a very good paper that I think will be a great contribution to GS and the field. I think with minor revisions, it is worth publishing in GS.
Feedback:
1) The literature review is generally comprehensive and inclusive. The authors should be aware of other efforts to integrate larger-scale molecular-scale networks in to agent-based modeling frameworks, including Letort et al's recent work to integrate Boolean signalign networks into agent-based models, such as PhysiBoSS (DOI: 10.1093/bioinformatics/bty766), which combines the MaBoSS open source package for Boolean networks with PhysiCell. Voukantsis et al. wrote a nice article in GS about integrating large gene networks into an agent-based model (DOI: 10.1093/gigascience/giz010).
2) It may be worth mentioning briefly to the readership why Boolean networks are considered instead of ODE networks. (Presumably for efficiency and to reduce the number of parameters.)
3) The authors should more explicitly state (between lines 105 and 111, probably) whether this is a

lattice-based model or off-lattice. Moreover, the method section does not state the movement rules for cells. Are they motile? What are the rules for placement of daughter cells after division? What about cell adhesion and repulsion mechanics? If the cells are static in this model, that's fine, but it needs to be stated clearly.
4) Around line 127, given that signaling factors are diffusion, non-Boolean fields, how do you decide presence / absence of a cytokine for receptors? Are there thresholds?
5) Around line 163, I don't see any cellular uptake of cytokines, even when binding to receptors. Please comment on their model assumptions and possible impact (e.g., on spatiotemporal behavior of cytokine gradients and distributions).
6) The authors state on line 169 that contact-based chemical interaction can be modeled with short diffusion distances lambda = sqrt(D / gamma). This is the diffusion length scale, but numerically, this spatial scale is not goig to be resolved at dx ~ lenght scale (instead of dx ~ 0.1 * length scale). Can the authors discuss why this is fine? (I think it is, since this is just approximating D = 0 and setting an cytokine indicator > 0 nearby for simple contact interactions.)
7) It would be good to include a sample image of an initial spatial distribution. I think it ought to be in the main manuscript, rather that SI. Likewise, I think it woudl be illustrative to readers to show the time series of a single simulation (and to provide a video as supplementary material) to better illustrate the model. (And then the authors will run it in high throughput to generate scalar metrics.)
8) I really like the describe initial state generation. It would be nice of the authors to provide source code for that routine.
9) The model was implemented in Biocellion. Will the source code be shared?
10) It wasn't completely clear to me if cells are proliferating, apoptosing, moving, etc. (Missing details in method). If they are static (e.g., flagged as cycling or apoptotic, but not simulated to the point of dividing or being removed from simulation), this needs to be more clearly stated.
11) It's still not fully clear how you map from genotype to phenotype. Can you please clarify this and perhaps add some brief text? Figure 2 is very hard to follow (the lines cross over lines). Perhaps break out "subfigures" that show which graph nodes map to apoptosis, proliferation, and migration, and how the states of those nodes turn them on? (Linear sum + activation function?)
12) The actual time scale of the simulations was not made clear. How many time steps? How much physical time? DId you simulate a very short time, and hence neglect proliferation, apoptosis, cell growth, cell movement, etc? Please make the time scale
13) The calibration protocol is nicely done. This represents some good work to connect TCGA with behavioral parameters in the ABM.
14) Likewise, I liked your data analysis of the agent models. Many modelers are struggling for the right metrics to analyze their spatial models, and fall back to destroying most of the information to just plot growth curves, etc.
15) I think the authors could improve their impact by (a) providing a nice, clean diagram describing the analysis workflow [showing (i) how one analysis module flows into the next, and (ii) a table of inputs, technique name, outputs, and what's learned for each block in your flow], (b) potentially open sourcing the analysis workflow. It woudl be very beneficial to the field to see the analysis of multicellular models become more standard, much like has happened for molecaulr models.
16) Similarly, the authors coudl have some nice impact in visualizing the calibration protocol, perhaps

similarly to (15).
17) Looking for correlations between calibrated model parameters and PDAC subtypes is novel. It allows us to integrate multiple forms of knowledge (reflected in the model structure) with the data.
18) This paper was a great case for high-throughput computing wtih agent models. May want to look at literature of Gary An who has written quite a lot on this topic
Moreover, the authors might find our most recent work with Argonne National Lab useful for comparison and future use: we built a decision tree binary classifier to divide parameter space into "meets a design objective" (e.g., cancer cell population controlled) vs. "does not meet objective", and adaptively choose parameter sets to refine the decision boundary. This reduced the needed simulations (for our study) from 10^7 to 10^4 simulations per design goal. Due to the decresed cost, we were able to next the design goals to assess the topology of the design parameter space. (DOI: 10.1039/c9me00036d; building on earlier HPC investigations in DOI: 10.1186/s12859-018-2510-x) I think the methods in your work and ours coudl be combined for more adaptive simulation runs and improved data analysis.
19) This is provocative: "For instance, there is a debate concerning whether stroma-cancer interactions are associated with progression of pancreatic cancer or, rather, provide protective measures." To the extent that you can, it would be good to show (and clearly state) that your model identifies ways the same cell types can be both helpful and harmful to the tumor, depending upon context. It's an important point that your model can make.
20) Optionally, you might wnt ot show that the same model, without spatial aspects (well-mixed) gives different results.
Minor points:
1) In the abstract that describes mechanistic models in general, I'd change "These models make the prediction" to "These models can potentially make the prediction" since it describes model outputs that (at that point) have yet to be validated.
2) TGF-beta often renders as TGF-square, which seems to indicate missing fonts somewhere. Please be careful on your manuscript.

Methods

Are the methods appropriate to the aims of the study, are they well described, and are necessary controls included? Choose an item.

Conclusions

Are the conclusions adequately supported by the data shown? Choose an item.

Reporting Standards

Does the manuscript adhere to the journal’s guidelines on [minimum standards of reporting?](#) Choose an item.

Choose an item.

Statistics

Are you able to assess all statistics in the manuscript, including the appropriateness of statistical tests used? Choose an item.

Quality of Written English

Please indicate the quality of language in the manuscript: Choose an item.

Declaration of Competing Interests

Please complete a declaration of competing interests, considering the following questions:

- Have you in the past five years received reimbursements, fees, funding, or salary from an organisation that may in any way gain or lose financially from the publication of this manuscript, either now or in the future?
- Do you hold any stocks or shares in an organisation that may in any way gain or lose financially from the publication of this manuscript, either now or in the future?
- Do you hold or are you currently applying for any patents relating to the content of the manuscript?
- Have you received reimbursements, fees, funding, or salary from an organization that holds or has applied for patents relating to the content of the manuscript?
- Do you have any other financial competing interests?
- Do you have any non-financial competing interests in relation to this paper?

If you can answer no to all of the above, write 'I declare that I have no competing interests' below. If your reply is yes to any, please give details below.

I collaborate with Ilya Shmulevich on mathematical model standards and "digital twins" in cancer care. These collaborations are independent of this paper, and I have had no involvement in the work in this paper. I declare that these collaborations have not affected my impartial scientific review.

I agree to the open peer review policy of the journal. I understand that my name will be included on my report to the authors and, if the manuscript is accepted for publication, my named report including any attachments I upload will be posted on the website along with the authors' responses. I agree for my report to be made available under an Open Access Creative Commons CC-BY license (http://creativecommons.org/licenses/by/4.0/). I understand that any comments which I do not wish to be included in my named report can be included as confidential comments to the editors, which will not be published.

Choose an item.

To further support our reviewers, we have joined with Publons, where you can gain additional credit to further highlight your hard work (see: https://publons.com/journal/530/gigascience). On publication of

this paper, your review will be automatically added to Publons, you can then choose whether or not to claim your Publons credit. I understand this statement.

Yes Choose an item.
